# mRNA Transcriptomics of Galectins Unveils Heterogeneous Organization in Mouse and Human Brain

**DOI:** 10.3389/fnmol.2016.00139

**Published:** 2016-12-16

**Authors:** Sebastian John, Rashmi Mishra

**Affiliations:** Disease Biology Program, Department of Neurobiology and Genetics, Rajiv Gandhi Centre for BiotechnologyThiruvananthapuram, India

**Keywords:** galectin, transcriptomics, neurogenesis, neural stem cell (NSC), brain heterogeneity, molecular neuroanatomy, Allen Brain Atlas, TRANSFAC

## Abstract

**Background:** Galectins, a family of non-classically secreted, β-galactoside binding proteins is involved in several brain disorders; however, no systematic knowledge on the normal neuroanatomical distribution and functions of galectins exits. Hence, the major purpose of this study was to understand spatial distribution and predict functions of galectins in brain and also compare the degree of conservation *vs.* divergence between mouse and human species. The latter objective was required to determine the relevance and appropriateness of studying galectins in mouse brain which may ultimately enable us to extrapolate the findings to human brain physiology and pathologies.

**Results:** In order to fill this crucial gap in our understanding of brain galectins, we analyzed the *in situ hybridization* and microarray data of adult mouse and human brain respectively, from the Allen Brain Atlas, to resolve each galectin-subtype’s spatial distribution across brain distinct cytoarchitecture. Next, transcription factors (TFs) that may regulate galectins were identified using TRANSFAC software and the list obtained was further curated to sort TFs on their confirmed transcript expression in the adult brain. Galectin-TF cluster analysis, gene-ontology annotations and co-expression networks were then extrapolated to predict distinct functional relevance of each galectin in the neuronal processes. Data shows that galectins have highly heterogeneous expression within and across brain sub-structures and are predicted to be the crucial targets of brain enriched TFs. *Lgals9* had maximal spatial distribution across mouse brain with inferred predominant roles in neurogenesis while *LGALS1* was ubiquitously expressed in human. Limbic region associated with learning, memory and emotions and substantia nigra associated with motor movements showed strikingly high expression of *LGALS1* and *LGALS8* in human *vs.* mouse brain. The overall expression profile of galectin-8 was most preserved across both these species, however, galectin-9 showed maximal preservation only in the cerebral cortex.

**Conclusion:** It is for the first time that a comprehensive description of galectins’ mRNA expression profile in brain is presented. Results suggests that spatial transcriptome changes in galectins may contribute to differential brain functions and evolution across species that highlights galectins as novel signatures of brain heterogeneity and functions, which if disturbed, can promote several brain disorders.

## Introduction

Galectins is a unique family of non-classically secreted, β-galactoside binding proteins that has recently received considerable attention in the spatio-temporal regulation of signal lattices, membrane trafficking and in the emergence of several pathologies (Nabi et al., 2015).

Galectins specifically bind to lipids and proteins with a β-galactoside containing headgroup, although with differential affinity and avidity, hence are capable of clustering and organizing dynamic signal lattices on the cell surface through their carbohydrate recognition domains (CRD; Nabi et al., 2015).

To date, 16 mammalian galectin protein members have been identified and all of them lack a recognizable signal sequence for their transport into the classical ER-Golgi cargo trafficking machinery, hence they are proposed to be secreted out into the extracellular environment directly from the cytoplasm. All mammalian galectins have an evolutionary conserved CRD of about 130 amino acids which binds to oligosaccharides *via* recognition of the β-galactoside units (Nabi et al., 2015).

The reported galectins can be further classified into three types on the basis of number of CRDs as proto, chimera and tandem-repeat types. Prototype (gal-1, -2,-5, -7, -10, -11, -13, -14, -15, and -16) are characterized by one CRD, while the chimera-type Gal-3 has one C-terminal CRD and a long N-terminal tail composed mostly of collagen-like repeats that terminate in a short non-collagenous terminal peptide sequence. The tandem-repeat type galectins (gal-4,-6,-8,-9,-12) possesses two CRDs connected by a linker domain of variable lengths that governs several biophysical properties and functions of these proteins (Heusschen et al., 2013; John and Mishra, 2016).

Galectins are known to localize in the extracellular matrix, nucleus, cytoplasm and in the subcellular organelles wherein they are reported to have multifarious roles, however, the surface interactions are predominantly carbohydrate mediated (Dumic et al., 2006). In the recent years, crucial roles of galectins have been examined in organogenesis, cell cycle, apoptosis, migration, adhesion, polarity generation, ciliogenesis, mechanosensing, surface to nuclear signal transport, RNA splicing, adipogenesis and immune system functions (Liu, 2005; Dumic et al., 2006; Baptiste et al., 2007; Sato et al., 2009; Mishra et al., 2010; Rhodes et al., 2013; Nabi et al., 2015).

Both age and diet are known to crucially influence galectins’ expression across different organs (Rhodes et al., 2013). Hence, these oligomeric multifunctional proteins are now emerging as strong regulators of processes ranging from cellular metabolism to complex disease dynamics and carry the potential to emerge as novel nanobiotools and biomarkers for disease therapeutics (Nabi et al., 2015; John and Mishra, 2016).

The first mammalian galectin (RL14.5 or galectin-1) was initially identified in the process of axon pathfinding amongst some other crucial processes (Hynes et al., 1990) and even though galectins’ roles are now being established in several brain disorders such as in neuroblastoma and glioblastomas (Le Mercier et al., 2010; Veschi et al., 2014), dengue fever (Chagan-Yasutan et al., 2013; Toledo et al., 2014), ischemia (Walther et al., 2000; Doverhag et al., 2010; Chen et al., 2014), autism (Voineagu et al., 2011), multiple sclerosis (Stancic et al., 2011), experimental allergic encephalomyelitis (EAE) (Reichert and Rotshenker, 1999) etc., ironically, ‘no systematic studies’ have been performed on its expression, regulation and functions in brain’s normal physiology. This missing gap in our knowledge on brain galectins, could have otherwise established an experimental framework and a precise map for further dissection of the complexity of the mammalian brain and mechanisms of its pathogenesis *via* an understanding of the normal *vs.* abnormal transcription levels.

Since galectins are secreted proteins, their precise spatial localization in terms of expression (producer cells) can be best understood by profiling their mRNA transcriptome. In this context, it is important to determine the distribution of galectin transcripts ‘at all possible levels of expression’ in both single cells and in a population of cells across different regions of the brain, as ‘low level’ transcripts can also act as essential signatures for determining normal and pathological phenotypes.

Since, transcription factors (TFs) play key roles in the tight regulation of gene expression, an understanding of those TFs that regulate various galectins and whose functions in some brain processes are already defined, can enable us to seek ‘first insights’ into the various functions of galectins in brain physiology and their associated pathologies.

**FIGURE 1:**
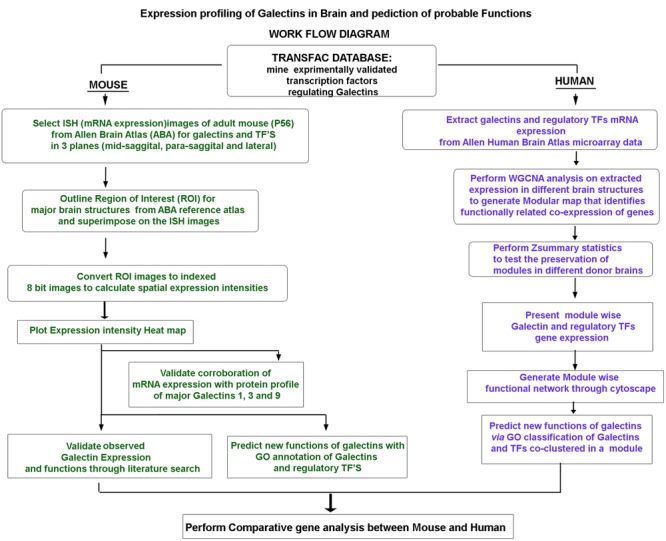
**Flowchart of the experimental strategy: The summary of the experimental logic followed for (i) mRNA expression profiling of galectins in mouse and human brain, (ii) validation of galectins expression and (iii) prediction of probable functions of galectins in brain *via* GO analysis based mining of the known functions of the putative regulatory transcription factors**.

To this end, as described in workflow (**Figure 1**), the Allen Brain Atlas, provided the raw transcript data on galectins’ expression in young adult mouse brain and the microarray data of human brain, while the TRANSFAC software enabled the identification of putative TFs that can regulate galectins. Hence, we extracted, normalized and quantified the galectin gene expression data and clustered it *via* separate algorithms for mouse and human to identify the linkages among galectins and also with their regulatory TFs in different regions of brain. Given the fact that ‘weakly expressing’ transcripts are crucial in brain’s highly tuneable plasticity, our proposed method of quantitation of expression was necessary as it picked up even the sparser areas with low intensity expression which were hidden or not clearly visible in the expression mask provided by the Allen Institute (please see Supplementary Figure S15). It is to be noted that Allen Brain Atlas developers encourage such ‘end user’ data curation as their expression masks are created on the general methodology of ‘one size fits all’ basis and may not be highly useful for every research question (Jones et al., 2009).

Our analysis identified low to moderate expression of various galectins in the neurons of the olfactory bulb, cortex, hippocampus, basal ganglia, thalamus, hypothalamus, amygdala, substantia nigra and the cerebellar cortex. Besides, the brain regions associated with adult neurogenesis-the olfactory bulb, rostral migratory stream (RMS), lateral ventricles, subventricular zone (SVZ), sub-granular zone and the dentate gyrus (DG) showed heterogeneous galectin expressions. All galectins also had a highly heterogeneous expression within and across brain sub-structures and were identified (through informatics prediction) to be the regulatory targets of crucial brain enriched TFs that have been previously reported for their distinct roles in brain activities such as proliferation, differentiation, synaptic transmissions and neuronal survival. Hence, informatics driven Galectin-TF cluster analysis, gene ontology (GO) annotations and data driven co-expression networks enabled prediction of functional relevance of galectins in the neuronal processes.

The detailed microarray-based analysis of human transcriptome of individual galectins revealed some diverse and several other conserved modules between brain regions within and among the individual human subjects. However, significant differential galectin signatures were observed for putatively homologous brain regions in mouse and human. For example, while *Lgals9* (ENSEMBL gene denotion norms: ‘*Lgals’* for mouse and ‘*LGALS’* for human) had maximal spatial distribution across brain in mouse, with predicted predominant roles in neurogenesis; *LGALS1* was ubiquitously expressed in human. Limbic regions which are associated with learning, memory and emotions and substantia nigra (SN) which is associated with motor movements also showed strikingly high expression of *LGALS1* and *LGALS8* in human *vs.* mouse brain. The overall expression profile was mostly preserved across both these species for galectin-8, however, galectin-9 showed maximal preservation only in the cerebral cortex.

This study, hence, provides a data and informatics driven resource on galectin family genes in terms of their relation to neuronal cell diversity and functions within and across species. It highlights both species specific and across species galectin expression patterns that supports their probable roles in the evolutionary divergence on one end and conservation of some basic functional modules on the other. This work further lays a platform for research on galectins’ probable roles in the brain physiology and pathogenesis, based on the modulation of their transcriptome dosages by the regulatory TFs.

## Materials and Methods

### ISH Images of Mouse Brain

The *in situ* hybridization (ISH) raw high resolution images were downloaded from the ABA Atlas for each galectin in three different planes (lateral, para-sagittal and mid-sagittal from http://mouse.brain-map.org/ (please see Supplementary Table S1 that provides ABA URL’s list for each galectin ISH images used for analysis). The reference atlas and Nissl’s stained images synchronous or corresponding to the respective ISH images were further downloaded. This study has been performed strictly according to the ABA ‘citation’^1^ and ‘terms and use’^2^ policy. Please note that averaging over at least 2–3 good quality images in each plane of section was done to generate the expression profile (Lein et al., 2007).

A good ABA ISH image was defined by (i) anatomic normalcy, (ii) dissection quality, (iii) section orientation, (iv) signal-to-noise ratio, (v) focused image, (iv) uniform illumination of sample during image acquisition, (vii) absence of tissue artifacts (bubbles, tears, tissue folds and debris) and (viii) uniform white balance or background correction^3^ (follow ‘Documentation’ Tab-Supplemental Data-ABA Data Production Processes and ISH Platform Controls). According to these criteria, galectins’ predicted regulatory TFs-CEBPA, RNF96/TRIM28 and TCF3 failed ISH quality control and this was directly mentioned on ABA Image display platform. In addition, GLI3, NANOG, TAL-1, REX1/REXO1 and ZFX image series showed 1) non-uniform probe hybridization leading to variation/inconsistency in signal (color) in multiple adjacent sections and/or 2) inconsistent hybridization due to presence of sectioning artifacts such as bubbles, debris, tears and folds in tissue in ‘multiple’ sections, hence these TFs were also eliminated from further analysis (*see* Supplementary Table S3). In general, wherever there were occasional tissue artifacts, bubbles or folds, adjacent images were used as a representation of a good quality image.

### Expression Analysis

The ISH process is not strongly quantitative in the sense of measuring transcript copy number; hence in order to detect the extent of regional expression of galectins and their putative regulatory TFs, an expression signal detection schema was devised (*see* Supplementary Figure S16 for detailed explanations with examples). First the high resolution ABA images were converted to 8 bit format using freely downloadable-64 bit Fiji image analysis software [it’s just ImageJ] (Schneider et al., 2012). This 8 bit image format is associated with a signal intensity gradient (LUT) in grayscale with pixel intensity values ranging from highest intensity (*index position 0, shade: black*) to lowest (*index* position *255, shade: white*). When images are visualized in this LUT, the signal will be black or in shades of gray against white background. In order to better distinguish the differences in pixel intensities corresponding to gene expression and to visually describe it, the grayscale LUT with 256 indexed positions was custom converted to pseudocolor mask to visualize the expression profile for each image. The pseudocolor or LUT (Look Up Table: calculated values of output color data as it corresponds to the input RGB color data) was created by editing the colors at each index position (Fiji: Image > Type > Color > Edit LUT > Color entry window option) generating discrete seven-color scale with decreasing level of expression (from ‘*very very high’ to ‘very very low’ till ‘ no signal*’: red–dark orange–orange–yellow–green–blue- dark blue–black). The following RGB values were entered in the ‘Entry window’ for each color: red (255,0,0); dark orange (255,124,0); orange (255,190,0); yellow (255,255,0); green (0,255,0); blue (0,123,255); dark blue (0,0,255) and black (0,0,0); However, note that the false color LUT does not change the underlying grayscale pixel value, which is measured for scoring expression intensity.

A series of decreasing integers from 7 to 0 were then assigned to the above color codes to generate a calibration scale and custom pseudocolor LUT, that is (i) ‘*very very high*’/very strong expression (color: red, index position: 0–31, pixel intensity scale = 7), (ii) ‘*very high’*/strong expression (color: dark orange, index position: 32–63, pixel intensity scale = 6); (iii) ‘*high*’/above moderate expression (color: orange, index position: 64–95, pixel intensity scale = 5; (iv) ‘*medium’*/moderate expression (color: yellow, index position: 96–127, pixel intensity scale = 4); (v) ‘*low’*/below moderate expression (color: green, index position:128–159, pixel intensity scale = 3); (vi) ‘*very low’*/*weak expression* (color: cyan, index position:160–175, pixel intensity scale = 2); (vii) detectable but ‘*very very low’ expression* (color: blue, index position:176–191, pixel intensity scale = 1); (viii) ‘*no expression/no signal’* (color: black; index position:192–255, pixel intensity scale = 0). This pseudocolor LUT was saved as a Fiji plug-in and all ISH 8 bit converted images were edited using the same LUT (in LUT Editor) to visualize differential expression signal for each gene. Note that equal ‘intensity’ index positions, that is a total of 32 positions, were assigned for scales from ‘*very high*’ expression (red, intensity scale = 7) to ‘*low’* (green, intensity scale = 3) in LUT Editor. The next 32 index positions below ‘low/green’ were equally divided into 16 positions each to distinguish weak signals into ‘*very low’* and ‘*very very low’* pixel intensities. Last 64 index positions (from 192 to 255) were assigned for ‘*no signal’* as these mainly positions corresponds to background intensities in 8 bit gray scale format.

In each image, individual structures were annotated based on the ABA reference atlas ontology and images were mapped/marked into regions of interest (ROIs, Fiji: Analyze/Tools/ROI Manager) according to the reference atlas ^4^. The pseudocolor image conversion helped in identifying even the extremely low level intensity segments and regions. It also enabled visualization of cohorts/clusters of more intensely expressing pixels in a region of overall low expression. Hence, the qualitative details of the visual description of galectin expression were enhanced. The pseudocolor scale also aided in quantitative description of the expression signal as ‘heat maps’ (**Figure 5**) (*see Supplementary Datasheet S4: Supplementary Figure S16/PowerPoint document for further details and examples for pseudocolor LUT generation and use of color LUT for expression intensity scaling. Also see steps for scaling density and derivation of expression factor-which is represented in heat-maps)*.

To determine expression levels, let’s say of *Lgals8* in Cortex Layer 5 (L5) in parasagittal plane, where the ROI shows ‘a’ cells as blue, ‘b’ cells as green and ‘c’ as yellow and ‘d’ cells in red, mean gray value associated with these pseudocolor pixels was ascertained from 8 bit binary images using Fiji: Image/Threshold and Analyze/Set Measurement (click on mean gray value and max and min gray value to visualize gray value range)/Measure tool option to measure the mean gray values around all expressing pixels in the selected region. Let’s say it was 169.18 (ranges from minimum 0 to maximum 255), then expression level (L1) according to above mentioned intensity scale (from 7 to 0) will receive value of 2 (*very low*). On performing similar measurements over three images, in one plane of section, an average Intensity [L, L = L1 + L2 + L3)/3] can be obtained. Similar exercise can be followed to yield average intensity in other planes of sagittal sectioning (mid/lateral as the case maybe).

In addition, ‘density’ which is the distribution of positively labeled cells or pixels in a given area over the total number of cells or pixels present in that area was also considered in analysis. Fiji tools: Image (8 bit)/Adjust/Threshold; Process/Binary/Make Binary/Watershed/; Analyze/Tools/ROI (assign ROI) were used followed by Analyze/Measure Particle option for counting number of positively expressing pixels. ROI selected in Nissl’s image to mark the Region of Interest (corresponding to relevant region in the ABA reference atlas) was superimposed on the ISH image for area normalization. When sum of the areas of all positive signals (obtained from Fiji) was divided by the area of the smallest pixel, that is 0.17 μm^2^ (in Fiji), then the number of total pixels for the transcript of interest and total number of pixels available in that area (through Nissl’s image) were independently obtained. From this, ‘density’ in percentage was calculated (% density Np, Np = [(sum of areas of all pixel intensities for a transcript in a ROI/0.17)/(sum of areas of all pixel intensities for Nissl’s image in the same ROI/0.17) ^∗^100]). The calculated density (in percentage) was further classified on the scale of 1–4: (a) scale of 1: 0–5% (*sparse distribution*); (b) scale of 2 = 5–20% (*scattered distribution*); (c) scale of 3 = 20–70% (*medium distribution*) and (d) scale of 4 = > 70% (*high/wide-spread distribution*). For example, say in a *Lgals8* ISH image, ROI of parasagittal plane Cortex Layer 5 (CTX-L5) showed 440358.8 positive pixels while Nissl’s image showed 9219235 positive pixels in the given ROI, then the area density is 47.76% and receives a density (D1) value of 3 (*medium distribution*). On performing similar measurements over three images, in one plane of section, an average Density [D, D = (D1 + D2 + D3)/3] can be obtained. Please note that this kind of analysis was done for each plane of sectioning using multiple magnification levels to accurately quantify the expression. Also note that for more dense regions, a robust manual image thresholding was performed using Fiji (*see* help files in Fiji).

In this way, the gene expression in each area can be subjectively scored according to two parameters: (a) the level of Intensity (L) and (b) the level of Density (D), i.e., the amount of area covered by each level of intensity. This methodology was borrowed from the previously published protocols with a slight modification (Lein et al., 2007; Sunkin and Hohmann, 2007; Dahlin et al., 2009).

Different intensity level within an area were averaged (2–3 images in a sagittal sectioning plane) and multiplied with the average of density level within an area to provide an average expression factor (E) for one specific area or sub-structure in the mouse brain. This can be further represented by the following relationship:

(1) $$\begin{array}{c} \text{E}\;\text{=}\;\text{L}\;\;\times \;\;\text{D}, \end{array}$$

where L is average of different intensities and D is an average of respective different densities.

Since there are seven intensity levels and four density levels, the upper limit for average expression factor in individual subregions was 28 and the lower limit was 0. These expression factors were further assigned the following categories: ‘*very low*’ (where, 0 < E < 6), ‘*low*’ (where, 6 < E < 11), ‘*moderate*’ (where, 11 < E < 17), ‘*high*’ (where, 17 < E < 22) and “*very high*” (where, E > 22).

Hence, using the average values of Intensity (L) and Density (D) functions in the above examples for CTX L5 ROI, where say intensities (L1, L2, L3) for three images are 2,2,1 and densities (D1, D2, D3) are: 3,3,3 then expression factor E = [L1xD1 + L2xD2 + L3xD3/3] will be 5. So, in this example, expression factor for CTX L5 for *Lgals8* will be categorized as overall ‘*very low’* (as 0 < E < 6).

Please see Supplementary Table S4 for detailed expression analysis (heat maps). Note that expression factor of a house keeping gene GAPDH is shown to highlight the relative expression of ubiquitous gene in comparison to galectins and their predicted TFs in various brain subregions).

Please see Supplementary Method file for further details and references on (i) Prediction of potential TFs regulating galectins in mouse and human, (ii) Structures studied in mouse brain (iii) Expression heatmaps, (iv) GO annotation, (v) Microarray analysis from Human Allen Brain Atlas and (vi) Network Construction. To access Allen Brain Atlas white pages and documents, please see URL: http://mouse.brain-map.org/ and follow ‘Documentation’ section in Tabs.

## Results

### Galectin Family Gene Expression Analysis in Mouse Brain

To begin, the original ISH image data from the ABA was identified and extracted for galectin-1,2,3,4,7,8,9, and 12 (Supplementary Table S1). The remaining galectin genes were not detected in the ABA dataset.

**FIGURE 2:**
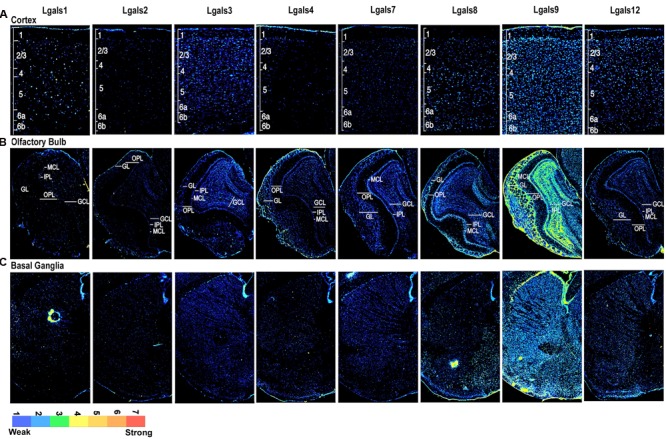
**Galectins expression in mouse Cerebral Cortex, Olfactory Bulb, and Basal Ganglia (A)** Expression profile of galectins in cortex with individual cortical layers marked from L1 to L6b. **(B)** Expression profile of galectins in olfactory bulb (OB), with different cell layers labeled as GL, glomerular layer; IPL, inner plexiform layer; OPL, outer plexiform layer; MCL, mitral cell layer and GCL, granule cell layer. **(C)** Expression profile of galectins in basal ganglia (BG).

**FIGURE 3:**
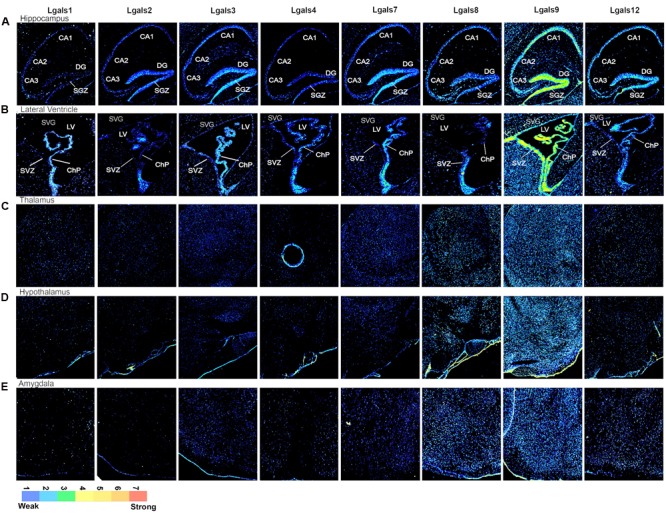
**Galectins expression in mouse Hippocampus, Sub-Ventricular Zone, Thalamus, Hypothalamus and Amygdala (A)** Expression profile of galectins in hippocampus. CA1, *Cornu Ammonis*1; CA2, *Cornu Ammonis*2; CA3, *Cornu Ammonis*3; DG, dentate gyrus; SGZ, sub granular zone. **(B)** Expression profile of galectins in lateral ventricle (LV). SVZ, sub ventricular zone; ChP, choroid plexus. **(C)** Expression profile of galectin in thalamus. **(D)** Expression profile of galectins in hypothalamus. **(E)** Expression profile of galectins in amygdala.

**FIGURE 4:**
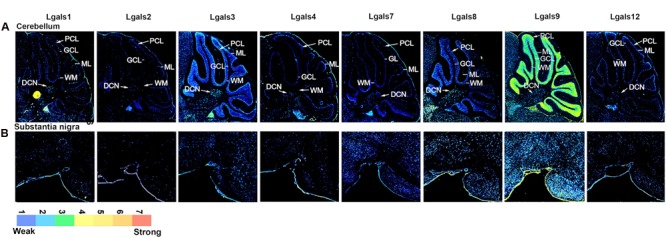
**Galectins expression in mouse Cerebellum and Substantia nigra (A)** Expression profile of galectins in cerebellum. DCN, deep cerebellar nuclei; WM, white matter; ML, molecular layer; GCL, granular cell layer; PCL, Purkinje cell layer. **(B)** Expression profile of galectins in substantia nigra.

**FIGURE 5:**
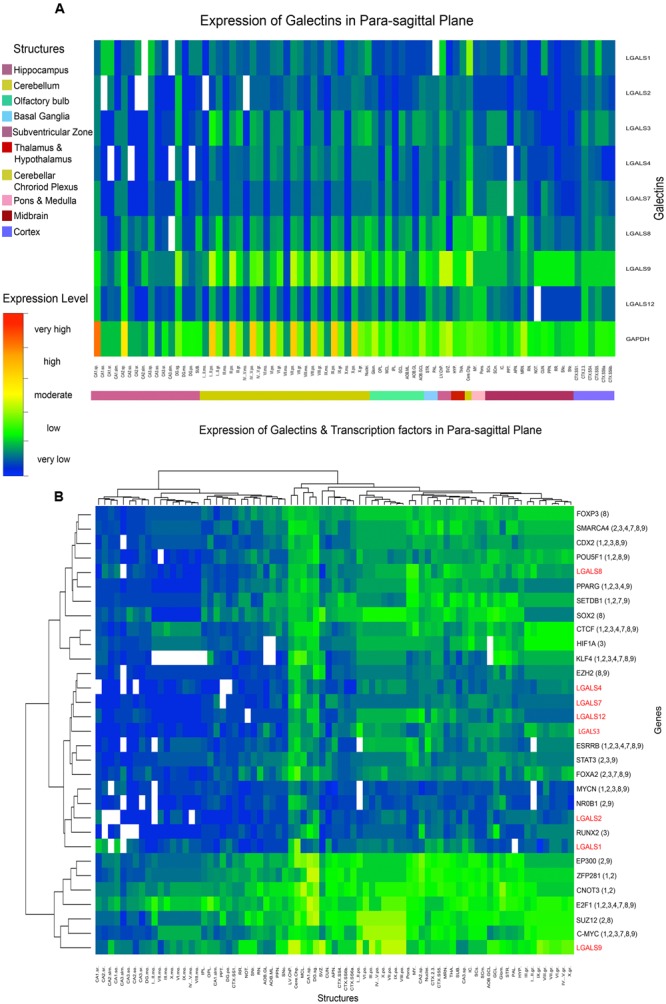
**Heat map of Galectin-TF gene expression in Para-sagittal plane (A)** Heat map of galectins in mouse showing the average expression in each sub-structure arranged by parent structures. Expression factor of GAPDH is shown as a reference. **(B)** Heat map of galectins with their putative regulatory transcription factors showing expression correlations after hierarchical clustering at the level of both gene and structure. Symbol ‘LGALS’ is used instead of ‘lgals’ ENSEMBL denotion for mouse galectins only for uniformity of labeling.

**FIGURE 6:**
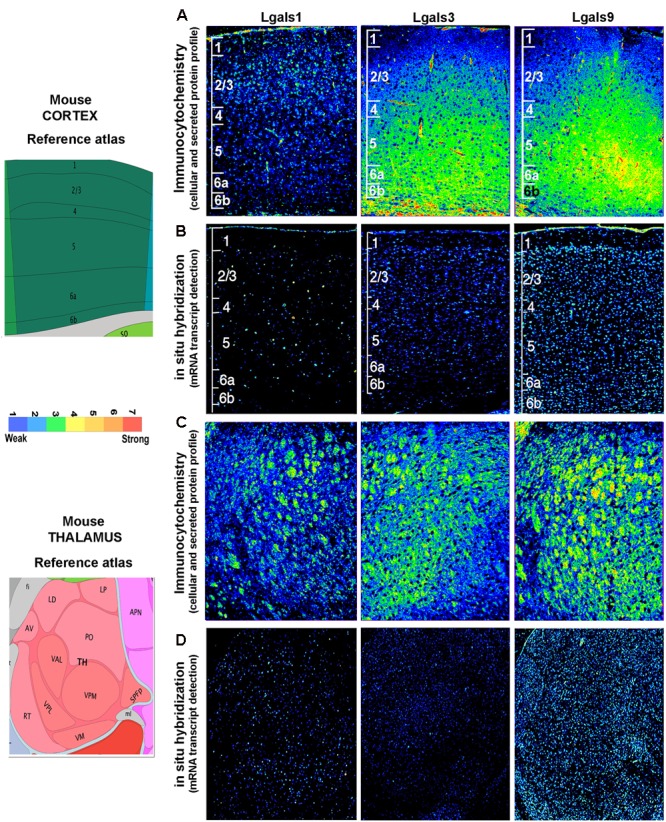
**Comparison and validation of galectins’ mRNA expression with protein profiling in mouse Cerebral cortex and Thalamus. (B,D)** ABA based mRNA expression profile of 3 major galectins in cortex and thalamus was compared with immunohistochemistry based protein expression in the respective regions as shown in **(A,C).** The individual cortical layer are marked from L1 to L6b and pseudo-color calibration bar is included to represent the intensity of expression. Note a good regional corroboration between mRNA and protein expression except that the protein expression is more widespread due to the secretory nature. The protein is also detected in the extracellular space which is suggestive of autocrine-paracrine effects.

**FIGURE 7:**
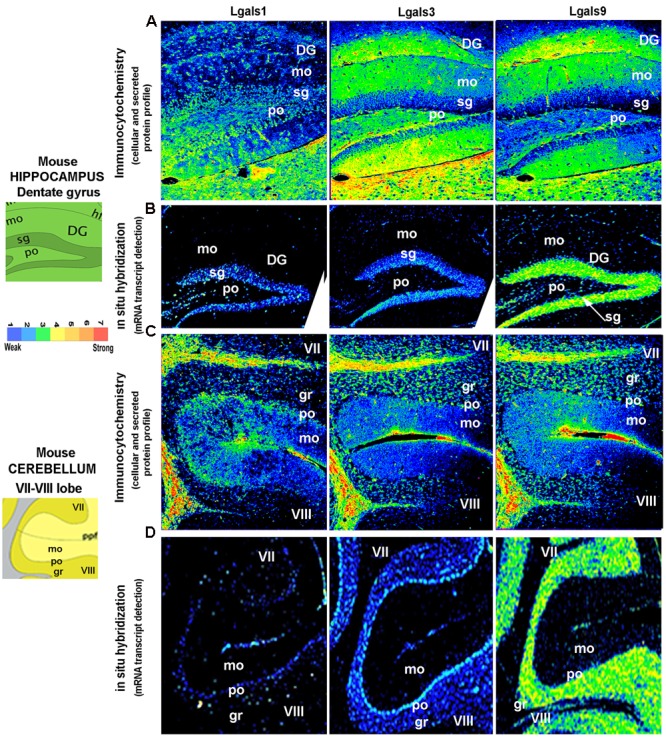
**Comparison and validation of galectins mRNA expression with protein profiling in mouse Hippocampus and Cerebellum. (B,D)** ABA based mRNA expression profile of 3 major galectins in the Dentate Gyrus region of the hippocampus and VII–VIII lobe of the cerebellum is compared with immunohistochemistry based protein expression in the respective regions as shown in **(A,C).** DG, dentate gyrus; mo, molecular layer; sg, sub-granular layer (a zone of adult neurogenesis); po, polymorph layer in DG and Purkinje cell layer in cerebellum; gr, granule cell layer; VII/VIII, 7th and 8th lobe of cerebellum with prepyramidal fissure(ppf). Pseudo-color calibration bar is included to represent the intensity of expression. Note a good regional corroboration between mRNA and protein expression except that the protein expression is more widespread due to the secretory nature. The sub-granular zone of the DG that expresses the transcript is essentially low in intracellular protein pool, probably due to secretion. The protein is also detected in the extracellular space and on surface of cells is suggestive of autocrine-paracrine effects.

**FIGURE 8:**
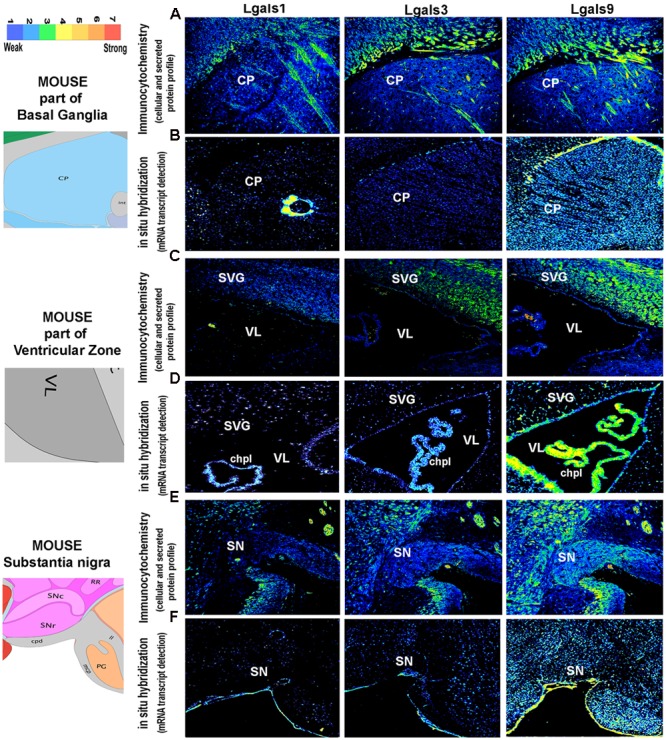
**Comparison and validation of galectins mRNA expression with protein profiling in mouse Basal ganglia, Ventricular Zone and Substantia nigra. (B,D,F)** ABA based mRNA expression profile of 3 major galectins in the Striatal Caudoputamen (CP, site of activity dependent synaptic plasticity, motor control and procedural memory) region of the basal ganglia, Ventricular zone: VL (in the vicinity of neurogenic subventricular zone, SVG; and choroid plexus: chpl) and Substantia nigra (SN, motor control region) is compared with immunohistochemistry based protein expression in the respective regions as shown in **(A,C,E)**. Pseudo-color calibration bar is included to represent the intensity of expression. Note a good regional corroboration between mRNA and protein expression except that the protein expression is more widespread due to the secretory nature. The protein is also detected in the extracellular space and on the surface of cells is suggestive of autocrine-paracrine effects.

Parallel to this process, a run through TRANSFAC software enabled us to predict TFs that could regulate galectins’ transcription (Supplementary Table S2). Through this analysis, a total 34 TFs, for all the galectins combined, were identified in mouse (Supplementary Table S2). Then with the help of literature survey, GO annotations and UniProt (2012), evidences for the involvement of these TFs in the nervous system processes were examined. Out of the 34 predicted TFs, experimental evidences for only 30 could be established for some involvement in the nervous system functions (Supplementary Table S3). The ISH image data for the 30 TFs were searched in the Allen Brain Atlas (ABA) and out of these TFs, only 24 were found to have good quality images (Supplementary Table S3). Upon completion of this data mining exercise, the ABA sagittal section images for galectins and their putative regulatory TFs were converted into ‘8 bit indexed images’ for quantitation of expression in various brain regions and across different planes of brain sections, i.e., mid, para and lateral **(***see Materials and Methods*, **Figures 2**–**4**; Supplementary Figures S1–S12, Supplementary Table S4). The intensity of expression was represented by the pseudocolor LUT converted images with a calibration bar **Figures 2**–**4**) and the expression factor was represented by the heatmaps (**Figures 5A,B**, Supplementary Figures S13 and S14, Supplementary Table S4). Our expression mask or pseudo-colored images were found to be much in concert with the protein expression profile of galectins than the one given by the Allen brain atlas (*for an example see* Supplementary Figure S15 which shows a comparison between mask provided by the Allen brain atlas, mask created by our methodology and our antibody mediated detection of galectin-12 protein. Please note that the same plane of section, same age, same gender and same mouse strain has been used for precise comparisons. Of note was the observation that the regional protein expression data of galectin-12 matched well with our customized mask for transcript expression. This was further confirmed for *Lgals1*, -3 and -9 (**Figures 6**–**8**). Also note that due to secretory nature of galectins, protein expression is more widespread in comparison to respective transcript profiling of the producer cells).

### Regional Expression Profile of Galectins in Mouse Brain

To understand the expression dynamics of galectins and their transcriptional program, we chose to study each structure in mouse brain individually to determine the cellular context of their expression.

As shown in **Figure 5B**, to get correlated genes, galectins and their putative regulatory TFs were further arranged in clusters after performing the hierarchical clustering. As many of the galectins were regulated by multiple similar TFs (Supplementary Table S3), matching the pattern of TFs with respect to individual galectin expression further helped us in making a fair assessment regarding their common functions.

Hence, we first describe the matched expression profile of galectins and their putative regulatory TF’s observed in different structures in the adult mouse brain and then discuss the possible roles of galectins, which were predicted with the help of region wise expression and GO (Supplementary Table S5).

From the initial observations of all ABA galectin images (**Figures 2**–**5**), *Lgals9* showed the highest expression at the whole brain level and even individually in the major brain structures as compared to other galectins. The finer expression details are described as follows:

### The Cerebral Cortex

The cerebral cortex is known to co-ordinate the sensory and motor information. Anatomically, layer 1 (L1), situated below the meninges, consists of few neuronal cell bodies, layer 2/3 consists of granule cells and small pyramidal cells, layer 4 consists of granule cells, layer 5 is enriched in large pyramidal cells and layer 6 in fusiform cells (Supplementary Figure S4, see reference atlas). In particular, layer 4 along with layer 2/3 is associated with experience dependent plasticity. Layer 4 receives inputs from outside cortex and distributes it to other cortical layers for further processing, whereas layer 5 neurons are majorly involved in voluntary motor movements.

Following the layer-wise schema, we find that *Lgals9* had a widespread expression in layers 2–6 (L2–6) of cortex except in L1 where it showed very low expression probably due to the sparseness of cells which were majorly only inhibitory neurons (**Figures 2A** and **5A**; Supplementary Figure S1). While *Lgals9* and *Lgals12* showed ‘low’ expression in L2/3; galectins-1, 2, 3, 4, 7, and 8 showed ‘very low’ expression in the same region. *Lgals9* was the only galectin with below moderate expression intensity in L4 while rest of the galectins had low to very low detectable expression intensities. Layer 5 pyramidal cells expressed galectin-1 and 9 followed by galectins-3, 8, and 12. *Lgals1* showed the strongest expression intensity in L5 among all the layers but this intensity was probably due to a few cells that expressed this galectin very strongly. Also, a few *Lgals4* positive cells were detected in L5-6.

In the galectin-TF co-expression analysis, E2F and EP300, that were predicted to regulate *Lgals9* (Supplementary Tables S2 and S4, Supplementary Figure S4) showed a similar pattern of expression to *Lgals9* but with intensities in a different order of magnitude (**Figure 5B**). The expression pattern for *Lgals8* in L5 matched with that of SMARCA4, MYC, and EZH2, which were some of the TFs that possibly regulated *Lgals8* (Supplementary Table S2). This observed diversity in the expression levels of galectins in the cortex could be due to the diversity and heterogeneity in the distribution of inhibitory interneurons (Markram et al., 2004) and also may be due to the complexity of the cortex itself.

### The Olfactory Bulb (OB)

The OB transmits smell information from the nose to the brain and also receives information from the amydala, neocortex, hippocampus, locus coeruleus and substantia nigra for odorant/chemical awareness. This input circuitry also enables odorant associated memory formation for future behavioral responses. Glomerular layer (GL) is the first level of synaptic processing in OB and the spatial odor maps organized by chemicals in this layer can be used for perception of odor in the olfactory cortex *via* the participation of the outer plexiform layer (OPL), the mitral cell layer (MCL), the inner plexiform layer (IPL) and the granule cell layer (GCL) in OB. OB is also one of the major sites of adult neurogenesis (*please see* Supplementary Figure S1 *for diagrammatic representation of OB layers*).

We identified the presence of atleast moderate expression for *Lgals9* in the OB (**Figures 2B** and **5A** Supplementary Figure S1B, Supplementary Table S4) in comparison of all other galectins. *Lgals9* showed moderate expression intensity in the GL, the MCL and the GCL due to a few cells that had strong expression in these layers in comparison to low expression intensity of cells in the OPL and the IPL. Overall, *Lgals9* also had the highest average expression in the GCL of OB amongst all the galectins and TFs examined (Supplementary Figure S5, **Figure 5A**). The distribution and low intensity expression of *Lgals8* was apparent in the MCL, accessory olfactory bulb’s granular cell layer (AOB GCL) and the GL. *Lgals1* and *Lgals2* showed no major expression but still there were few cells which were positive for both these galectins in OB. Galectin-3,-4,**-7**, and 12 showed very weak positivity throughout OB.

Among all the predicted TFs which could regulate *Lgals9* expression; E2F1, EP300, SETDB1, CTCF, POU5F1 were more or less moderately expressed in the GCL. *Lgals8* expression in MCL also corroborated well with the positive transcript signal for many putative TFs regulating its expression, for example, SMARCA4, SOX2, MYC, FOXP3, KLF4, and CTCF (Supplementary Figure S5; **Figure 5B**). For other galectins, no interesting galectin-TF co-expression was identified.

### The Basal Ganglia

The basal ganglion is associated with the control of the voluntary movements, procedural learning, routine behavior, cognition and emotions.

The basal ganglion comprises of striatum and palladium and most of the cells in striatum are GABAergic. Circuits of basal ganglia that have been shown to be responsible for motor control include excitatory inputs from the cortex, especially from L2/3 and L5, intralaminar nuclei of the thalamus and also from the SN pars compacta toward the striatum. Through the direct pathway, striatum output projects to globus pallidus interna and SNr, while *via* the indirect pathway, they project to globus pallidus externa, which are the two parallel cortex-basal ganglia-thalamus-cortex circuits (Kreitzer and Malenka, 2008).

In our study, the striatum and palladium showed comparable levels of *Lgals9* (**Figures 2C** and **5A**). A similar pattern was also observed for *Lgals9* putative regulatory TF E2F1 and to a certain extent for EP300 (Supplementary Figure S6, **Figure 5B**). *Lgals8* and *Lgals12* also showed enough number of cells with average expression intensity.

### The Hippocampus

Hippocampus is made up of the DG and the Ammon’s horn (or *Cornu Ammonis*- CA and is subdivided into CA1, CA2, and CA3). DG is a major site of adult neurogenesis and hippocampus is known to play major roles in consolidation of short and long term memories as well as spatial navigation.

*Lgals9* showed a ‘wave like expression’ pattern in the hippocampal subregions with moderate expression in CA1, a decrease in expression in CA2 and with a slight increase in CA3 (**Figure 3A**, Supplementary Figure S2A) The DG pyramidal neurons were prominently labeled with *Lgals9* in some cells near the base of the granular cell layer (GCL) in sub granular zone (SGZ), a region defined by the presence of more committed neural stem/progenitor cells. DG overall showed the strongest galectin expression across all the hippocampal subregions with *Lgals9* dominating the galectins. For all other galectins, although the intensity levels in DG were weak, there were few pyramidal neurons which had below moderate expression in SGZ. Similarly, a few cells with moderate expression level were identified in the polymorph layer of DG for galectins-1, 8, 9, and 12. The molecular layer of DG had weak intensity expression for *Lgals9* which was far weaker for all other galectins. Pyramidal layer (sp) of CA2 showed below moderate expression intensity for *Lgals9* and *Lgals12* which was followed by CA1 sp layer’s with approximately moderate expression for the same galectins, hence a gradient pattern of expression in the CA region of the hippocampus was delineated. *Lgals1* and *Lgals8* showed moderate expression in the CA3 pyramidal layer and upon comparing the expression levels in the subiculum (SUB), *Lgals8* showed the strongest expression among all the galectins (**Figures 3A** and **5A**).

Amongst the predicted TF’s that could regulate galectins in brain, E2F1, EP300 and MYC were strongly expressed in the Ammon’s horn (Supplementary Figure S7, Supplementary Table S4, **Figure 5B**) and these TFs formed a cluster of their own with *Lgals9* having a similar expression pattern for CA1, CA2 to CA3 (**Figure 5B**). The other putative regulatory TFs for *Lgals9* that showed moderate expression were POU5F1, FOXA2, SETDB1, CTCF and SMARCA4 (Supplementary Figure S7, Supplementary Table S4, **Figure 5B**). Although these TFs did not form a cluster with *Lgals9*, they did show a good expression level, similar to *Lgals9*. Interestingly, expression profile for *Lgals9* in the GCL of SVZ was observed to be similar for its possible regulators, i.e., E2F1, EP300, SMARCA4 and SETDB1.

Although a lot of putative TFs that regulate *Lgals2* had a good expression in the hippocampus, its own expression was found to be very low which suggests that it may be a low level and cell-type specific transcript (**Figures 3A** and **5A**). *Lgals3*, however, had a good cellular positivity but the intensity weakened as we moved along CA1–CA3 direction. The TFs HIF1A and RUNX2 were predicted to regulate *Lgals3* and were also found to be co-expressed in the Ammon’s horn in the ISH images (Supplementary Figure S7). Few cells in CA1 and DG showed strong intensities for HIF1A while RUNX2 was slightly weakly expressed throughout the Ammon’s horn. The expression of RUNX2 in DG, however, matched with that of *Lgals3* at the base (Supplementary Figure S7; **Figure 5B**). Putative TFs, SOX2 and FOXP3 both of which may specifically regulate *Lgals8*, were also present in the cluster with it, providing solid assumption for their regulatory effects on *Lgals8* (**Figure 5B**).

### The Lateral Ventricle and the Subventricular Zone

Subventricular zone that lines the lateral ventricles is well documented to be enriched in the neural stem cells. The choroid plexus (ChP) which protrudes into the lateral ventricles and into the fourth ventricle near the cerebellum is responsible for secreting the cerebrospinal fluid (CSF) (Brown et al., 2004) and maintains the outer blood brain barrier (Hilhorat et al., 1973). This system of ChP-CSF is important for the development of CNS. ChP is an area which provides access to immune cells into the CNS and it is composed of large number of T cells (Lun et al., 2015). Previous studies have suggested its role in inflammation (Schwerk et al., 2015), ischemia (Doverhag et al., 2010), Alzheimer’s (González Marrero et al., 2015) and Multiple Sclerosis (Stancic et al., 2011).

*Lgals9* showed moderate to high expression in the SVZ and ChP (**Figures 3B** and **5A**, Supplementary Figure S8). There was a heterogeneity of galectins in the SVZ with a few cells expressing strongly for *Lgals3* (Comte et al., 2011) and *Lgals12*. The wall opposite to SVZ in lateral ventricle was also positive for expression of galectin-9, -2, -3, and 4.

Moderate to high expression of *Lgals9* in ChP suggested its various roles in ChP associated immune diseases and indeed *Lgals9* function has been widely documented in the immunological responses (Wiersma et al., 2013). Other galectins also had positive expression in ChP but it was of low intensity. However, *Lgals1* showed a below moderate expression.

Some of the TFs which may regulate *Lgals9* also had moderate expression levels in SVZ like E2F1, KLF4, and EP300 (Supplementary Figure S8, **Figure 5B**, Supplementary Table S4) while some of them had weaker expression intensities like MYC, SETDB1, POU5F1, FOXA2 and some were present only in few cohorts of cells that were moderately positive, as was the case with EZH2. *Lgals3* showed an overall weaker expression in SVZ where its putative regulatory TFs, FOXP3 and POU5F1 had the same expression but SOX2, KLF4 and SUZ12 had a higher expression. In this region, a few cells, weakly expressing galectin-1, - 2, -4, and 7 were also identified with their putative regulatory TFs, i.e., CTCF, ESRRB, PPARG which had similar pattern of expression.

Rostral migratory stream that extends from the lateral ventricle to the OB had high average expression for *Lgals9* along with its the positive expression in the SVZ (**Figure 3B**), GCL and the GL in the OB, where neuroblasts finally reach (**Figure 2B**). This highlights a role for *Lgals9* in the process of neuroblasts migration, probably as a guidance cue, which is further supported by its reported role in the cell–cell adhesion which may assist chain migration in RMS, as previously reported for *Lgals3* (Comte et al., 2011). The RMS was weakly positive for other galectins too. For *Lgals3* and *Lgals12*, there were few cells with below moderate expression in both SVZ and different layers of OB, and the RMS also had a similar kind of expression for them, revealing a possible involvement in tangential neuronal migration which has been suggested by a previous study on *Lgals3* (Comte et al., 2011). *Lgals3* has also been shown to promote neural cell adhesion (Pesheva et al., 1998) and this function might help in the process of rostral neuronal migration.

### The Thalamus (THA) and the Hypothalamus (HYP)

Thalamus relays sensory and motor signals to the cerebral cortex and is involved in regulation of sleep, alertness and consciousness. Hypothalamus links the nervous system to the endocrine system *via* the pituitary gland and is involved in regulation of body temperature, hunger, thirst, fatigue, sleep, circadian rhythm and the attachment behaviors.

In both thalamus (THA) and the hypothalamus (HYP), a high number of cells were positive for *Lgals9* and along with this density there was also an accompanying heterogeneity in the intensity levels (**Figures 3C,D**) with cells having low, below average and average levels of *Lgals9* transcript. Thalamic neurons positive for *Lgals8* were few and had low intensity expression. *Lgals12* had about 20% and *Lgals1* had around 10% cellular positivity in the thalamus but with below average intensity. More than 90% of cells positive for *Lgals3* and *Lgals7* also showed low expression levels. The number of *Lgals8* positive cells in the hypothalamus were less but the expressing cells had average or above average intensity levels. HYP was also positive for *Lgals12* but only few cells showed the expression. Galectin-3 and 4 too had low intensity level cells in the HYP. The lowest expression in HYP was for *Lgals2* with sparse number of cells being positive for this galectin. *Lgals1* showed less expression in HYP with a very few cells expressing in different intensity levels.

E2F1 and EP300 (Supplementary Figure S9, **Figure 5B**) which may regulate *Lgals9* had the same kind of expression pattern for almost all the sub regions in HYP. This was also the case with the thalamus. On the other hand, *Lgals3* had weaker but widespread expression intensity in the THA which could be regulated by FOXA2 and PPARG. *Lgals3* expression could also be regulated by a few other putative TFs like RUNX2, CTCF, ESRRB, KLF4, MYCN, STAT3 and CDX2 that had a sporadic expression in THA and might be enough to transcribe *Lgals3*. *Lgals1* and *Lgals2* showed scattered expression with weak intensity and putative TFs regulating these two galectins were among those with spread out and scarce expression. Also, both these galectins showed an overlapping expression pattern.

### The Amygdala

Amygdala has primary roles in processing of memory, decision making and emotional reaction. Amygdala is shown to be responsible for the acquisition and expression of conditioned fear through glutamatergic spiny projection neurons, GABAergic interneurons and the GABAergic medium spiny neurons (Tovote et al., 2015).

Levels of different galectins in the amygdala (**Figure 3E**) were similar to those present in the HYP (**Figure 3D**) with the highest levels for *Lgals9* and *Lgals8* followed by reduction in number of cells for *Lgals12*. *Lgals3* and *Lgals7* positive expression was found in nearly 70% of the cells of this region but cells had low levels of expression intensity. *Lgals1* and *Lgals4* showed the same kind of expression with very few cells having below average expression intensity only.

### The Cerebellum

The cerebellum is majorly involved in the motor control and has a unique laminar organization consisting of various types of neurons with granule cells being predominantly glutamatergic input neurons of the cerebellar circuitry whereas the Purkinje cells are GABAergic and act as major output neurons of the cerebellum.

Cerebellar granule cells (GCL) (**Figures 4A** and **5A**, Supplementary Figure S3, Supplementary Table S4) expressed *Lgals9* nearly exclusively with few cells having moderate level of expression in all the lobes and a few cells had above moderate expression intensity mainly in lobes I/II, IX and X. *Lgals3* expressed uniformly in the GCL of the cerebellum, however, with a very low expression. Similarly, *Lgals8* showed a very low expression in GCL but had a few cells with below moderate intensity levels. *Lgals4* and *Lgals12* showed transcript positivity only in the Purkinje cell layer (PCL), while *Lgals3* and *Lgals8* showed higher expression in PCL as compared to GCL which is quite easily distinguishable from the **Figure 4** that depicts a sharp boundary of cells. *Lgals1* showed very low expression levels but it was distributed in a good number of cells making it clear that it was expressed in the PCL. Molecular layer for all the galectins showed the lowest expression with an exception of *Lgals9* which had some cells with below average intensity expression. *Lgals3* and *Lgals8* showed the highest number of positive cells with low to moderate average expression in the deep cerebellar nuclei (DCN) of the cerebellum. This expression in DCN was closely followed by *Lgals1* and *Lgals9* which had high intensity expressing cells with some low intensity expressing cells too making their average expression slightly less. Galectins -2, 4, 7, and 12 had the lowest expression levels in DCN with only few positive cells.

The distinctive boundary of PCL in *Lgals8* and *Lgals3* highly correlated with the expression of MYC and ESRRB (Supplementary Figure S10, **Figure 5B**, Supplementary Table S4) which were predicted to regulate both these galectins. SOX2 which may regulate *Lgals8* had moderate expression in PCL and lower expression in GCL which made its presence in PCL more visible too. PPARG which regulated *Lgals3*, showed a clear expression in PCL.

A careful examination of *Lgals9* also revealed its expression in PCL which was otherwise not explicitly visible but *Lgals9* regulatory TFs positivity in this layer gave the first indication of the possibility of its presence in the PCL. E2F1, EP300, SETDB1 and SMARCA4, the putative TFs of *Lgals9* also showed expression in PCL. STAT3, predicted to regulate both *Lgals3* and *Lgals9* also showed a characteristic expression in PCL.

Moderate expression of *Lgals9* was observed in GCL which coincided with the expression pattern of E2F1, EP300 and MYC. Also SMARCA4, SETDB1, POU5F1 and CTCF positive cells in GCL showed some heterogeneity in the expression intensities.

### The Substantia Nigra

Substantia nigra is predominantly made up of dopaminergic neurons and is also a major site for motor co-ordination.

The entire SN region (Substantia nigra pars compacta, SNc; and Substantia nigra pars reticulata; SNr) (**Figures 4B** and **5A**) was moderately positive for *Lgals9* and its putative regulatory TFs, with the highest expression of E2F1, SMARCA4, SETDB1 and FOXA2 (Supplementary Figure S11, **Figure 5B**). Similarly, *Lgals8* had a moderate expression in the whole SN region but the cellular density was less compared to *Lgals9*. SOX2 and POU5F1 that putatively regulated *Lgals8* showed the same density but different expression intensities. Interestingly, the TF FOXA2, which in our TRANSFAC analysis was predicted to regulate galectins-2, -3, -7, -8, and 9 (Supplementary Table S2) has been previously documented to regulate multiple phases of midbrain dopaminergic neuron development. The mouse lacking a copy of FOXA2 shows abnormalities in the motor behavior (Ferri et al., 2007; Kittappa et al., 2007). Hence, the involvement of these galectins in motor behavior could be extrapolated to galectins 8 and 9 which showed moderate expression in motor control areas, although galectin-2, -3 and -7 positive neurons could also contribute to motor control functions even though only a few cells were identified to be positive in SNc.

### Validation of Regional Transcript Profile With Galectin Protein Expression in the Mouse Brain

The brain galectins transcriptome analysis was further substantiated with the evidences of near about matching patterns of major galectin protein expression (-1, -3, and -9) in different anatomical regions, however, due to the secretory nature of this protein, the regional pattern of spread was far wider, with signal detection in the extracellular matrix and on the cell surfaces (**Figures 6**–**8**). It is to be noted that cells that showed positive transcript can be much lower in protein levels than ‘the transcript low or transcript null’ neighboring cells due to high secretion of these proteins into the microenvironment for prospective autocrine and paracrine effects.

### Functional Annotation of Galectins

Upon identification of the heterogeneous expression of various galectin transcripts across the brain sub-structures, we resorted to the literature search (data-based) and GO analysis (informatics-based) to (i) validate our expression data and observations with at least some similar evidences in literature [Supplementary Table S3: *worksheet 1 and 2*] and (ii) to predict newer functions of prominent galectins *via* the extrapolation on the known functions of their putative TFs (Supplementary Tables S5 and S6).

In this context, a study has shown an increase in the expression of *Lgals1* in facial motor neurons (VII) after nerve injury (McGraw et al., 2004) which corroborates well with the evidence of the presence of *Lgals1* in region VII in our expression analysis. Another functional study for *Lgals1* suggests its role in proliferation of neural progenitors in the hippocampus (Kajitani et al., 2009), which was also evidenced by the presence of few positive cells in the SGZ layer of the DG in our own studies (**Figure 3**; Supplementary Figure S2). The low expression of *Lgals1* in hippocampus, as observed by us, was also supported by the study in which the authors had found that *Lgals1* was mainly expressed in the interneurons of the hippocampus that also expressed axonal marker beta tubulin-III (Kajitani et al., 2014).

In our survey of *Lgals1* functional annotations, the term ‘regulation of apoptotic process’ (*P* = 1.76E-3) was underpinned. Indeed, one of the study has suggested selective proapoptotic role of *Lgals1* in a subpopulation of GABAergic interneurons (Bischoff et al., 2012), hence providing evidence for correctness of our informatics based predictions. The informatics driven GO term ‘response to wounding’ (*P* = 1.16E-3), that was extrapolated to be the function of *Lgals1* based on the roles of its putative regulatory TFs, was supported by the evidence where *Lgals1* induced astrocyte differentiation and helped in preventing neuronal loss after injury (Sasaki et al., 2004; Ishibashi et al., 2007). Some of the TFs (Supplementary Table S2), such as CTCF (neural development), E2F1 (neurogenesis, forebrain development), PPARG (neuronal cell survival) and ESRRB/POU5F1 (stem cell maintenance, cell proliferation) were predicted to be the regulators of *Lgals1*, which further supports the validity of informatics based extrapolations as *Lgals1* is known to play a role in stem cell maintenance and neurogenesis (Cooper-Kuhn et al., 2002; Wada et al., 2006; Chin et al., 2009; Watson et al., 2014) (*please see* Supplementary Table S3: *worksheet 1 and 2*). Similarly, through the extrapolations on the known functions of *Lgals1* putative TFs like E2F1 (Wang et al., 2007), KLF4 (Su et al., 2014), MYCN (Wartiovaara et al., 2002) and PPARG (Fuenzalida et al., 2007), its role in neuroprotection was underpinned (Supplementary Table S3: *worksheet 1 and 2*).

A recently published study (Sirko et al., 2015) suggested a regulatory role for galectins 1 and 3 in proliferation and in imparting neural stem cell potential to a subset of reactive astrocytes, which supports *Lgals3* role in neurogenesis. This role of *Lgals3* was again corroborated with the putative TFs regulating its expression, which too had some role in neurogenesis in hippocampus (HIFIA, RUNX2), SVZ (SOX2, SUZ12, POU5F1), thalamus and the cerebellum (ESRRB, MYC) (Supplementary Table S3). Possible regulation of *Lgals3* by KLF4 in sub-ventricular zone and thalamus predicts its role in neuronal migration and its possible regulation by PPARG in thalamus and cerebellum shows an anti-apoptotic role (Supplementary Table S3). Besides, putative regulation of *Lgals3* by SMARCA4, STAT3 (Matsumoto et al., 2006; Muller et al., 2009) along with FOXA2 and E2F1 has already been discussed (Supplementary Tables S3 and S4). GO classification which was based on the known functions of individual galectin regulatory TFs (putative) supported the role of both *Lgals1* and *Lgals3* in stem cell proliferation and differentiation. However, *Lgals3* was also implicated/predicted to play a role in locomotion, response to light, eating behavior, eye/forebrain/hindbrain development, extracellular matrix metabolic pathways of collagen and elastin, iron and calcium homeostasis, regulation of vesicle mediated transport, Notch/cytokine/JAK-STAT/TGFB2 and smoothened signaling, viral infection associated processes and acute inflammatory response (Supplementary Tables S3 and S4).

*Lgals8* along with -2,-3,-7, and 9 were putatively regulated by TF FOXA2 which was associated with the GO term ‘stem cell differentiation’ and ‘regulation of neurogenesis’ (Supplementary Table S5), suggesting the reason for observed galectin-8, -2,-3, and 9 expression in the hippocampus. *Lgals8* was also possibly regulated by glial fate determination gene, SMARCA4 in the OB and cerebral cortex. SOX2, a neural stem cell maintenance TF could possibly regulate *Lgals8* in OB, hippocampus and cerebellum, again suggesting a role for galectin-8 in cell proliferation (Supplementary Table S3). Among other GO terms of particular interest in the context prediction of *Lgals8* functions were forebrain/hindbrain development, response to nerve growth factor and establishment/maintenance of apical-basal cell polarity (Supplementary Tables S5 and S6). Some of the important brain associated functions such as response to nutrient and forebrain/eye development were also predicted for *Lgals7* and *Lgals4* respectively. Regulation of lipid kinase activity, lipid storage, PPAR signaling and response to inflammation and regulation of sister chromatid cohesion as well as G1/G2 mitotic phase transition processes were also inferred for *Lgals4* (Supplementary Tables S5 and S6). Go classification also suggested that *Lgals2* may have important roles in protein targeting to the nucleus, stem cell biology and neuronal migration (Supplementary Tables S5 and S6).

Further, out of 16 TFs putatively regulating *Lgals9* (Supplementary Tables S2, S3, and S5), 13 were involved in ‘regulation of developmental process’ (*P* = 2.39E-10) and amongst these 13, seven were involved in ‘CNS development’ (*P* = 3.53E-7) along with some other TFs which had some role in making brain development possible. Of specific interest were the *Lgals9* associated GO terms: stem cell proliferation, maintenance and differentiation; regulation of regeneration, neurogenesis and gliogenesis; regulation of G1 to S phase cell cycle transition and asymmetric cell division; response to nutrient, fluid shear stress, wound healing, oxidative stress and hypoxia; development of hindbrain, hypothalamus, radial glial and dopaminergic neurons differentiation; cell-cell adhesion and communication; regulation of lipid metabolic process; regulation of cholesterol transport and protein import into the nucleus especially SMAD; positive regulation of mitochondrial membrane potential and chromatin remodeling; positive regulation of Notch, Protein kinase B, canonical WNT and JAK-STAT signaling. One of the most intriguing GO terms associated with *Lgals9* was the ‘response to the steroid hormone estrogen’, suggesting that this galectin may be involved in generation of sex specific changes in brain activity (Supplementary Tables S5 and S6).

In the OB, *Lgals9* may have role in memory formation (EP300), cell cycle regulation (SETDB1) and stem cell proliferation (POU5F1) based on functions of the putative regulatory TFs. In cerebral cortex and hippocampus, *Lgals9* may be involved in memory formation (EP300), where it may be additionally involved in stem cell proliferation (MYC, POU5F1) and differentiation (FOXA2). *Lgals9* may also regulate development of SVZ, hypothalamus and basal ganglia due to its putative regulation by the forebrain development associated TF- E2F1. Besides, it may promote neuronal proliferation (MYC, SETDB1, POU5F1) migration (KLF4) and differentiation of dopaminergic neurons (FOXA2). In the cerebellum, *Lgals9* may be required for glial cell fate (SMARCA4), astrocyte differentiation (STAT3) and various aspects of hindbrain development (SMARCA4) (Supplementary Table S3). So, from this, it can be said that *Lgals9* followed by *Lgals8, -1 and -3* may serve a major role in the mouse brain development, while other galectins may refine the brain architecture and functions rather more subtly.

### Galectin Family Gene Expression Analysis in Human

Similar to that in mouse, informatics driven approach identified a total of 174 TFs in the vicinity of different galectin genes that could serve as the possible regulators of galectins’ transcription (Supplementary Table S7). Subsequently, all 174 TFs were taken together along with the galectins for module detection and functional network construction. This approach, combined with literature search on the functions of TFs that putatively regulated galectins, bestowed us with a unique opportunity to predict the functions of galectins in the human brain (Supplementary Tables S9, S16, and S17).

### Network Construction and Module Detection

**FIGURE 9:**
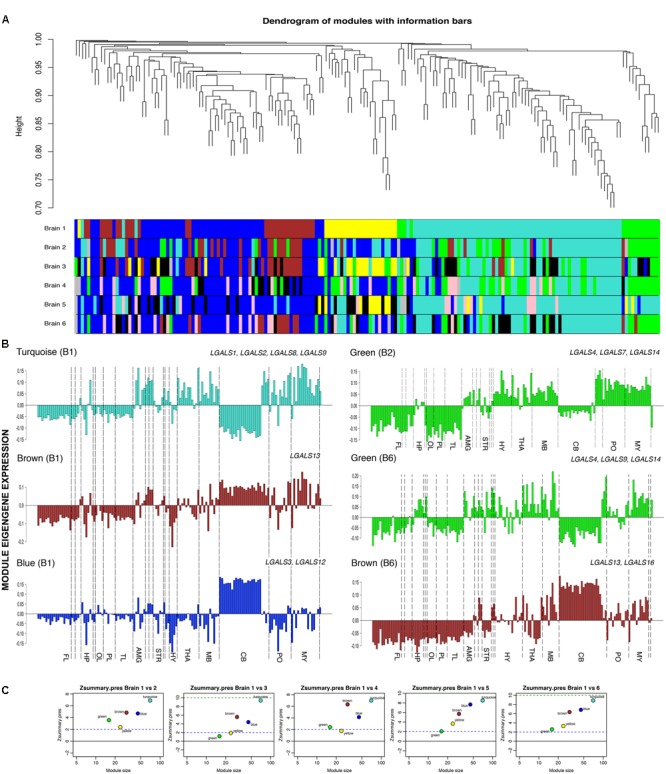
**Weighted co-expression networks and modules. (A)** Top: Dendrogram from gene co-expression network analysis of galectins and their regulators. Bottom: Modules of co-expressed genes were assigned a color and all modules from each human brain are shown. **(B)** Module eigengene expressions for different modules in different brains are shown, FL, frontal lobe; HP, hippocampus; OL, occipital lobe; PL, parietal lobe; AMG, amygdala; STR, striatum; HY, hypothalamus; THA, thalamus; MB, midbrain; CB, cerebellum; PO, pons; MY, medulla. Selected galectins in each module are shown. **(C)** Preservation measure Z_summary_ between brain 1(B1) *vs.* the other 5 donor brains.

To identify the unique and biologically important expression pattern of the transcriptome of galectins in human brain, we analyzed a high-quality microarray data set from the Allen Human Brain Atlas (AHBA) using WGCNA (Zhang and Horvath, 2005; Langfelder and Horvath, 2008) (Supplementary Tables S10 and S11). This method helped in discovering the co-expression relationship between genes and enabled their grouping into modules based on Pearson correlation. These modules have been used to gain insights into the functionally related co-expressed genes. This co-expression weighted network, based on TOM was created by taking the Pearson Correlation Matrix and transforming it into connection strength matrix, which is the degree of the shared connection between the two genes obtained by increasing the correlations by raising to the threshold power (Yip and Horvath, 2007). TOM was calculated for each gene pair which considers both correlation and their shared relationship. In a weighted network, each gene was assigned to a module and through these tools; we identified 5 distinct co-expression modules with 16–70 genes per module as shown in the dendrogram of genes clustered according to the closeness of the expression pattern (**Figure 9A**).

### Module Membership and Preservation

To represent each module within a network, it was summarized by module eigengene which is the first principal component that presents expression profile for that module and helps in explaining the variability of all genes in a module (**Figure 9B**).

Each gene was also provided with another measure called module membership (kME), which is the absolute value of correlation between the expression level of each gene in the network and each module eigengene, along with their *P*-values (Supplementary Table S12). This measure informed us about the level to which a gene belonged to a module on the scale of 0–1. Genes with high module membership in their respective modules helped us in elucidating the function of a member gene based on the annotation of the module. To know the hub genes of a network, intramodular connectivity was measured (Supplementary Table S13), which informed us on how frequently a node interacts with other nodes and genes with the highest intramodular connectivity were called as the hub genes. The hub genes helped in identifying module’s functions or predicting the role of an unknown gene from the module properties.

Z_summary_ statistics, a permutation test that creates an opportunity to test the preservation between different brains allowed us to assess the preservation of modules in the other five donor brains with respect to the first donor and the modules were assigned colors according to the modules from donor brain 1 (B1) for preservation comparisons (Supplementary Tables S14 and S15). Our study showed that genes of the modules with the same color were highly preserved and stayed together under the dendrogram (**Figure 9A**). High preservation score was detected in 3 out of 5 modules across different donor brains (**Figure 9C**). The gene modules (blue, brown, and turquoise) identified in donor brain 1 were well conserved in other donor brains too, at the level of regional gene expression, i.e., in structures and sub-structures, as measured by a module preservation index (Supplementary Table S15).

### Module-Wise Galectin Gene Expression and Annotation

Genes belonging to a certain module helped in identifying (1) which all galectins showed similar expression pattern across the brain; (2) which galectins were enriched in only one part of the brain and (3) which subregions or major parts of brain showed similar galectin profiles. Besides this, the variability of galectin expression could be also analyzed at the global and local levels with this approach. The variability at the local level could elucidate more functional roles of genes depending on where they were expressed, while the variability at the global level could illuminate us toward the heterogeneity and distinction between each brain. The relationship of module eigengene expression profile with individual structures was based on the differential or relative expression of each galectin and TF across its own absolute expression.

Out of seven modules indentified, turquoise module (consisting of *LGALS1*,-*2*,-*8*, and -*9*) was most preserved across donor brains. The other modules which showed presence of galectins were the brown module (*LGALS13*, -*16*), blue (*LGALS3*, -*12*) and the green module (*LGALS4,-7*, -*14*) (Supplementary Table S14, *please refer to all worksheets*).

Out of the five modules that we found in donor brain1 (B1, reference donor), the turquoise module consisted of 70 genes including four galectins (1, 2, 8, 9) (Supplementary Table S14). This module was positively correlated with globus pallidus (GP), thalamus (THA), midbrain (MB), cerebellar (CB) nuclei, Pons (PO) and medulla (MY), while negatively correlated with cortex (CTX), hippocampus (HP) and the cerebellar cortex [CB CTX] (**Figure 9B**). Significant heterogeneity was found in the hippocampus (HP) for turquoise modules in B1 (brain1) and B3 (brain 3), where CA4 showed a positive relationship. Similarly, amygdala (AMY) showed variability in its individual substructures for this module.

*LGALS1* was present in turquoise modules in all the six brains (Supplementary Table S14) and had a high module membership value (kME) in all of them, i.e., >0.75 (Supplementary Table S12). Along with *LGALS1*, putative TFs which had high kME values, showed similar expression patterns in those regions where the module was positively correlated. With these observations, it can be said that knowledge on genes in turquoise module for which some biological process, pathway or function is known, can assist in validating the established functions as well as enable the prediction of novel roles for *LGALS1* from their close relationship. A similar approach could be further used for all other galectins that showed up in the conserved modules.

**FIGURE 10:**
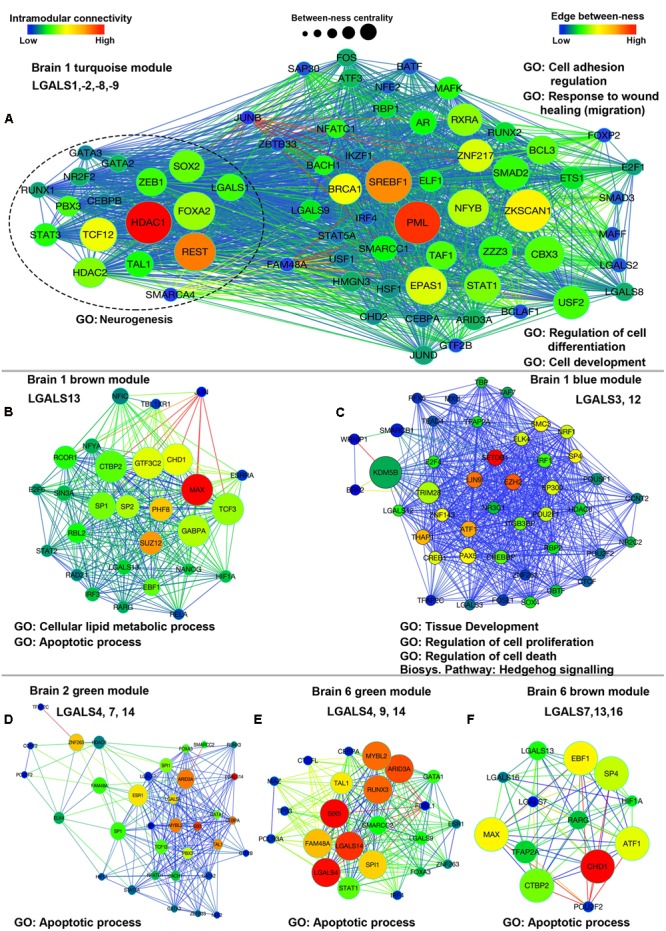
**Network plots showing co-expression interaction of module for which module eigengene expression is shown in **Figure 9B**. (A)** Brain 1 (B1) turquoise module, **(B)** Brain 1 brown module, **(C)** Brain 1 blue module, **(D)** Brain 2 green module, **(E)** Brain 6 green module, **(F)** Brain 6 brown module. Color of each node represents measure of intramodular connectivity; node size denotes the between-ness centrality and color of edges show the edge between-ness, which is the weight of each edge. Major GO classification terms are mentioned against each module. Conserved galectins in each module are also indicated.

Apart from the GO terms for the regulation of gene expression (Supplementary Tables S16 and S17), the turquoise module was predicted to be enriched in genes involved in ‘immune system development’ (*P* = 8.304E-18), ‘cell fate commitment’ (*P* = 6.851E-13), regulation of cell–cell adhesion (*P* = 6.051E-03) and ‘cell proliferation’ (*P* = 8.856E-13). Among all the informatics based biological processes identified for turquoise module, each process showed at least one or more galectins associated with them, for example, *LGALS1* was associated with GO terms ‘regulation of cell differentiation’ (*P* = 3.298E-18), ‘cell development’ (*P* = 3.243E-9), ‘regulation of nervous system development’ (*P* = 3.882e-6) and ‘neurogenesis’ (*P* = 1.595e-5. TCF12, REST and HDAC1 were the 3 TFs that were present among the top 10 hubs of turquoise module and were predicted to be involved in regulation of neurogenesis along with *LGALS1* (**Figure 10A**, Supplementary Tables S16 and S17). Similarly, potential functions for other galectins could be obtained by the logic of guilt-by-association for other modules too (**Figures 10B–F**, Supplementary Tables S16 and S17). Apart from employing GO analysis to predict genes involved in the process of neurogenesis, we went through the literature and found 6 more TFs (E2F1, FOXP2, HSF1, IKZF1, SMAD3, and TAF1) that could be associated with neurogenesis as mentioned in Supplementary Table S9. For the GO term on the ‘regulation nervous system development’, 8 out of 11 TFs were putatively involved in the regulation of *LGALS1*.

Further, in turquoise module, *LGALS8* had over-expression in hippocampal region and *LGALS9* also showed some positivity in CA4 region. Upon careful consideration of the GO classification and the positive expression of galectins-8 and 9 in the subregions of hippocampus, it can be proposed that these two galectins may be also involved in hippocampal neurogenesis. Interestingly, GO classifications for putative TFs regulating *LGALS8* and *LGALS9* also showed that these galectins could be involved in neuronal survival or neural cell fate determination.

*LGALS2* which mainly belonged to turquoise module according to WGCNA analysis, showed low kME values in all the brains (<0.5), which suggests that this galectin might not belong to any particular module or may not have high correlation with the module eigengene expression, so it needed curation in each brain individually, by looking at its expression profile and not by looking at the module eigengene’s expression. Further, blue (B1) and brown (B1) modules showed positive correlation with GP and CB cortex. The same modules showed positive correlation in the DG of HP in atleast 2 brains (B1 and B4), while in rest of the hippocampal subregions this correlation was identified to be low (Supplementary Table S12). In the brown (B1) module, HYP and THA showed variation among all the subregions (Supplementary Table S12). Similarly, many genes were expressed in different structures with variable expression but it was their relative expression level which helped in deciding if the gene was really expressed in that particular area and whether the expression was similar across all the regions.

The common predicted theme between blue (B1) and brown (B1) modules was ‘cell death,’ but the genes in blue module were also enriched in ‘cell proliferation’ (*P* = 1.002E-4) which distinguished this module from brown module even though they had similar expression pattern for different regions (**Figures 10A,B**, Supplementary Tables S16 and S17). Also blue module (B1) corresponded to GO terms ‘stem cell development’ (*P* = 2.007E-7), ‘cell cycle’ (*P* = 5.796E-7), ‘developmental induction’ (*P* = 1.251E-4) and like the turquoise module it was predicted to be involved in ‘cell fate commitment’ (*P* = 4.163E-4). In the context of galectins, *LGALS3* was the major galectin present in this module and had putative roles in ‘tissue development’ (*P* = 5.77E-5), ‘regulation of cell proliferation’ (*P* = 2.036E-3) and ‘regulation of cell death’ (*P* = 7.914E-3).

As we had already searched the literature for more biological roles in the context of neuronal processes for galectins’ putative regulatory human TFs (Supplementary Table S9) apart from those established with the help of GO enrichment analysis, we could gain further insights into the possible functions of galectins from the modules with which these TFs were associated. Through this prediction approach, we found that the turquoise module showed association with all major brain processes such as neural cell proliferation, differentiation, neurite extension, migration, survival and synaptic transmission. The blue, brown, and green modules showed protection against viruses as a common function. While the activation of neural gene process was uniquely associated with turquoise module; neural stem cell differentiation and lateral ventricle differentiation were exclusively associated with blue and brown modules respectively. Both green and the brown modules were also more associated with processes pertaining to differentiated neurons such as synaptic plasticity, synaptic transmission and other synaptic functions (Supplementary Tables S18–S21).

The other possible roles played by the members of blue module (*LGALS3, -12*) included neural migration, neuronal survival/anti-apoptosis, CNS development and neural differentiation (**Figure 10C**, Supplementary Tables S16 and S17).

The green module (*LGALS4, -7, -14*) was not preserved with high confidence in all the brains (Supplementary Table S15), but *LGALS4* and *LGALS14* were well maintained in most of them (**Figure 9B**, Supplementary Table S14) and were amongst the hubs in green module network of some of the brains (Supplementary Tables S12 and S13, **Figure 10B**). Hence, functions associated with the members of green module could highlight some viable role for both *LGALS4* and *LGALS14*. In the donor brain 6 (B6), *LGALS9* had high membership in green module of some donor brains (**Figure 9B**, Supplementary Table S14) which again demonstrates the heterogeneity between donor brains and that the age, diet, ethnicity, gender etc. could majorly regulate galectins’ expression (Rhodes et al., 2013).

Another important observation that we made was that even though the total number of genes belonging to a module differed in each brain (Supplementary Table S14), some of the intramodular hub genes (mainly top 5 in each module) were remarkably reproducible across the human brains (Supplementary Tables S12 and S13). For example, RE-1 silencing transcription factor (REST) was consistently identified as hub in Brains 1, 2, 3, and 5 in the turquoise module and this TF has been shown to have a role in neurogenesis (Gao et al., 2011) and neuronal differentiation (Su et al., 2004). Similarly, EZH2, associated with functions in neural migration, proliferation and neurogenesis and SETDB1 which is connected with roles in neuronal survival and CNS development were identified to be hubs in brains 1, 2, 3 and 4 in the blue module (Supplementary Tables S12, S16, and S17). This information highlights that in general, the important brain functions and processes were preserved as hubs in all the donor brains, resurrecting the fact that human brain are fundamentally similar and the divergences in gene expression profiles were rather more subtle.

Emergent knowledge on the ‘preservation of hub genes’ presented one more advantage to this analysis which surprisingly revealed that the TFs that emerged as hubs were also the putative regulators of galectins in each module (**Figure 10**, Supplementary S14). For example, out of 10 TF hubs in turquoise module, 7 hubs regulated *LGALS1*, 9 hubs regulated *LGALS2*, 8 hubs regulated *LGALS8* and 9 hubs regulated *LGALS9*. Similarly in the blue module, 9 hubs regulated *LGALS3* and in the brown module 7 hubs regulated *LGALS13*. This analysis thus helped us in deciphering the deeper insights into the overall uniqueness of brain spatial transcriptomics heterogeneities as well as commonalities from the results obtained on spatial organization of galectins and their predicted regulators.

### Comparative Gene Expression Analysis between Mouse and Human

Upon comparing the data driven expression of galectins in mouse and humans at a global level, *Lgals9* showed both the widespread and the highest expression in all the brain regions in mouse, while in humans, *LGALS1* had almost absolute ubiquitous expression, but relative to individual structures, it was under-expressed (Supplementary Table S22). At a local level, that is at the scale of individual structures and substructures some regions showed similar profiles of galectins and other structures showed an opposite trend. This was much anticipated as mouse is an evolutionary different species and has predominantly different physiology than human. Species specific spatial distribution of molecular signatures between mouse and human brain has been previously reported (Zeng et al., 2012) and this fact could be the major cause of the observed differences in regional identity, the levels and distribution of galectins in these species.

Strikingly, in the human hippocampus, considering only the absolute expression values, DG region showed the highest expression of *LGALS1* amongst all galectins but overall the expression of *LGALS8* was the highest (Supplementary Table S22). On contrary, in mouse hippocampus, *Lgals9* showed the highest expression in DG whereas its expression was the third lowest in human in the same region (Supplementary Table S4, **Figures 3A** and **4A**).

In humans, the galectins’ expression in amygdala was divided into substructures, where individual structure has expression close to its mean, *LGALS1* showed the highest absolute expression closely followed by *LGALS8* while *LGALS9* and *LGALS12* were the lowest (Supplementary Table S22). However, in mouse amygdala, *Lgals9* expression was the highest followed by *Lgals8*, which points to the consistency of expression of galectin-8 which was highly preserved in both the species (Supplementary Table S4, **Figures 3E** and **5A**). Similarly, *LGALS3* showed high expression in human amygdala but it was low for mouse. However, some conservation in galectin-3 expression profile was observed for hypothalamus and thalamus in both the species.

In human basal ganglia, consisting of globus pallidus (GP) and striatum (Str), galectins -1, 3, and 8 showed high expression, on contrary, in mouse, the expression of these galectins was the lowest in the same region (Supplementary Table S4, **Figures 2C** and **5A**). While *Lgals9* showed highest expression in mouse, it showed an opposite trend in human, where its expression was amongst the lowest. So, basal ganglia also served as a case where the profile between mouse and human was quite opposite (Supplementary Table S4; **Figure 5A**). However, it is to be noted that the contribution of galectin-9 to brain structure and functions cannot be underestimated in humans as it has been documented to control significant cellular and pathological signaling (John and Mishra, 2016).

One of the important brain structures which develops mostly postnatally in mouse and *in utero* in human is the cerebellum (Haldipur et al., 2012). In this part of the human brain, *LGALS2* was least expressed (Supplementary Table S22), which was quite consistent with its expression in mouse (Supplementary Table S4; **Figure 5A**). But the other galectins did not follow the same trend. In the mouse cerebella, *Lgals9* had the highest expression across galectins, while in humans it was quite low as compared to the other galectins (Supplementary Tables S4 and S22, **Figure 5A**).

Although for mouse, the expression analysis for cortex was done layer-wise and in humans it was according to the lobes and its different sub-structures, it was observed that in mouse, *Lgals9* had the highest average expression across the layers among all the galectins analyzed, while in humans too, this galectin had maintained the same kind of profile for the cortex, hence the two different techniques employed to identify galectin-TF transcript expression (ISH for mouse *vs.* microarray for human) well corroborated with each other.

Substantia nigra, consisting of dopaminergic neurons showed high density and moderate expression of *Lgals9* in mouse (Supplementary Table S4; **Figure 5A**), but in human, the expression was low as compared to galectins 1, 3 and 8, where *LGALS1* was the highest (Supplementary Table S22: *please see all worksheets*).

Between mouse and human, 18 common TFs were predicted to be involved in regulating the expression of different galectins (Supplementary Table S8). Amongst those, 4 did not pass the image quality test by ABA and hence the remaining 14 were analyzed by using the ISH images in mouse. Among these 14 common TFs, SMARCA4 and SOX2 showed the highest absolute expression values in human across all the regions (Supplementary Table S22: *please see all worksheets*). In mouse, these two TFs came together along with *Lgals8* when clustered hierarchically (Supplementary Table S4; **Figure 5B**), hence pointing to the co-expression of these TFs with *Lgals8* in both the species. The major role for these TFs has been reported in neurogenesis, which again validated their co-expression probably for the co-ordination of a common function. But in humans, only SMARCA4 putatively regulated galectin-8 depicting a difference in the regulation in different species.

EZH2 which is involved in neurogenesis, neuronal migration and memory formation, in both humans and mouse, had very low expression in mouse hippocampus (Supplementary Table S4; **Figure 5B**) but had few cells with low level average expression in the SGZ of DG in mouse. Similarly, human brains showed highest absolute expression of this TF among all sub-regions of the hippocampus (Supplementary Table S22). This TF also putatively regulated galectins-8 and 9 in both the species (Supplementary Tables S4 and S22, **Figure 5B**: *please see all worksheets*).

E2F1 has been reported for a role in neural development and neuronal survival and our analysis showed that it regulated all the galectins (1, 2, 3, 4, 7, 8, and 9) in mouse, with the highest expression among all the galectins and putative regulatory TFs. On contrary, its expression was among the lowest in humans, although it theoretically regulated the same galectins there too except for galectin-7 (Supplementary Tables S4 and S22, **Figure 5B**: *please see all worksheets*). This differences in levels of expression in both species suggest that either there may be different complex of TFs that were involved with this TF for its regulatory function or that the low levels of expression in human was sufficient for the proper regulation of its target genes. Similarly, there were several more TFs that were predicted to be common between the two species, which may have different regulatory roles and expression (Supplementary Table S8).

## Discussion and Conclusion

The major goals of the study were to describe (1) the neuroanatomical organization of galectins in brain substructures of mouse and human species [data driven] (2) predict probable functions of galectins through the known functions of co-expressed galectins’ putative regulatory TFs [informatics driven] and (3) to compare both intra and inter-species specific differences as well as the extent of conservation in galectins’ expression across brain structures and sub-structures [data and informatics driven inferences].

Here, to the best of our knowledge, it is for the first time that the brain-centered gene expression profile of galectins has been systematically described *via* the biocuration of raw ISH images of adult mouse from the ABA and the normalized microarray data for six human donor brains from the AHBA. Further, through a novel strategy of extrapolation of the regulatory TFs functions, galectins probable roles in the generation of brain heterogeneity are deduced and functional networks are generated, which now lays a strong ground for further detailed experiments.

Allen Brain Atlas driven neuro-anatomical/neuroinformatics studies have been particularly successful in accelerating the understanding of brain across species and has been vital in many innovations related to brain drug therapeutics^5^ (Allen Brain Atlas: 5 years and Beyond; Jones et al., 2009; Sunkin et al., 2013). Since the semi-quantitative approach for gene expression has been followed for all genes with ‘one size fits all’ high throughput approach, ABA, in recent years, has been robustly curated for further ‘context dependent analysis’ by independent investigators (Jones et al., 2009). However, the ABA has clearly discussed the technical reproducibility of ISH data over several sections from the same mouse brain^6^ (follow ‘Documentation’ Tab- *In Situ* Hybridization and Supplemental Data-ISH Platform Validation). They have also run appropriate positive and negative controls for the ISH data^6^ (follow ‘Documentation’ Tab-Supplemental Data-ISH Platform Controls). The data is generated on inbred mouse strain and excellent reproducibility across group of animals has been verified by re-examining the ISH patterns of about 1000 genes from the big dataset^6^ (follow ‘Documentation’ Tab-Supplemental Data-Comparison of the Top 1000 Genes) that confirms the ABA is a highly reliable, non-profit resource to neuroscientists for proposing challenging hypothesis for innovative and frontier research in brain sciences. Further, Allen Brain Atlas data has been verified to be highly accurate and consistent with the peer-reviewed literature and other independent high throughput brain gene expression/radioactivity based ISH databases such as BGEM and those generated by Prof. Fred Gage team (Lein et al., 2004, 2007)^6^ [follow ‘Documentation’ Tab-Supplemental Data-Cross Platform Validation]. The reproducibility and reliability of data is thoroughly discussed and demonstrated in supplementary section of the following publication: http://www.nature.com/nature/journal/v445/n7124/suppinfo/nature05453.html; http://www.nature.com/nature/journal/v445/n7124/pdf/nature05453.pdf) and is also demonstrated by others^5^ (Allen Brain Atlas: 5 years and Beyond; Jones et al., 2009). Our own work is similar to previous report that biocurated the ABA ISH data and found good concordance with the protein expression (Zhang et al., 2008). Furthermore, the normal human brain gene expression microarray data is available at the ABHA platform for minimum six donors for a reliable intra and interspecies cross comparisons, besides, the comparisons within the brain regions of each donor is made possible. Hence, we resorted to ABA and ABHA as a reliable platform to begin systematizing the ‘science of galectins’ in the perspective of brain structural organization and regulation.

Galectins have been deeply studied and elucidated in the functioning of immune system and with the discovery of immune system in brain (Louveau et al., 2015), it becomes all the more necessary and timely to understand the expression and roles of galectins in CNS. For example, immune system molecule IFNγ that has been recently shown to be involved in generating neuronal connectivities is crucially regulated by galectins (Blouin et al., 2016; Filiano et al., 2016; John and Mishra, 2016). Gal-3 aberrations have been identified to be hub in autism associated disorders, again pointing to the role in brain wiring and generation of social behavior (Voineagu et al., 2011). Besides, given the secretory nature of galectins, the detailed CNS expression, and regional enrichment and spread as morphogens needs to be timely elucidated to understand the role of this family of proteins in regulation of CNS architecture and functional circuits for cognitive performance and behaviors.

Our analysis revealed many intricacies in the expression profiles of galectins and their regulatory TFs in different regions of brain which has led us to uncover (1) informatics driven potential functions for individual galectins in brain, (2) reflect on the species specific signatures and (3) brain mRNA transcriptome regional and cellular heterogeneities *via* curation of ABA data.

This approach to understand galectins’ regional expression and functions *via* their regulatory TFs was particularly useful because the ‘cellular identity and function’ for defining the phenotype and genotype of diverse cells from multiple different regions has been observed to be the end product of the precise regulation of gene expression in each cell type. This control of gene expression is maintained by cell- or tissue-specific TFs (Müller et al., 1988; Vaquerizas et al., 2009) and their misregulation can lead to a variety of brain defects and neurodegenerative disorders such as Parkinson’s disease, Alzheimer’s disease and Amylotrophic Lateral Sclerosis (ALS) (Miller et al., 2006; Cooper-Knock et al., 2012; Ziats et al., 2015).

In fact, the regional heterogeneity in expression of galectin transcripts in brain could be a net outcome of distinct cellular transcriptional complex which consists of multiple TFs that outline the identity and function of cells in a region by regulating differential gene expression. This transcriptional complex consists of (1) facilitators which are ‘expressed in all the cells of a region’ and regulate multitude of genes and (2) specifiers which ‘distinguishes these cells into different cell types’ to give a context to these cells by regulating only specific genes (Vaquerizas et al., 2009). Our analysis in mouse revealed that four TFs, CTCF, E2F1, ESRRB and KLF4 could act as facilitators in the regulation of galectins as they were found to be putatively regulating all of them amidst the specifiers.

Further, the regional heterogeneity of the transcript is a direct function of the availability of transcription binding sites (TFBS) in the cell type. Chromatin immunoprecipitation with sequencing (ChIP-seq) (Johnson et al., 2007) has paved the way for identifying transcription factor binding sites (TFBS) in target genes that helps in elucidating the regulatory networks of a gene and even a whole family of genes. The putative TFBS obtained for galectins were located both far and near to their promoter sites and many of these sites are the possible locations where enhancers may bind and regulate galectins expression.

In ABA data curation, we find that several galectins were expressed in the same brain regions at the same time, though in a non-overlapping pattern in some neuronal populations and also with differing intensities. Hence, the differences in the regional expression of galectins and their predicted regulators in different brain structures may be explained to be occurring as follows: First, there could be a differential binding of TFs at the promoter sites. Second, there could be differences in cell density, in cell type, their morphology and the circuits they form with the other cell types in the same structure or in the different parts of the brain. Third, there could be differences in the activity of the cell type studied in different regions, that is, whether it is excitatory or inhibitory. Fourth, this could be due to the differences in the positioning of the brain regions (lateral, para-sagittal or mid-sagittal, for example). Finally, each cell in a region could be regulated separately or there could be differences in the regulation of different cell types in a region (Kasukawa et al., 2011) as was the case for *Lgals1* in the L5 of cortex, where only few cells expressed this galectin but strongly. Therefore, this diversity and distribution of different cell types in different structures of adult brain may underpin the regional complexity in its transcriptome too.

There could also be other extrinsic and intrinsic factors that could govern galectins’ expression heterogeneity and preservation. For instance, we identified a set of local neural circuits where the same galectin is expressed across the circuit and establishes the positive function of those circuits. For example in mouse brain, *Lgals3* and *Lgals8* were positively expressed in the cerebellar PCL and these cells provided output from the cerebellar cortex to the DCN, which too were positive for both of them. The heterogeneity of galectins in the Ammon’s horn of the hippocampus was possible due to the differences in the morphology of cells in each CA region. Similarly, the unique expression pattern was obtained for cerebellum in all human brain modules, which was quite distinctive in its module eigengene expression from the rest of the brain (Lein et al., 2007; Kang et al., 2011; Liscovitch and Chechik, 2013) probably to due to its development taking place after the birth (to some extent), unlike the rest of the brain development.

Contrary to data driven expression analysis, the prediction of functions for galectins was majorly possible by studying the co-expression of galectins and putative regulatory TFs between different regions and co-relating this information with the cellular identities of those regions. In human micoarray-based transcriptome analysis, the enrichment of genes in specific modules could also reveal unique gene signatures and functions for the un-annotated genes. Similarly, identification of different modules with different numbers of co-expressed genes, enabled prediction of the function of galectins in those modules with the aid of already known functions of the other members of that module by the principle of ‘guilt by association’ (Oldham et al., 2008). This possibility of shared function for the genes involved in each module based on the co-expression analysis from the microarray data has been described in earlier studies too (Hughes et al., 2000; Segal et al., 2003). In ABA data, we have indeed seen that for the co-expressed genes, expression profile of TFs at least matches partly with that of its targets, as was the case for *Lgals9* and *Lgals8* in mouse cortex.

Another point that must be highlighted here is that the number of possible TFs regulating galectins in humans ranged from 72 for *LGALS7* to 152 for *LGALS9*. There were large number of TFs that were common across all galectins. It is only by treating each galectin with its own regulators in WGCNA analysis, that we could generate modules where many of the regulators were separated on the basis of co-expression. A combination of galectins and their putative regulatory TFs’ transcript expression hence unveiled the prospective functions of galectins, because genes that cluster together are observed to share similar roles.

Similar to mouse brain, both measures of preservation and heterogeneity were observed for human brains, where the general framework for the brain development comprising of anatomically similar structures and resident neural circuitry established preservation. On the other hand, heterogeneity was possible due to differences in expression pattern, differences in the number of structures studied for each brain, age, sex, ethnicity and even diet of the brain donors. Preservation helped in identifying the common functions of genes in all donor brains while heterogeneity revealed another possibility of identifying a distinct role in different brains.

Hence, by systematically analyzing the gene expression in both mouse and human and comparing it in the common structures from both the organisms, we were provided with an opportunity to cautiously but promisingly consider mouse as a model organism for galectin associated brain diseases based on their transcriptome profiling in the two species which is akin to studies that have already established the conservation of gene expression between mouse and human (Strand et al., 2007).

Gene ontology analysis of different modules in human brains and possible regulators of galectins analyzed individually in both mouse and human, predicted enrichment in neurogenesis, neural cell migration, gliogenesis, response to wound healing, CNS development, cell fate commitment, cell proliferation, cell differentiation, cell cycle and regulation of cell death. With further identification of functions for different TFs in brain through literature search, we could predict more functions for galectins such as synaptic plasticity and maturation, neuronal survival, neural differentiation, neurite extension, protection against viral infection, response to inflammation etc., (Supplementary Tables S18–S21). Therefore, the method of analysis presented here display several opportunities. This method enabled (1) prediction of novel functions for galectins that has generated ‘leads for further experimental work, (2) it highlighted common and species specific galectin expression regulatory networks and (3) it identified brain microstructured-fine tuned heterogeneity and as the GO data is progressively updated, it can enable further more predictions of galectin functions in brain. Overall, this study sufficiently highlights that galectins heterogeneous transcriptome diversifies molecular and functional anatomy in both mouse and human brain. Hence, in conclusion, galectins can be considered as novel and emerging molecular signatures of brain heterogeneity. Ongoing studies in our lab on galectin-TFs structure-function relationship in mammalian brain will lend insights into the patho-physiological relevance of galectins expression and regulation and may further enable efficient maneuvering of these molecules for both therapeutics and regenerative purposes. Our recently acquired data makes it clear that the expression mask generated by our methodology corroborates well with galectin protein expression profile *vs.* the expression mask available at the Allen Brain Atlas (*please see* **Figures 6**–**8**; Supplementary Figure S15, detailed study on galectins developmental-adult protein expression will be communicated soon).

Overall, the major highlights of this work suggest that (i) galectins have a highly heterogeneous transcript expression within and across mouse and human brain anatomical locations (ii) galectins are predicted crucial targets of brain enriched TFs (iii) galectin-1, -3, -8, and -9 may regulate several neuronal processes, while galectins-2,4,6,7 and 12 may regulate more specialized and localized functions (iv) galectins-8 is most conserved across mouse and human brain (v) and finally, due to diverse regional distribution within and across species, galectins-the well known family of immune system proteins may be considered as novel signatures of brain heterogeneity and functions with several similarities to other recently reported HLA family of immune system proteins in the mammalian brain (Boulanger, 2009; Darmanis et al., 2015).

## Ethics Approval and Consent to Participate

The immunohistochemistry data was generated on C57/Bl6J mice strictly according to the protocols approved by the Institutional Animal Ethics Committee (certificate number IAEC/163/RM/2012) of Rajiv Gandhi Center for Biotechnology (RGCB).

## Availability of Data and Material

The datasets generated during and/or analyzed during the current study are available in the Allen Brain Mouse Atlas online database (ABA), web link- http://mouse.brain-map.org/ and TRANSFAC software (licensed software). The Allen Brain Human Atlas (AHBA) Microarray data is available at the following link: http://human.brain-map.org/static/download. However, the datasets supporting the conclusions of this article are included within the article and its additional files.

## Author Contributions

RM designed and performed research; SJ performed immunohistochemistry and generated **Figures 1** and **6**–**8**; Supplementary Figure S15. RM wrote the manuscript. Both the authors have read and approved the final manuscript.

## Conflict of Interest Statement

The authors declare that the research was conducted in the absence of any commercial or financial relationships that could be construed as a potential conflict of interest.

## Footnotes

1 http://www.alleninstitute.org/legal/citation-policy/

2 http://www.alleninstitute.org/legal/terms-use/

3 http://mouse.brain-map.org/

4 http://atlas.brain-map.org/atlas?atlas=2#atlas=2&structure=10703&resolution=15.79&x=7488.142596550707&y=3807.8964917164926&zoom=3&plate=100883770

5 http://www.alleninstitute.org/what-we-do/brain-science/research/scientific-publications/

6 http://mouse.brain-map.org/

## Abbreviations

ABA: Allen Brain Atlas
ABHA: Allen Brain Human Atlas
BGEM: brain gene expression map
CNS: central nervous system
ISH: *in situ* hybridization
symbol ‘*lgals*’: for mouse galectins
symbol ‘*LGALS*’: for human galectins
TF: transcription factor
TOM: topological overlap measure
TRANSFAC: *TRANS*cription FACtor Database
WGCNA: weighted gene co-expression network analysis.
