# Supplementary material for: mRNA Transcriptomics of Galectins Unveils Heterogeneous Organization in Mouse and Human Brain
Source: Front Mol Neurosci. 2016 Dec 16;9:139. doi: 10.3389/fnmol.2016.00139 (PMC5159438; doi:10.3389/fnmol.2016.00139)
Supplement: Supplementary file 1 [file Data_Sheet_1.PDF]

SUPPLEMENTARY FILES:

Supplementary Methods & Legends for Supplementary Figures and Tables

mRNA Transcriptomics of Galectins Unveils Heterogeneous Organization in Mouse and Human Brain

Sebastian John[1] and Rashmi Mishra[1]

1. From the Department of Neurobiology and Genetics, Rajiv Gandhi Centre for Biotechnology, Thycaud PO, Poojappura, Thiruvananthapuram, India-695014

Supplementary figures: 16

Supplementary tables: 22

METHODS

Prediction of potential transcription factors regulating galectins in mouse and human

An integrative approach was used for identifying the possible transcription factors regulating the gene expression of galectins in brain using TRANSFAC software. It contained information compiled from ENCODE project along with their own experimentally verified *in vivo* ChIP fragments using ChIP-chip/-seq data from different cell lines and tissues. These fragments were located near the vicinity of a particular gene and helped in predicting the transcription factor (TF) binding sites in an unbiased manner. The identified TFs were further examined for the possible and established roles in the neuronal processes, for example, function in neuronal survival, neurogenesis, brain development, migration, synaptic functions and maturation. These sorted TFs were probed for the 'presence of transcript in the ISH image datasets of the adult mouse brain from ABA' and *via* 'the intensity measurements of probes in human brain microarray data' [available at the Allen Human Brain Atlas, AHBA; **(S10 Table)** shows the list of probes used for analysis]. The purpose of this methodology was that if we can identify the functions for transcription factors that regulate galectins, we can propose/assign some functions to galectins based on their co-expression with the putative regulatory TFs.

Structures studied in mouse brain

Some major brain structures were studied in great detail in relation to the hierarchically organized substructures based on the reference atlas ontology, while others were studied as a single major structure without delving deeply into the subregions. For example, in the perspective of cortex (CTX), the focus was on the somatosensory area, where the expression profile from all its 6 layers was generated. In hippocampus (HP), all 3 divisions in the Ammon's horn (CA1-CA3) were studied, taking the individual layers also into account along with the dentate gyrus's (DG) sublayers, that provided information on the highly proliferative sub granular zone (SGZ) too. However, thalamus (THA), hypothalamus (HYP), pons (PO) and medulla (MY) were analyzed as main parent structures. The complete list of structures along with their respective substructures analyzed in each plane is provided in **S11 Table.** For the expression analysis in mouse using ISH images, the expression profile was created for sagittal sections moving from lateral to mid-sagittal plane. This was done to see if there was any heterogeneity or variation in gene expression from lateral to medial direction as different structures are located in different planes of the brain. For example, for hippocampus,

lateral sections in sagittal plane showed two hippocampi in the cortex, while in the para-sagittal section, only one hippocampus was completely visible. Another example was of the cerebellum, where in the lateral section, only cerebellar hemispheres were visible but in the mid-sagittal section, all the lobes of vermis were properly identifiable.

Expression heatmaps:

The expression factors were then plotted as heat maps to represent different structures and different genes according to the level of average expression factors using assigned categories. The heat map was plotted in R using the function heatmap.2 in gplots package [1]. The hierarchical clustering was preformed to identify the genes with similar expression levels across the brain sub-structures. Another round of application of the hierarchical clustering, incorporating the combined expression profiles of galectins and the putative regulatory transcription factors in mouse brain, further helped in elucidating the co-expressed genes and from this exercise, we could seek first predictive insights into the functions of galectins from their putative regulators, if they had similar expression in the same regions or in the same cell-types. This methodology also facilitated the identification of the co-expressed genes that were spatially restricted, hence showed regional variations.

Gene ontology annotation:

Gene list enrichment analysis for different biological processes was performed for each galectin and its possible regulatory transcription factors by using ToppGene suite, with FDR corrected values and P-value cutoff of 0.05 [2].

Immunohistochemistry protocol and Image capture settings:

Standard fluorescence immunohistochemistry protocol was followed for paraffin sections and TSA Amplification system was used for signal detection according to manufacturer instructions. A total of 6-7 brains from 8 weeks old C57Bl/6J male mice were sectioned in saggital plane and 3 appropriate representative sections from parasaggital, mid-saggital and lateral planes were processed for galectins immunostaining per brain. No primary incubation was kept as a negative control and 'Tuj1' neuronal marker antibody incubation was used as a positive control to test the specificity of the signal. The images were captured the subsequent day. The image capture setting, namely, exposure time, gain and offset, mercury lamp power, objectives were kept constant for all comparative set of experiments. The images were captured in 10X objective using Olympus IX73 fluorescence microscope. The captured

images were converted to indexed images for regional expression analysis by using the same 'user generated' LUT (Look Up Table) settings which were described for analysis of the *in situ* hybridization images. Brightness/contrast adjustments, if necessary were applied equally to every pixel in the images (i.e. maximum projections), for each comparative set using Fiji image analysis software. No change to gamma settings was applied. All data were obtained from at least 6-7 independent animals. For each animal, at least 3 images per galectin per plane of sectioning were recorded.

Microarray data for human from Allen Human Brain Atlas (AHBA):

The microarray-based gene expression profiles i.e. normalized log2 transformed values for each transcription factor and galectin were extracted from the Allen Human Brain Atlas (AHBA, http://human.brain-map.org/static/download) [3]. Initially all the probes present in AHBA for each gene were downloaded and then only that probe **(S10 Table)** was taken to represent the gene whose median expression across all 6 brains was the highest [4]. The data was then separated for each donor and the extra subregions which were present only in one of the donor, were removed **(S11 Table).** The Human Genome Organization Nomenclature Committee Database (HGNC) [5] was used to provide the correct gene names for the genes in the dataset. For convenience, donor brains were renamed as follows: H0351.2001 as B1, H0351.2002 as B2, H0351.1009 as B3, H0351.1012 as B4, H0351.1015 as B5, and H0351.1016 as B6.

Network construction

Assigned weighted gene co-expression network was constructed for each brain using R software [6]. First, a similarity matrix was generated based on the Pearson correlations between all gene pairs. Then, this correlation matrix was raised to a soft threshold power β=12 (default value for signed networks) to calculate a signed adjacency matrix, which reported the connection strengths between the node pairs. The selected power was used to exhibit scale-free topology [7]. Based on the adjacency matrix, a topological overlap measure (TOM) was calculated, which reflects how close the neighbors of a gene are to neighbors of the other gene i.e. the network interconnectedness [8]. TOM was used to calculate the measure of node dissimilarity (1-TOM), which was used as input for creating a hierarchical clustering tree that defined the network modules. For branch cutting, a dynamic tree cutting algorithm was used with minimum module size of 10 genes. Then modules with high

correlation were merged where the minimum height for merging modules was kept at 0.25. Each module was given unique color followed by assignment of numbers according to size and the unassigned genes were labeled in gray.

Each module was summarized by the first principal component of the module expression profile, the module eigengene (ME), which reflected the characteristic expression profile of a module. For each gene, module membership measure (kME) was defined as the correlation between gene expression values and the module eigengene (ME). To assess the statistical significance of module membership, a p-value was also calculated. Further derivation of intramodular connectivity measure helped in identifying the key players in the network which were the hub genes.

Weighted Gene Co-expression Network (WGCNA) package was used to enable identification of modules representing specific substructures. Module eigengenes were plotted at the level of substructures with the help of verbosen barplot function, which helped in identifying whether the module was enriched in some specific structure or not.

To enable easy comparison and visualization in the modules from different brains, module labels for different brains were relabeled using match Labels function according to the modules in the donor brain 1 (B1). This function compared the genes in the modules in both datasets i.e. from 2 donors using a hypergeometric test and then relabeled the module from the second brain, so that both the modules with significant number of common genes had the same label.

Module preservation statistics $Z_{summary}$ was calculated to assess the overlap between the modules from rest of the donor's w.r.t. the first donor (B1). The module preservation analysis uses permutations to provide the $Z_{summary}$ statistics, which summaries how much composition and structure was preserved in the rest of the donors as compared to B1 [9]. The $Z_{summary}$ statistics takes into account the overlap in module membership, the density (mean connectivity) and connectivity (sum of connections) patterns of modules, hence higher the preservation of $Z_{summary}$, the stronger the evidence that the module was preserved. A module showed no evidence of preservation if its $Z_{summary}$ was less than 2; whereas a $Z_{summary}$ of greater than 5 (or 10) indicated moderate (or strong) module preservation.

To know if the module membership was preserved with respect to each module in B2-B6 as compared to B1, kME comparison plot function in WGCNA package was used that plots the correlation of module membership between two brains according to the modules.

For gene list enrichment analysis of each module from each donor brain, we used ToppGene suite, where we identified different biological processes with FDR corrected values with a P-value cutoff of 0.05 [2]. Cytoscape web-package was further used to create module wise networks where the identified hubs were presented as the central molecules; the module color denoted the intramodule connectivity, size denoted the betweenness centrality and the edge denoted the weight of the connection between the two nodes.

Comparison between mouse and human brain for galectin family gene expression

To compare the expression pattern across human and mouse, common structures were found according to top structure classification from the human brain annotation as described in **S11 Table.**

REFERENCES:

1. **gplots: Various R Programming Tools for Plotting Data. R package version 2.17.0.** [http://CRAN.R-project.org/package=gplots]

2. Chen J, Bardes EE, Aronow BJ, Jegga AG: **ToppGene Suite for gene list enrichment analysis and candidate gene prioritization.** *Nucleic Acids Res* 2009, **37:**W305-311.

3. Hawrylycz MJ, Lein ES, Guillozet-Bongaarts AL, Shen EH, Ng L, Miller JA, van de Lagemaat LN, Smith KA, Ebbert A, Riley ZL, et al: **An anatomically comprehensive atlas of the adult human brain transcriptome.** *Nature* 2012, **489:**391-399.

4. Oliver KL, Lukic V, Thorne NP, Berkovic SF, Scheffer IE, Bahlo M: **Harnessing gene expression networks to prioritize candidate epileptic encephalopathy genes.** *PLoS One* 2014, **9:**e102079.

5. Gray KA, Yates B, Seal RL, Wright MW, Bruford EA: **Genenames.org: the HGNC resources in 2015.** *Nucleic Acids Res* 2015, **43:**D1079-1085.

6. Langfelder P, Horvath S: **WGCNA: an R package for weighted correlation network analysis.** *BMC Bioinformatics* 2008, **9:**559.

7. Zhang B, Horvath S: **A general framework for weighted gene co-expression network analysis.** *Stat Appl Genet Mol Biol* 2005, **4:**Article17.

8. Yip AM, Horvath S: **Gene network interconnectedness and the generalized topological overlap measure.** *BMC Bioinformatics* 2007, **8:**22.

9. Langfelder P, Luo R, Oldham MC, Horvath S: **Is my network module preserved and reproducible?** *PLoS Comput Biol* 2011, **7:**e1001057.

SUPPLEMENTARY FIGURE LEGENDS:

S1 Fig: Pseudocolor conversion of the original *in situ* hybridization images of the cortex and the olfactory bulb from the Allen Brain Atlas. (A) Original ISH images of galectins are shown with the pseudocolor converted images of the cortex, with individual cortical layer marked from L1 to L6. **(B)** Original ISH images of galectins are shown with the pseudocolor converted images of the olfactory bulb, different cell layers labeled as GL: glomerular layer, IPL: inner plexiform layer, OPL: outer plexiform layer, MCL: Mitral cell layer and GCL: granule cell layer.

S2 Fig: Pseudocolor conversion of the original *in situ* hybridization images of the hippocampus and the lateral ventricle from the Allen Brain Atlas. **(A)** Original ISH images of galectins with the pseudocolor converted images of the hippocampus, with individual layers marked as CA1: *Cornu Ammonis*1, CA2: *Cornu Ammonis*2, CA3: *Cornu Ammonis*3, DG: dentate gyrus, SGZ: sub granular zone. **(B)** Original ISH images of the galectins with the pseudocolor converted images of lateral ventricle where SVZ: sub ventricular zone, ChP: choroid plexus are indicated.

S3 Fig: Pseudocolor conversion of the original *in situ* hybridization images of the cerebellum from the Allen Brain Atlas with the custom pseudocolor. (A) Original ISH images of galectins with the pseudocolor converted images for the cerebellum, where DCN: deep cerebellar nuclei, WM: white matter, ML: molecular layer, GCL: granular cell layer, PCL: purkinje cell layer are highlighted.

S4 Fig: Expression of galectins and their putative regulatory TFs in the Cerebral Cortex. Pseudocolor converted images of galectins and their putative transcription factors in

the mouse cortex highlight the extent and levels of co-expression. A reference image from Allen Brain Atlas is shown to map the individual cortical layers from L1 to L6.

S5 Fig: Expression of galectins and putative regulatory TFs in the Olfactory Bulb. Pseudocolor converted images of galectins and their putative transcription factors in the mouse olfactory bulb highlight the extent and levels of co-expression. A reference image from Allen Brain Atlas is shown to map the individual cell layers labeled as GL: glomerular layer, IPL: inner plexiform layer, OPL: outer plexiform layer, MCL: Mitral Cell Layer and GCL: granule cell layer.

S6 Fig: Expression of galectins and putative regulatory TFs in the Basal Ganglia. Pseudocolor converted images of galectins and their putative transcription factors in the mouse basal ganglia highlight the extent and levels of co-expression. A reference image from Allen Brain Atlas is shown.

S7 Fig: Expression of galectins and putative regulatory TFs in the Hippocampus. Pseudocolor converted images of galectins and their putative transcription factors in the mouse basal ganglia highlight the extent and levels of co-expression. A reference image from Allen Brain Atlas is shown to map the individual cell layers labeled as CA1: *CornuAmmonis*1, CA2: *CornuAmmonis*2, CA3: *CornuAmmonis*3, DG: dentate gyrus, SGZ: sub granular zone.

S8 Fig: Expression of galectins and putative regulatory TFs in the Lateral Ventricles and SVG. Pseudocolor converted images of galectins and their putative transcription factors in the mouse lateral ventricles highlight the extent and levels of co-expression. A reference image from Allen Brain Atlas is shown to map the individual cell layers labeled SVZ: sub ventricular zone, ChP: choroid plexus.

S9 Fig: Expression of galectins and putative regulatory TFs in the Thalamus. Pseudocolor converted images of galectins and their putative transcription factors in the mouse thalamus highlight the extent and levels of co-expression. A reference image from Allen Brain Atlas is shown.

S10 Fig: Expression of galectins and putative regulatory TFs in the Cerebellum. Pseudocolor converted images of galectins and their putative transcription factors in the mouse cerebellum highlight the extent and levels of co-expression. A reference image from Allen Brain Atlas is shown to map the individual cell layers labeled as DCN: deep cerebellar nuclei, WM: white matter, ML: molecular layer, GCL: granular cell layer, PCL: purkinje cell layer.

S11 Fig: Expression of galectins and putative regulatory TFs the Substantia nigra. Pseudocolor converted images of galectins and their putative transcription factors in the mouse substantia nigra highlight the extent and levels of co-expression. A reference image from Allen Brain Atlas is shown.

S12 Fig: Representative Image of Lgals9-TF hierarchical cluster: Pseudocolor converted images for one of the cluster obtained after performing hierarchical clustering for expression data of galectin-9 with its putative regulatory transcription factors in the parasaggital plane. A. cortex, B. olfactory bulb, C. hippocampus, D. lateral ventricle, E. cerebellum.

S13 Fig: Heat map of gene expression in Mid-sagittal plane. **(A)** Heat map of galectins in mouse showing the average expression in each sub-structure arranged by parent structures. Expression factor of GAPDH is shown as a reference. **(B)** Heat map of galectins with their putative transcription factors showing expression correlations after hierarchical clustering at the level of both gene and structure.

S14 Fig: Heat map of gene expression in Lateral plane. **(A)** Heat map of galectins in mouse showing the average expression in each sub-structure arranged by parent structures. Expression factor of GAPDH is shown as a reference. **(B)** Heat map of galectins with their putative transcription factors showing expression correlations after hierarchical clustering at the level of both gene and structure.

S15 Fig: Comparison of galectin-12 mRNA expression mask from the Allen Brain Atlas, Custom Mask used in this study and corroboration with protein expression. **(A)** Raw ISH image from Allen Brain Atlas, (**B)** ISH expression mask from the Allen Brain atlas, **(C)** ISH expression mask generated with our methodology for the same image, **(D)** confocal microscopy images of protein expression for galectin-12 in P56 mouse brain (male). (**F)-(N)**

Zoomed images of *Lgals-12* protein expression in major regions of the same mouse brain (*Lgals12*-green, DAPI-red). Please note that same plane of section, same age, same gender and same mouse strain has been used for precise comparisons. Clearly, regional protein expression data of galectin-12 matched well with our mask. *Please also note that due to secretory nature of galectins, protein expression is more widespread in comparison to respective transcript profiling of producer cell*s.

S16 Fig: Detailed Supplementary methods and examples for LUT generation; Intensity, Density and Expression factor calculation.

SUPPLEMENTARY TABLES LEGENDS:

S1 Table: List of URLs directing to the Allen Brain Mouse Atlas ISH image data extracted for mouse galectins.

S2 Table: TRANSFAC generated list of transcription factors that putatively regulate mouse galectins

S3 Table: Functions of galectins' putative regulatory transcription factors identified through literature search. Entries highlighted in yellow show genes for which Allen Brain Atlas had failed image quality control criteria. Entries highlighted in green, show no that there was no evidence for those genes in brain functions. Table shows the functions of each transcription factor with their literature references.

S4 Table: Allen Mouse Brain Atlas ISH image analysis for galectins and their putative regulatory transcription factors with GAPDH as reference gene. Orange highlights represents Lateral plane, Green represents Para-sagittal plane & Blue represents Mid-sagittal plane.

S5 Table: Mouse GO analysis for prediction of the functions of galectins from the known functions of their putative transcription factors: Gene enrichment analysis for each galectin and their possible transcriptional regulators in mouse using TOPPGene for biological processes with FDR corrected values and P-values less than 0.05.

S6 Table: Context dependent sorting of Mouse GO analysis for prediction of the functions of galectins from the known functions of their putative transcription factors. Sorting of Gene enrichment analysis for each galectin and their possible transcriptional regulators in relevance to brain processes.

S7 Table: List of predicted transcription factors regulating respective galectins in human.

S8 Table: List of predicted transcription factors common to both mouse and human, yellow color shows TFs with failed quality control ISH images in mouse.

S9 Table: List of predicted transcription factors in human which regulate galectins. Table shows the functions of each transcription factor with their references.

S10 Table: List of gene probes used in the Allen human brain atlas for galectins and their putative TFs.

S11 Table: Allen human brain atlas list of structures (data) available from each donor brain.

S12 Table: Module membership (kME) values for each gene corresponding to their respective module for each brain shown in separate worksheets.

S13 Table: Intramodular connectivity for each brain shown in separate worksheets.

S14 Table: List of genes belonging to each module for each brain shown in separate worksheets.

S15 Table: Module preservation statistics between donor brain 1 (B1) *vs.* other donor brains (B2-B6) shown in separate worksheets.

S16 Table: Human GO analysis for the prediction of the functions of galectins from the known functions of their putative regulatory transcription factors: Gene enrichment analysis

for modules represented in **Fig 9A-B** using TOPPGene for biological processes with FDR corrected values and P-values less than 0.05.

S17 Table: Context dependent sorting of Human GO analysis for the prediction of the functions of galectins from the known functions of their putative transcription factors in specific modules: Sorting of Gene enrichment analysis for each galectin and their possible transcriptional regulators in relevance to brain processes.

S18 Table: Summary of Brain Processes associated with Turquoise Module: List of brain processes associated with galectins' putative regulatory transcription factors in turquoise module for individual donor brain (6 donors). This module is associated with galectin 1,-2,-8 and -9.

S19 Table: Summary of Brain Processes associated with Brown Module: List of brain processes associated with galectins' putative regulatory transcription factors in brown module for individual donor brain (6 donors). This module is associated with galectin-13 and to less extent with galectin-16.

S20 Table: Summary of Brain Processes associated with Blue Module: List of brain processes associated with galectins' putative regulatory transcription factors in blue module of individual donor brain (6 donors). This module is associated with galectin-3 and to less extent with galectin-12.

S21 Table: Summary of Brain Processes associated with Green Module: List of brain processes associated with galectins' putative regulatory transcription factors in green module of individual donor brain (6 donors). This module is associated with galectin-4,-14 and to less extent with galectin-7.

S22 Table: WGCNA analysis of human brain microarray data from Allen Brain Human Atlas: Expression levels of different genes in individual brains (B1-B6) from 6 donors. List of genes and brain structures and substructures for transcript expression is described (including galectins) is systematically presented in separate worksheets.
