# Supplementary material for: mRNA Transcriptomics of Galectins Unveils Heterogeneous Organization in Mouse and Human Brain
Source: Front Mol Neurosci. 2016 Dec 16;9:139. doi: 10.3389/fnmol.2016.00139 (PMC5159438; doi:10.3389/fnmol.2016.00139)
Supplement: Supplementary file 2 [file Data_Sheet_2.PDF]

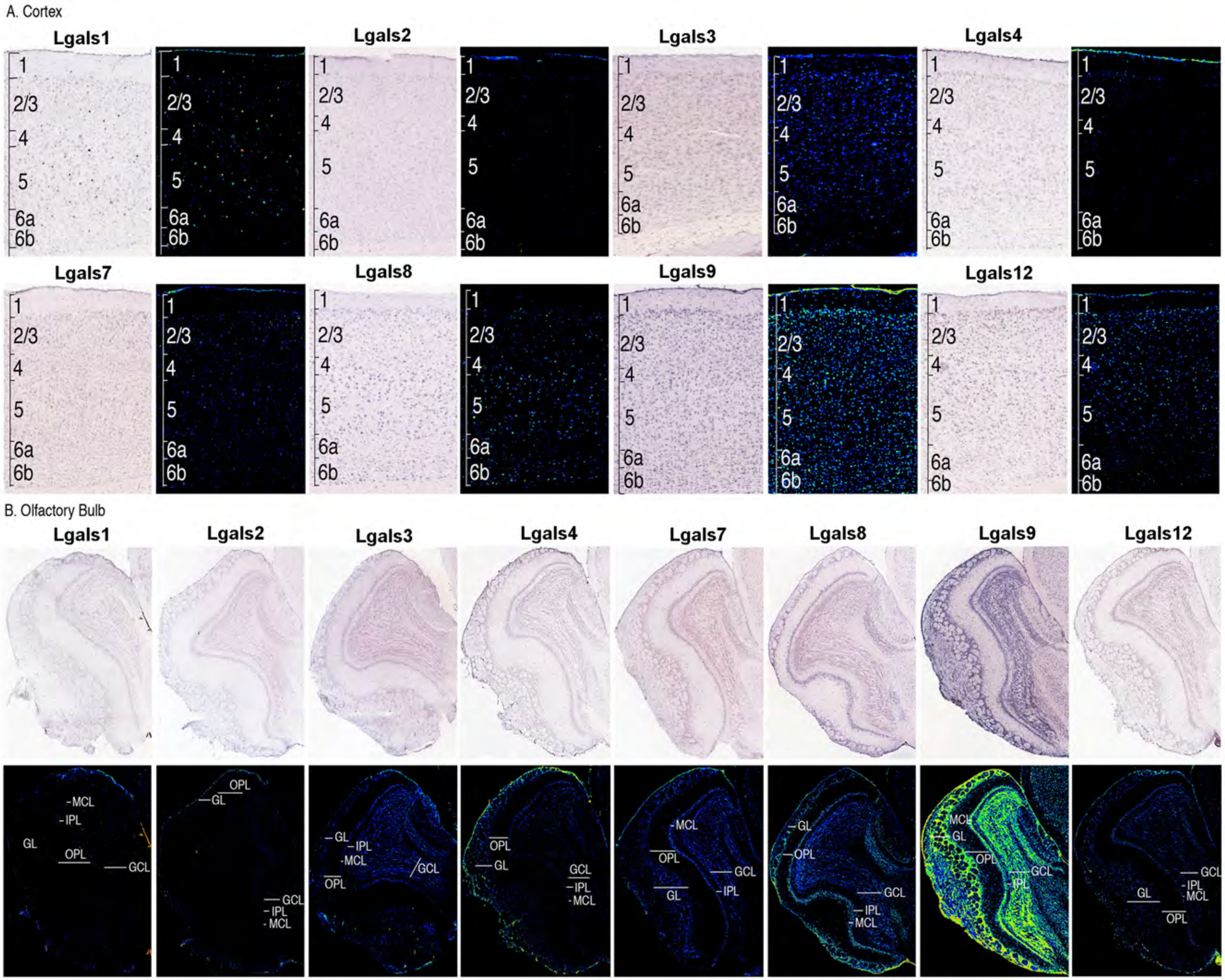
A. Cortex
Lgals1
Lgals2
Lgals3
Lgals4
Lgals7
Lgals8
Lgals9
Lgals12
1
2/3
4
5
6a
6b
B. Olfactory Bulb
Lgals1
Lgals2
Lgals3
Lgals4
Lgals7
Lgals8
Lgals9
Lgals12
GL
MCL
IPL
OPL
GCL

S1 Fig

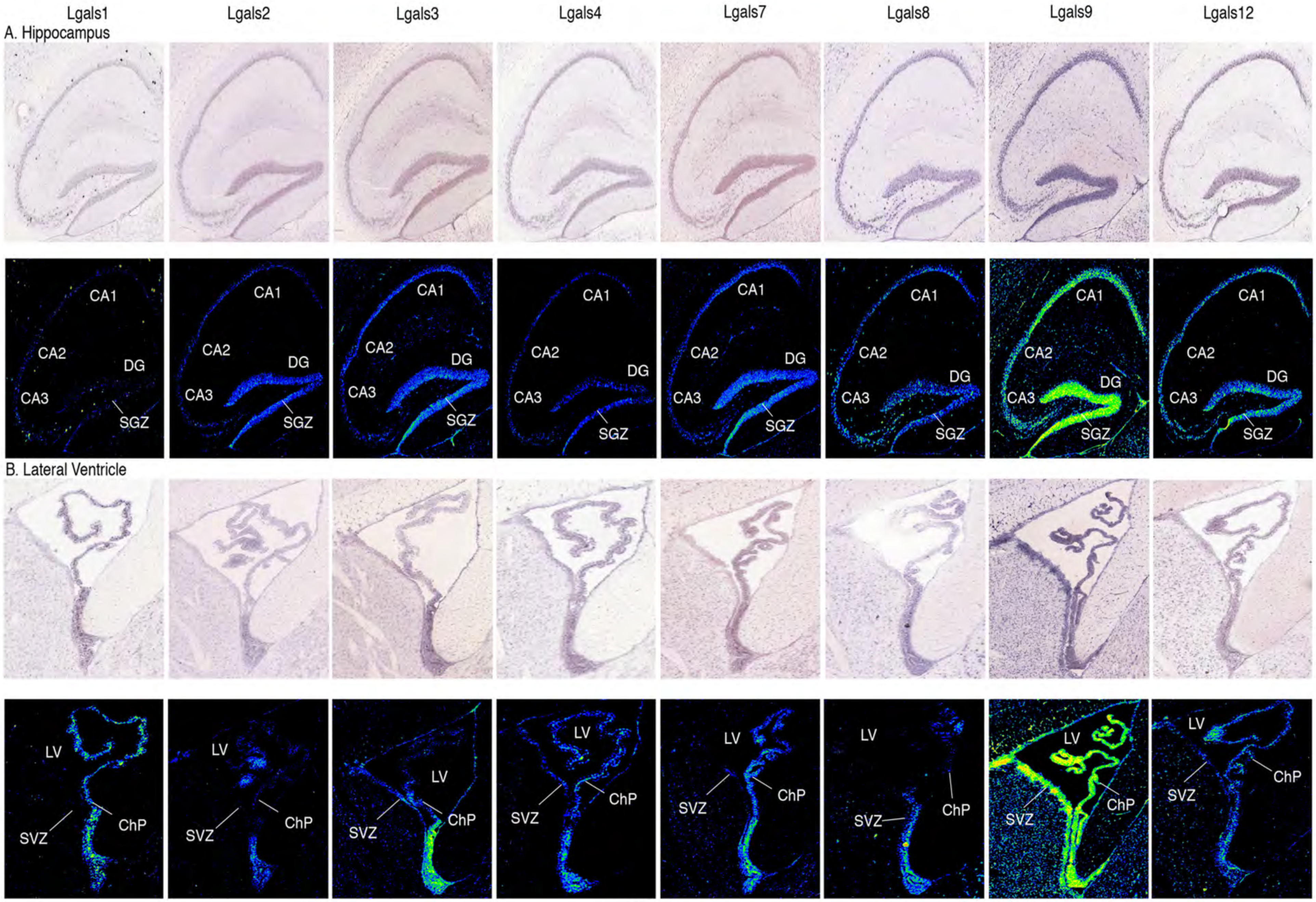
Lgals1
Lgals2
Lgals3
Lgals4
Lgals7
Lgals8
Lgals9
Lgals12
A. Hippocampus
CA1
CA2
CA3
DG
SGZ
B. Lateral Ventricle
LV
SVZ
ChP

S2 Fig

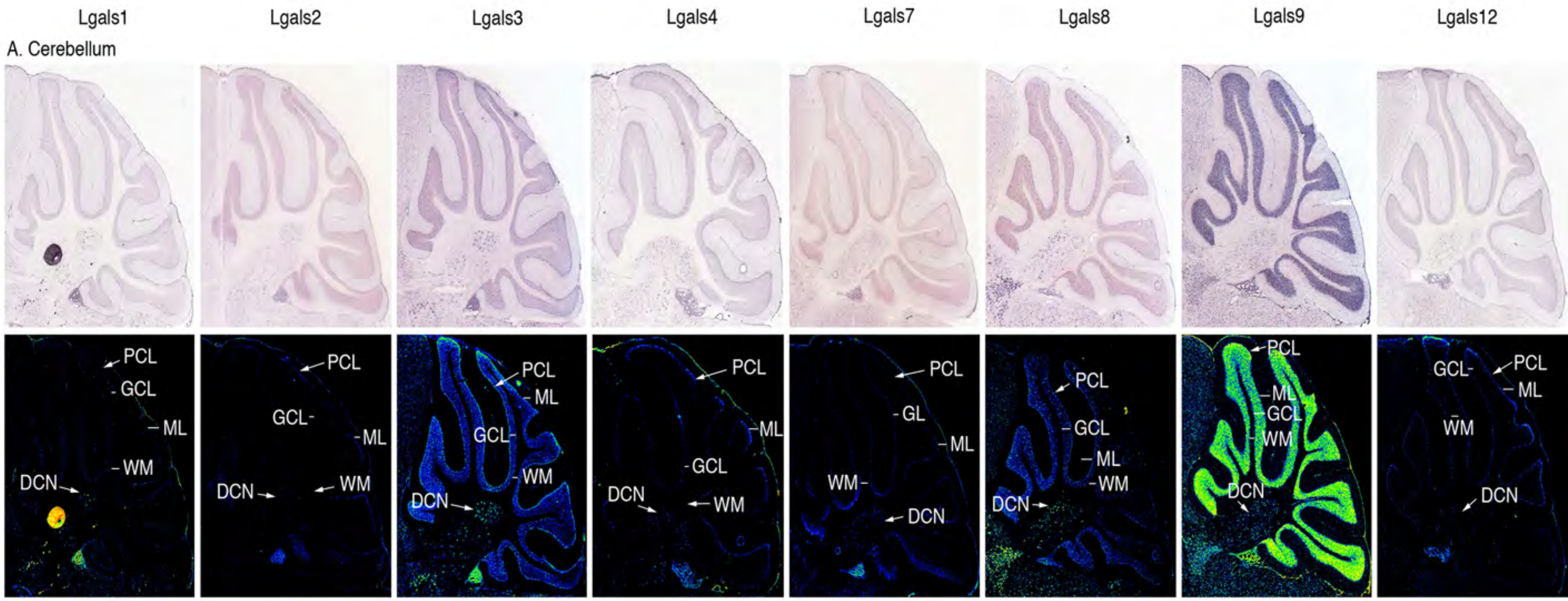
Lgals1
Lgals2
Lgals3
Lgals4
Lgals7
Lgals8
Lgals9
Lgals12
A. Cerebellum
PCL
GCL
ML
WM
DCN
GL

S3 Fig

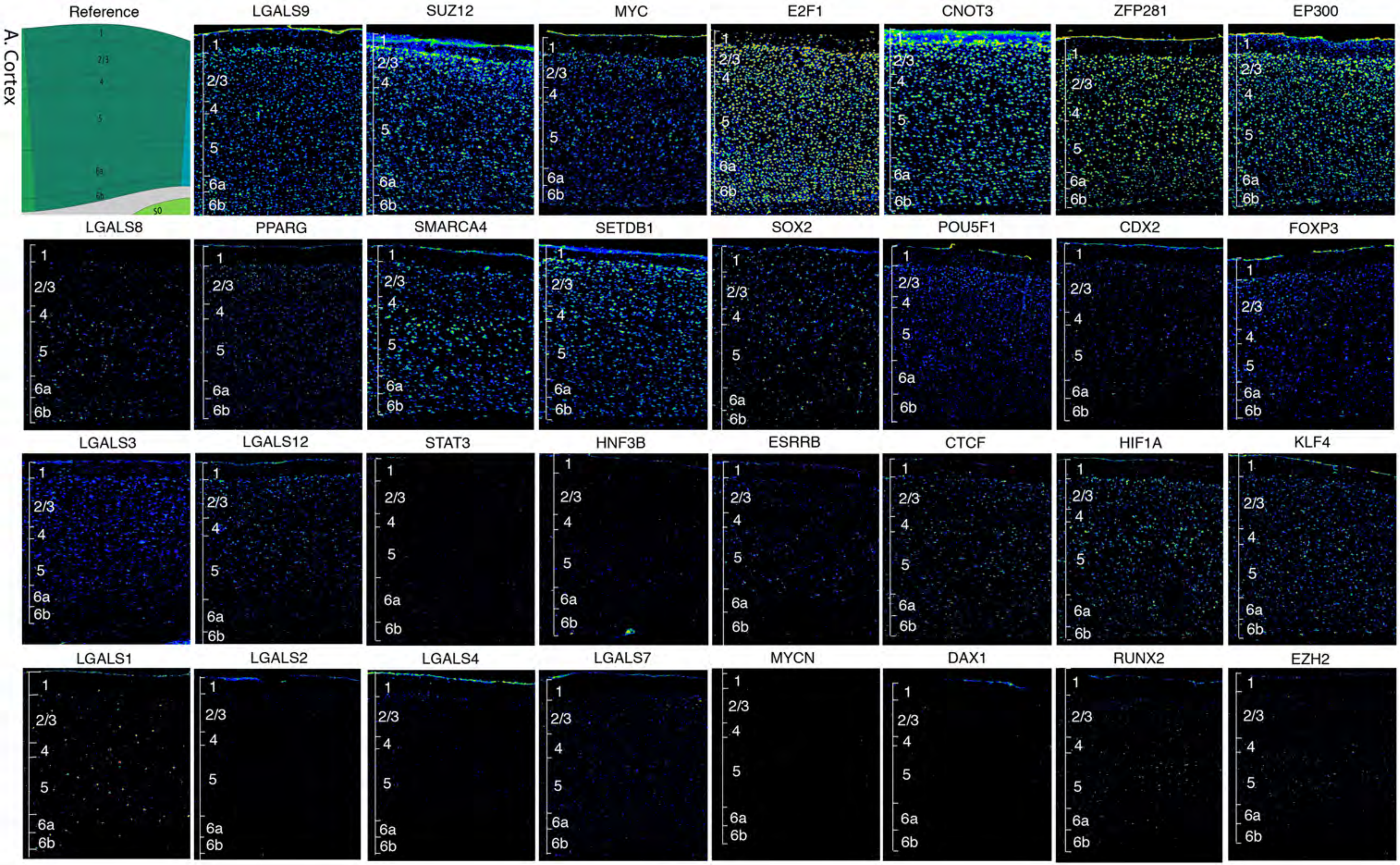
A. Cortex
Reference
LGALS9
SUZ12
MYC
E2F1
CNOT3
ZFP281
EP300
LGALS8
PPARG
SMARCA4
SETDB1
SOX2
POU5F1
CDX2
FOXP3
LGALS3
LGALS12
STAT3
HNF3B
ESRRB
CTCF
HIF1A
KLF4
LGALS1
LGALS2
LGALS4
LGALS7
MYCN
DAX1
RUNX2
EZH2
1
2/3
4
5
6a
6b
SO

S4 Fig

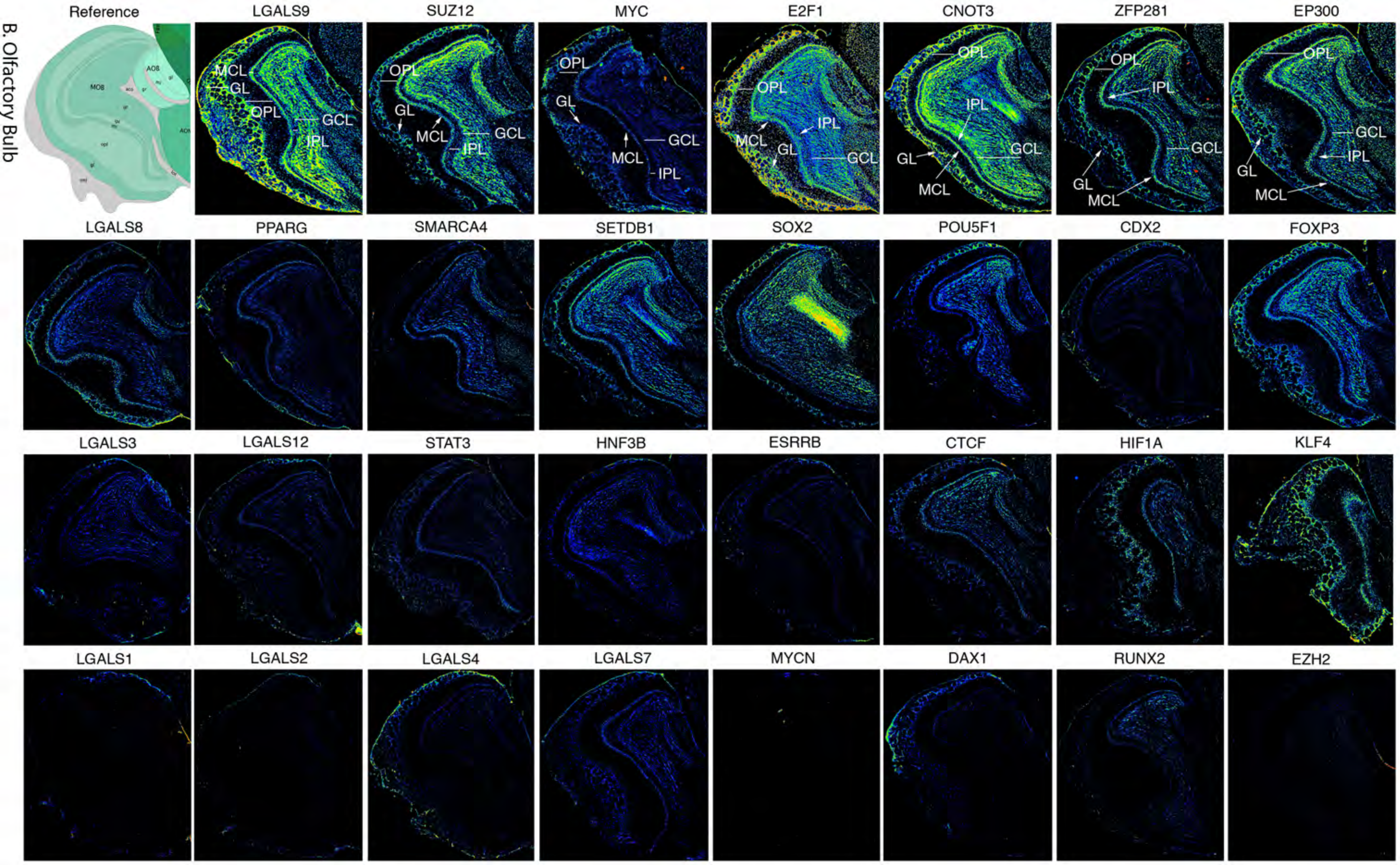
B. Olfactory Bulb
Reference
LGALS9
SUZ12
MYC
E2F1
CNOT3
ZFP281
EP300
OPL
MCL
GL
GCL
IPL
LGALS8
PPARG
SMARCA4
SETDB1
SOX2
POU5F1
CDX2
FOXP3
LGALS3
LGALS12
STAT3
HNF3B
ESRRB
CTCF
HIF1A
KLF4
LGALS1
LGALS2
LGALS4
LGALS7
MYCN
DAX1
RUNX2
EZH2

S5 Fig

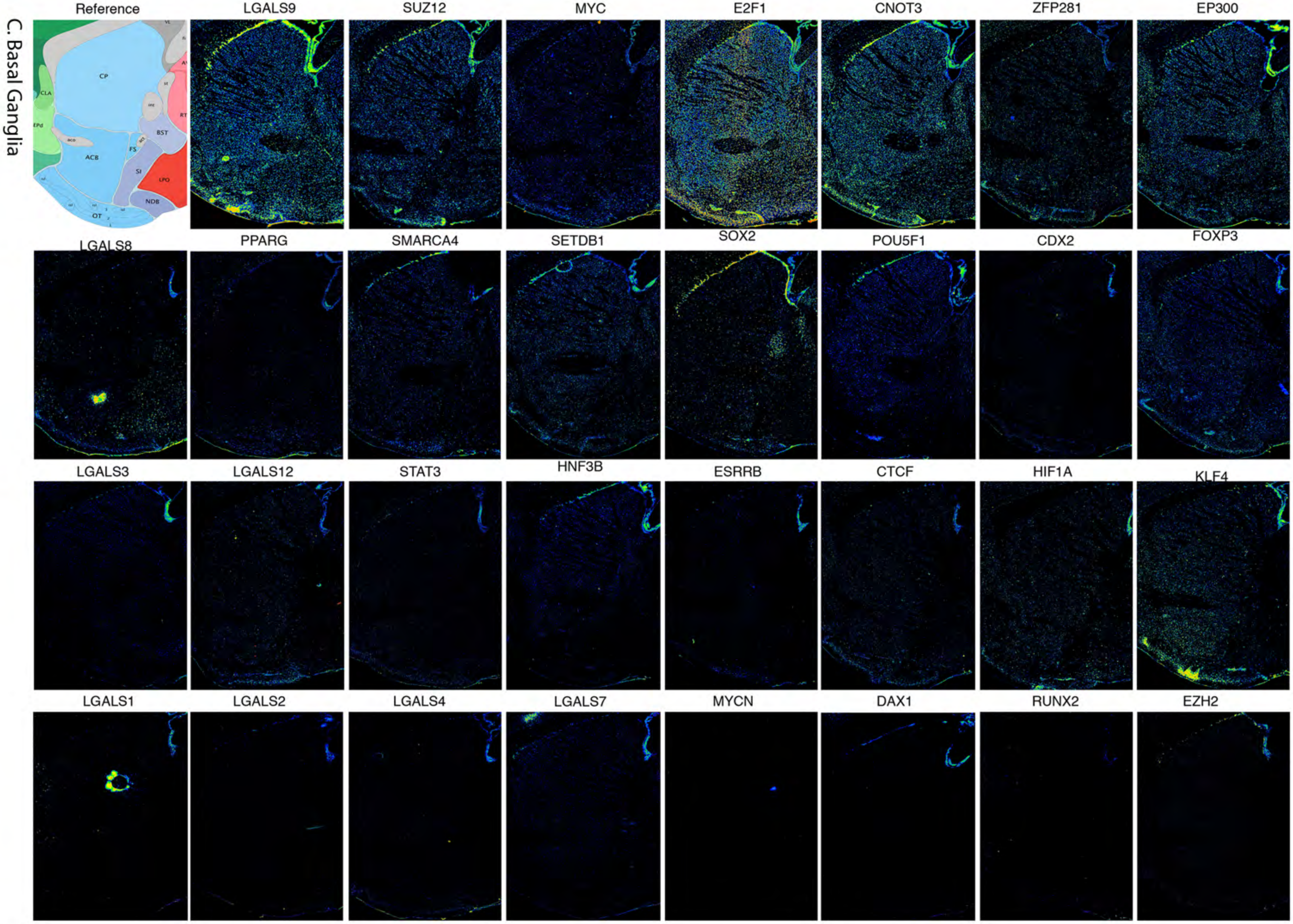
C. Basal Ganglia
Reference
LGALS9
SUZ12
MYC
E2F1
CNOT3
ZFP281
EP300
LGALS8
PPARG
SMARCA4
SETDB1
SOX2
POU5F1
CDX2
FOXP3
LGALS3
LGALS12
STAT3
HNF3B
ESRRB
CTCF
HIF1A
KLF4
LGALS1
LGALS2
LGALS4
LGALS7
MYCN
DAX1
RUNX2
EZH2

S6 Fig

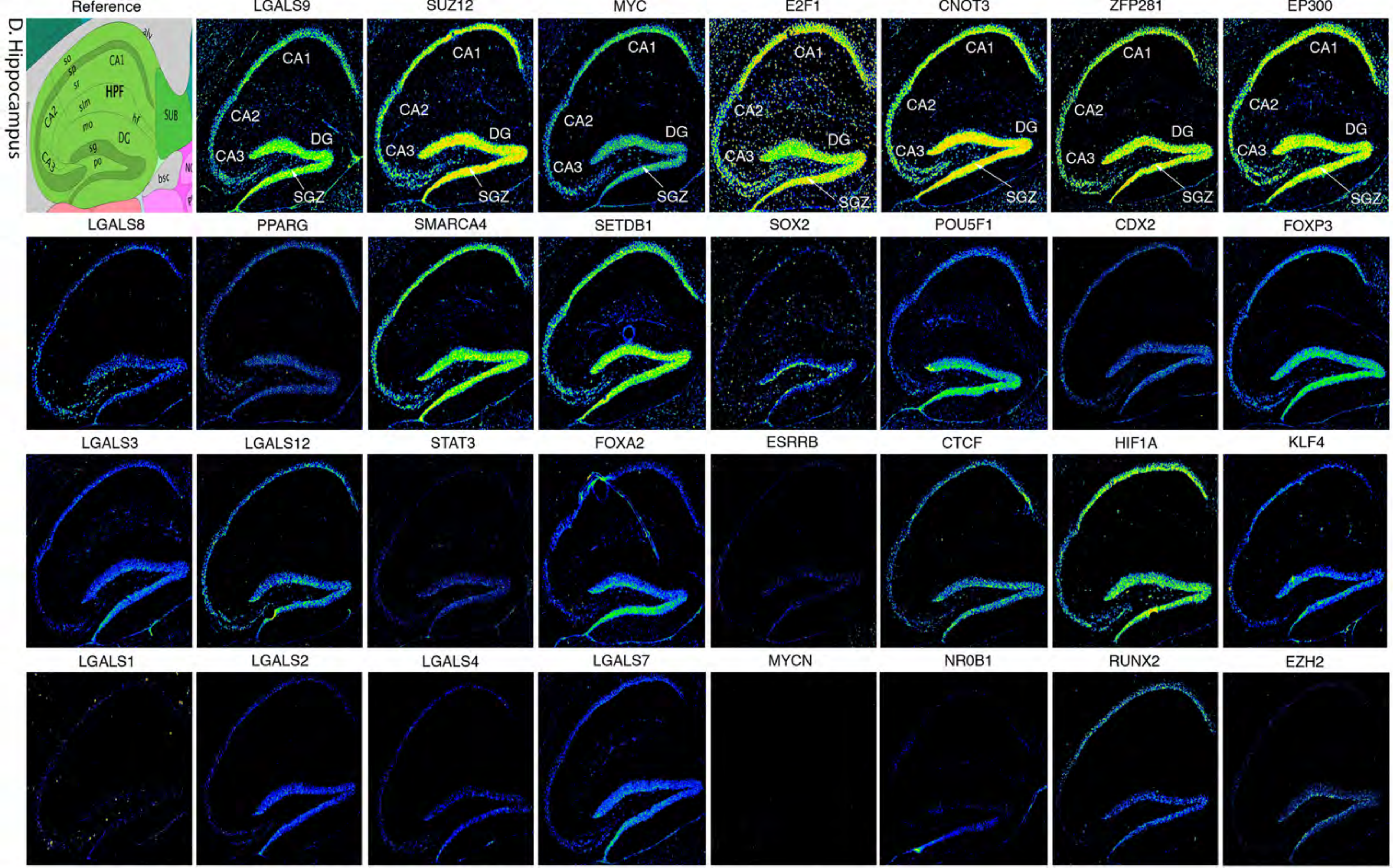
D. Hippocampus
Reference
alv
so
sp
sr
CA1
HPF
slm
CA2
mo
hf
SUB
DG
sg
po
CA3
bsc
LGALS9
SUZ12
MYC
E2F1
CNOT3
ZFP281
EP300
CA1
CA2
CA3
DG
SGZ
LGALS8
PPARG
SMARCA4
SETDB1
SOX2
POU5F1
CDX2
FOXP3
LGALS3
LGALS12
STAT3
FOXA2
ESRRB
CTCF
HIF1A
KLF4
LGALS1
LGALS2
LGALS4
LGALS7
MYCN
NR0B1
RUNX2
EZH2

S7 Fig

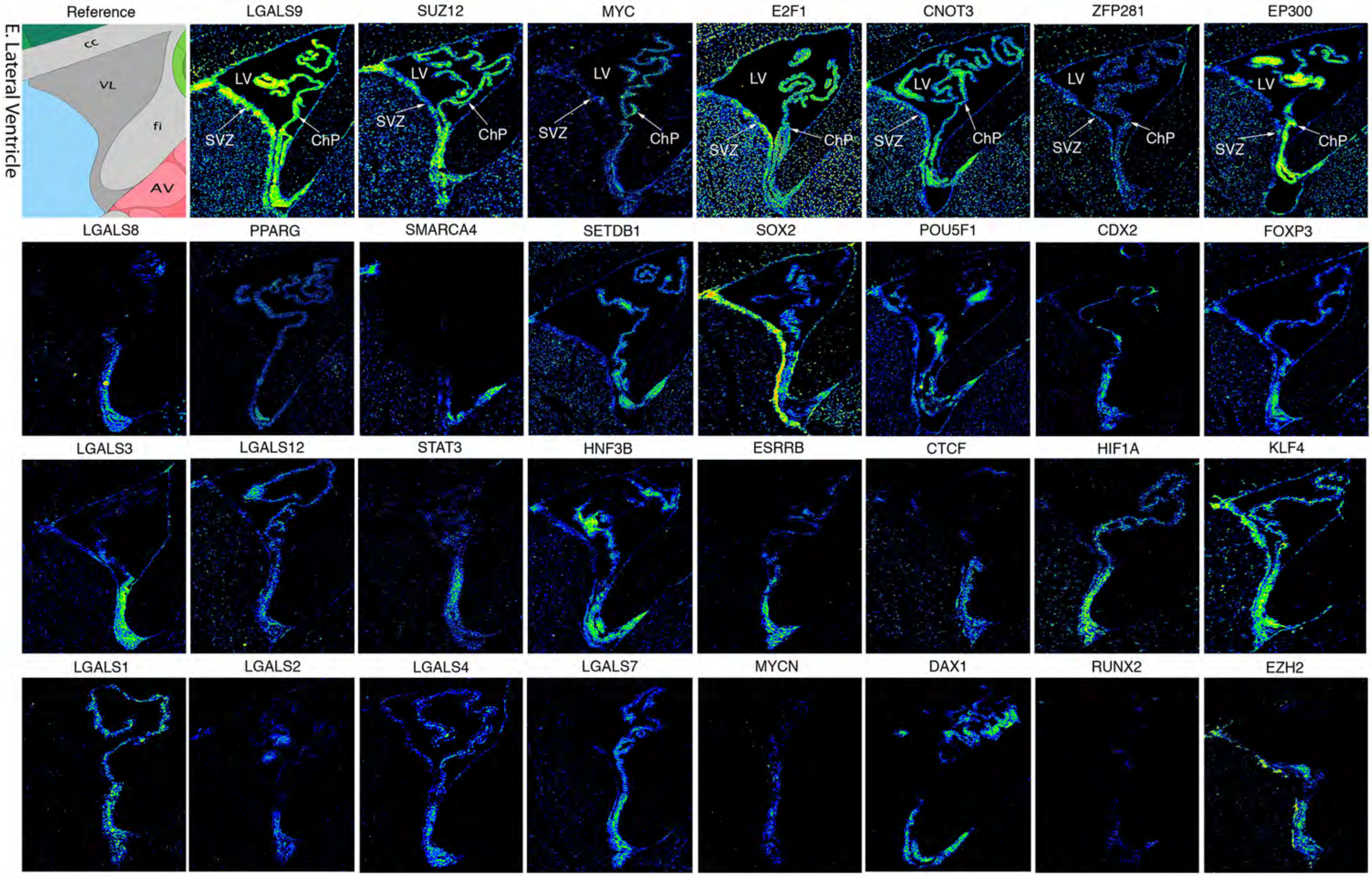
E. Lateral Ventricle
Reference
cc
VL
fi
AV
LGALS9
SUZ12
MYC
E2F1
CNOT3
ZFP281
EP300
LV
SVZ
ChP
LGALS8
PPARG
SMARCA4
SETDB1
SOX2
POU5F1
CDX2
FOXP3
LGALS3
LGALS12
STAT3
HNF3B
ESRRB
CTCF
HIF1A
KLF4
LGALS1
LGALS2
LGALS4
LGALS7
MYCN
DAX1
RUNX2
EZH2

S8 Fig

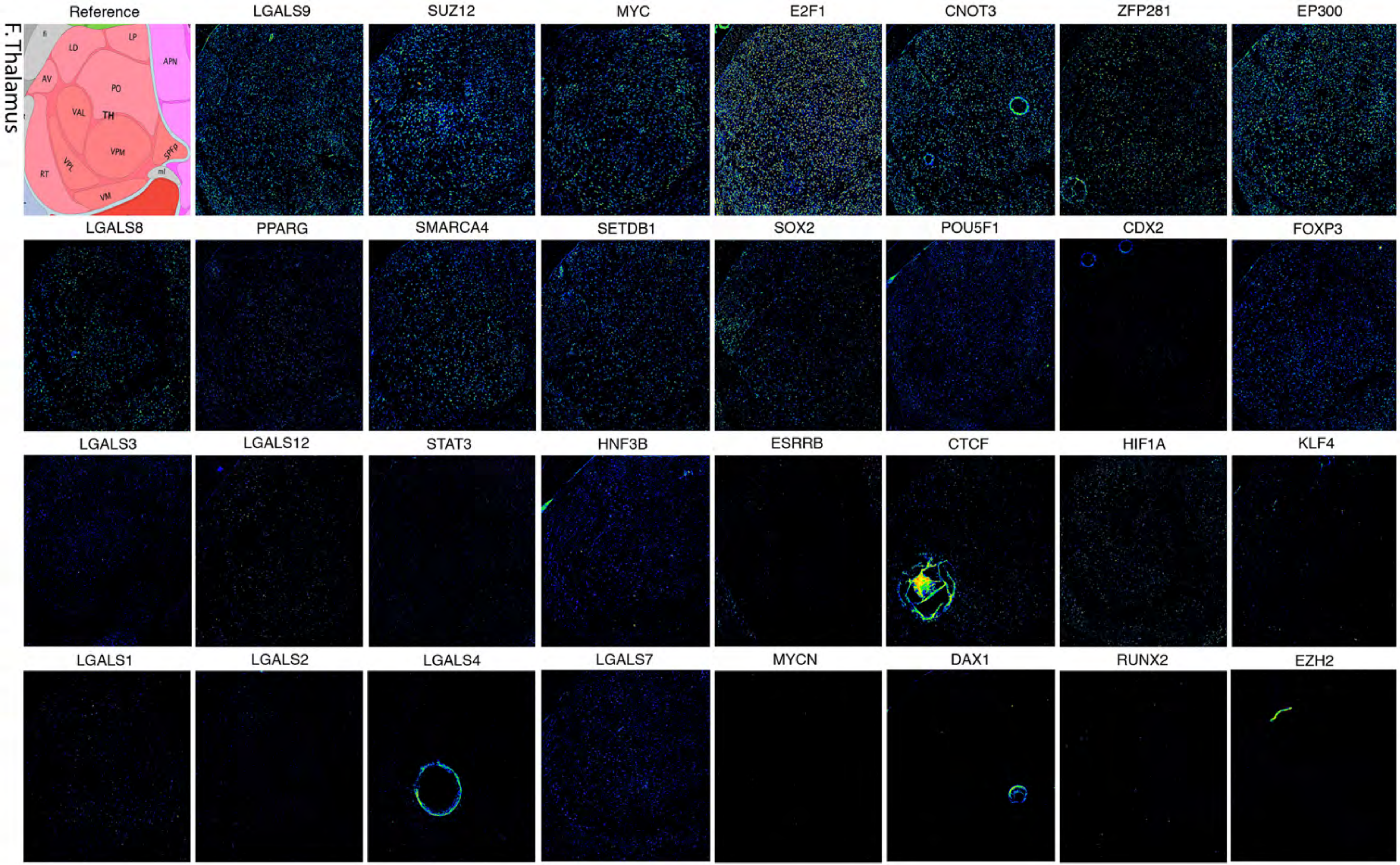
F. Thalamus
Reference
fi
LD
LP
APN
AV
PO
VAL
TH
VPM
SPFP
VPL
RT
ml
VM
LGALS9
SUZ12
MYC
E2F1
CNOT3
ZFP281
EP300
LGALS8
PPARG
SMARCA4
SETDB1
SOX2
POU5F1
CDX2
FOXP3
LGALS3
LGALS12
STAT3
HNF3B
ESRRB
CTCF
HIF1A
KLF4
LGALS1
LGALS2
LGALS4
LGALS7
MYCN
DAX1
RUNX2
EZH2

S9 Fig

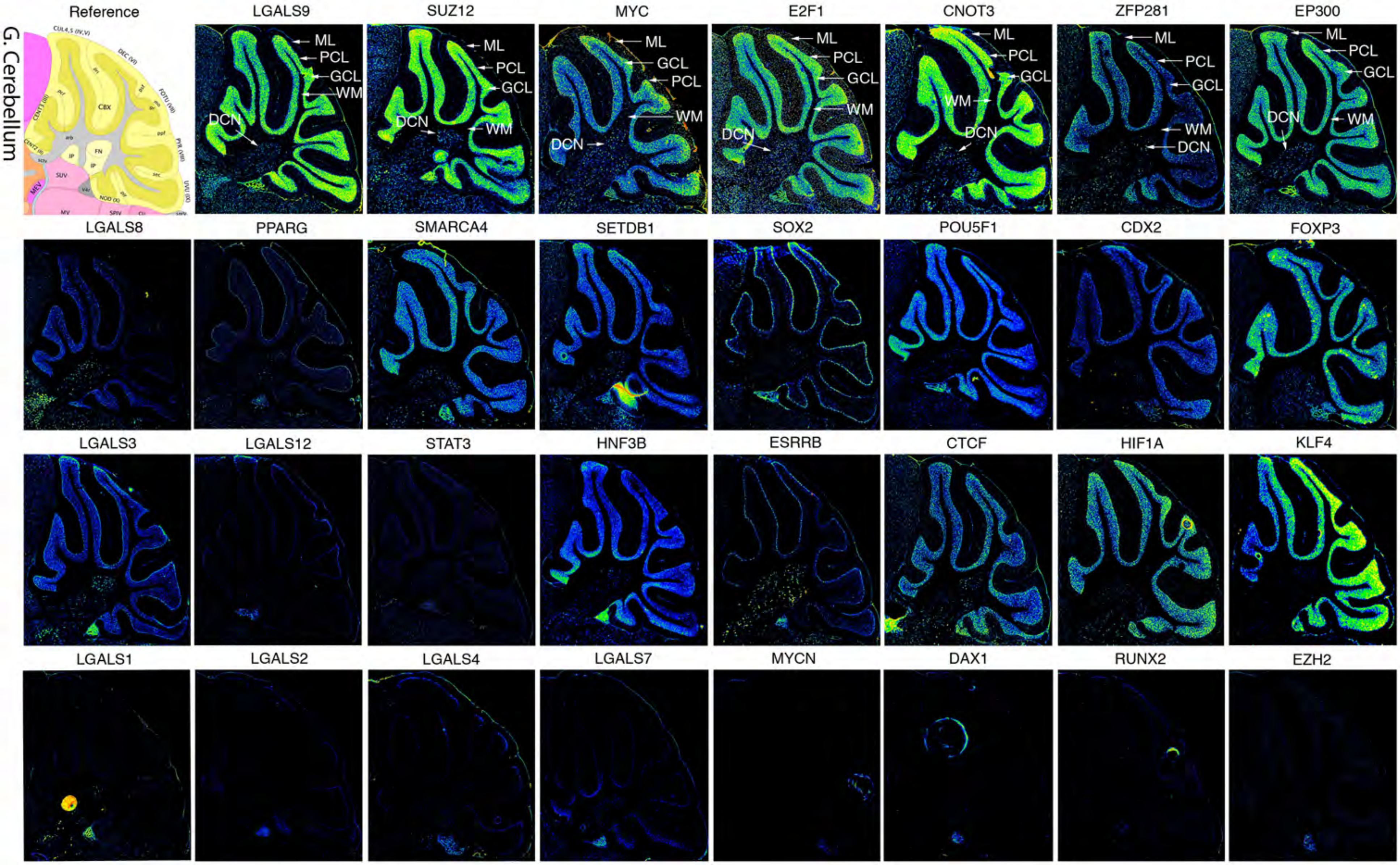
G. Cerebellum
Reference
LGALS9
SUZ12
MYC
E2F1
CNOT3
ZFP281
EP300
ML
PCL
GCL
WM
DCN
LGALS8
PPARG
SMARCA4
SETDB1
SOX2
POU5F1
CDX2
FOXP3
LGALS3
LGALS12
STAT3
HNF3B
ESRRB
CTCF
HIF1A
KLF4
LGALS1
LGALS2
LGALS4
LGALS7
MYCN
DAX1
RUNX2
EZH2

S10 Fig

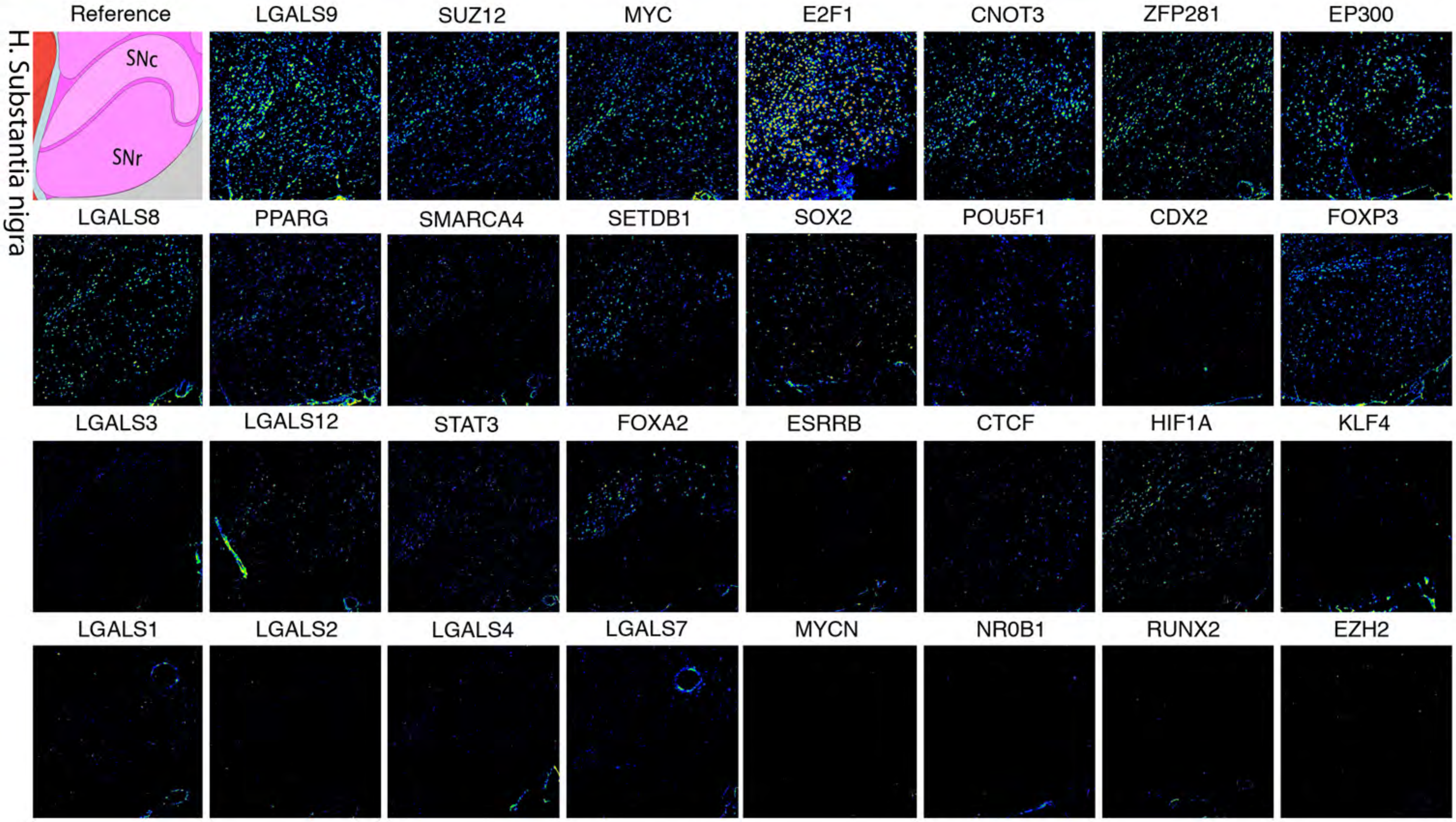
H. Substantia nigra
Reference
SNc
SNr
LGALS9
SUZ12
MYC
E2F1
CNOT3
ZFP281
EP300
LGALS8
PPARG
SMARCA4
SETDB1
SOX2
POU5F1
CDX2
FOXP3
LGALS3
LGALS12
STAT3
FOXA2
ESRRB
CTCF
HIF1A
KLF4
LGALS1
LGALS2
LGALS4
LGALS7
MYCN
NR0B1
RUNX2
EZH2

S11 Fig

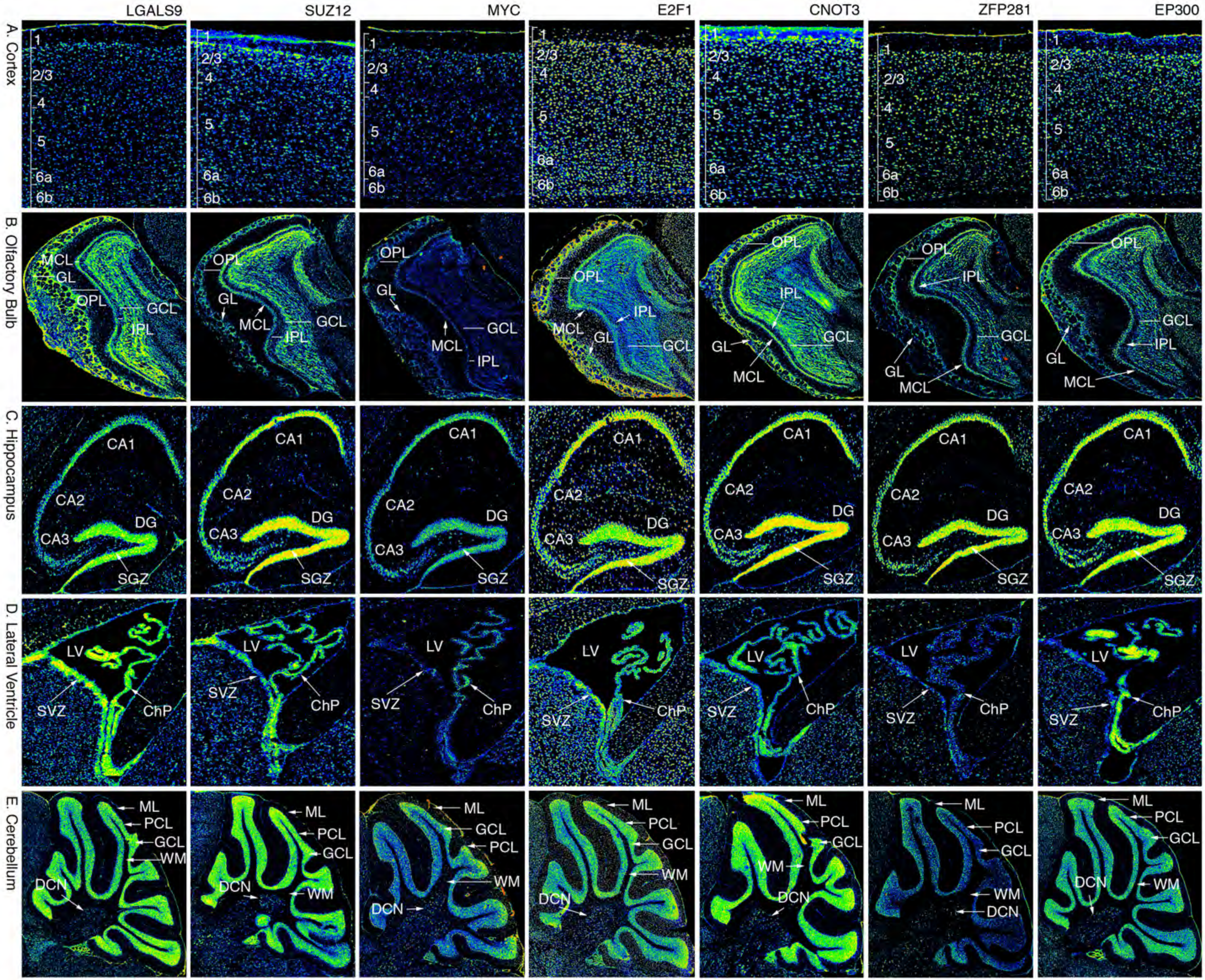
LGALS9
SUZ12
MYC
E2F1
CNOT3
ZFP281
EP300
A. Cortex
1
2/3
4
5
6a
6b
B. Olfactory Bulb
MCL
GL
OPL
GCL
IPL
C. Hippocampus
CA1
CA2
CA3
DG
SGZ
D. Lateral Ventricle
LV
SVZ
ChP
E. Cerebellum
ML
PCL
GCL
WM
DCN

S12 Fig

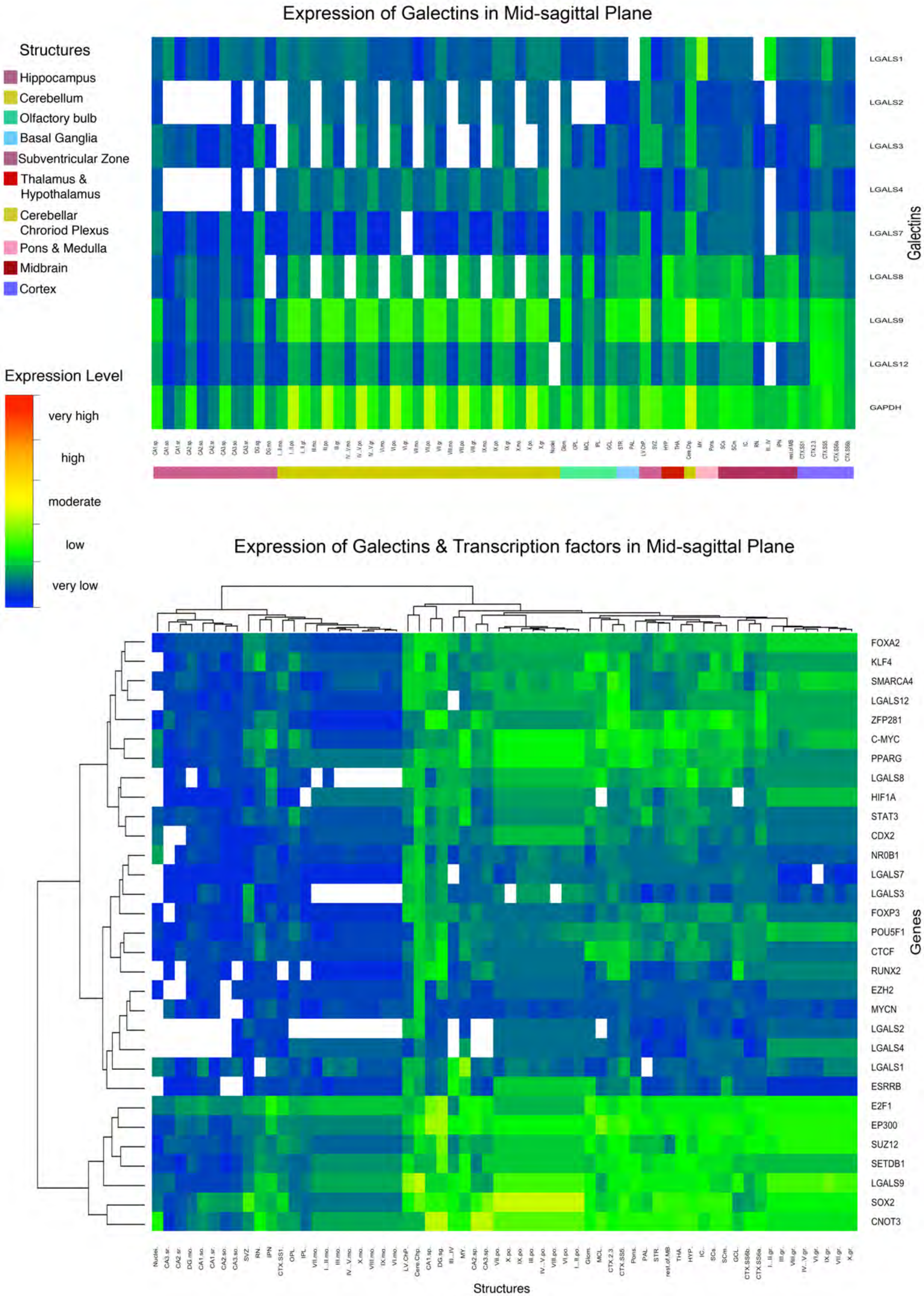
Expression of Galectins in Mid-sagittal Plane
Structures
Hippocampus
Cerebellum
Olfactory bulb
Basal Ganglia
Subventricular Zone
Thalamus & Hypothalamus
Cerebellar Chroriod Plexus
Pons & Medulla
Midbrain
Cortex
Expression Level
very high
high
moderate
low
very low
LGALS1
LGALS2
LGALS3
LGALS4
LGALS7
LGALS8
LGALS9
LGALS12
GAPDH
Galectins
Expression of Galectins & Transcription factors in Mid-sagittal Plane
FOXA2
KLF4
SMARCA4
LGALS12
ZFP281
C-MYC
PPARG
LGALS8
HIF1A
STAT3
CDX2
NR0B1
LGALS7
LGALS3
FOXP3
POU5F1
CTCF
RUNX2
EZH2
MYCN
LGALS2
LGALS4
LGALS1
ESRRB
E2F1
EP300
SUZ12
SETDB1
LGALS9
SOX2
CNOT3
Genes
Structures

S13 Fig

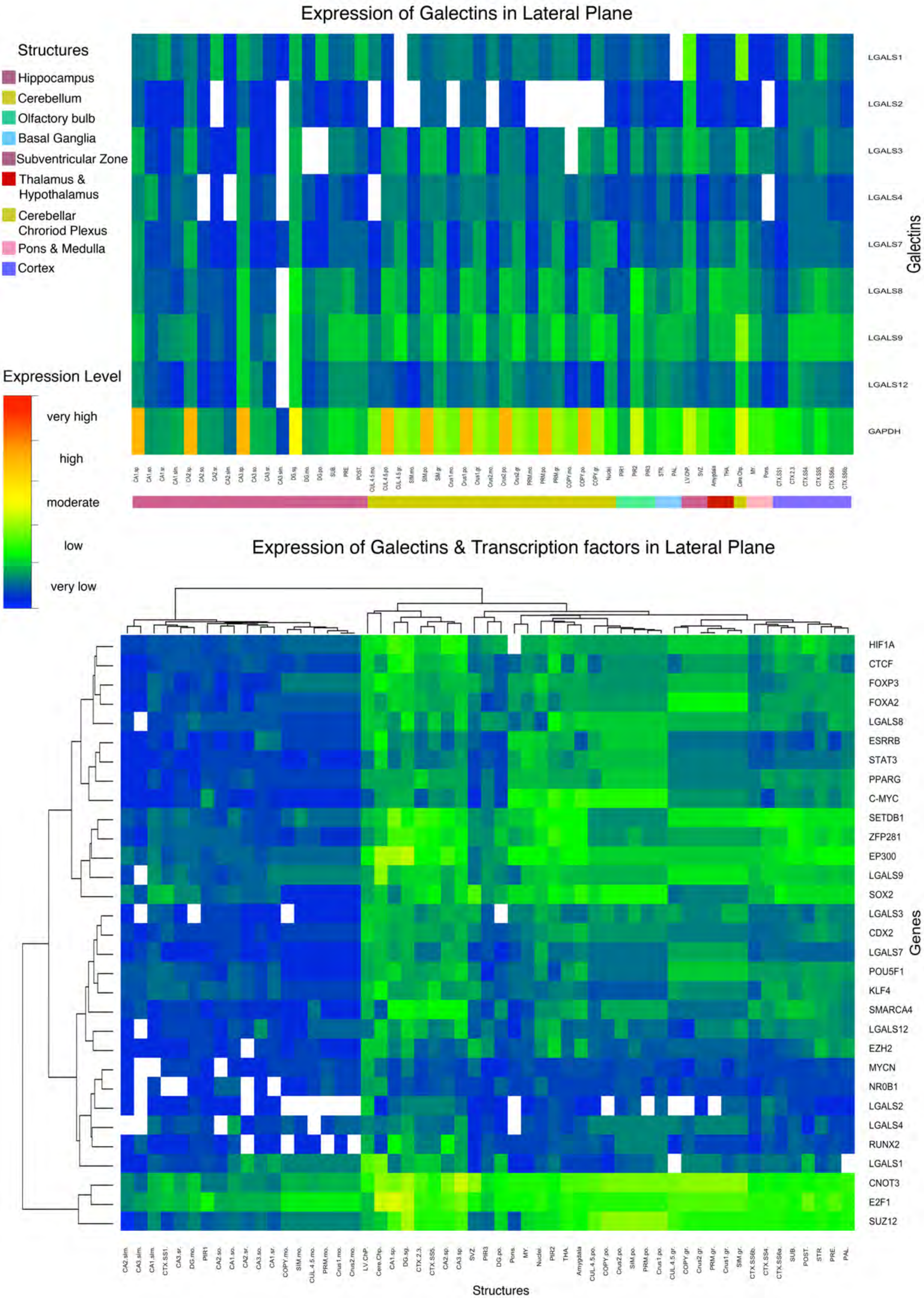
Expression of Galectins in Lateral Plane
Structures
Hippocampus
Cerebellum
Olfactory bulb
Basal Ganglia
Subventricular Zone
Thalamus & Hypothalamus
Cerebellar Chroriod Plexus
Pons & Medulla
Cortex
Expression Level
very high
high
moderate
low
very low
LGALS1
LGALS2
LGALS3
LGALS4
LGALS7
LGALS8
LGALS9
LGALS12
GAPDH
Galectins
Expression of Galectins & Transcription factors in Lateral Plane
HIF1A
CTCF
FOXP3
FOXA2
LGALS8
ESRRB
STAT3
PPARG
C-MYC
SETDB1
ZFP281
EP300
LGALS9
SOX2
LGALS3
CDX2
LGALS7
POU5F1
KLF4
SMARCA4
LGALS12
EZH2
MYCN
NR0B1
LGALS2
LGALS4
RUNX2
LGALS1
CNOT3
E2F1
SUZ12
Genes
CA2.sim.
CA3.sim.
CA1.sim.
CTX.SS1.
CA3.sr.
DG.mo.
PIR1
CA2.so.
CA1.so.
CA2.sr.
CA3.so.
CA1.sr.
COPY.mo.
SIM.mo.
CUL.4.5.mo.
PRM.mo.
Crus1.mo.
Crus2.mo.
LV.ChP.
Cere.Chp.
CA1.sp.
DG.sg.
CTX.2.3.
CTX.SS5.
CA2.sp.
CA3.sp.
SVZ
PIR3
DG.po.
Pons
MY.
Nuclei
PIR2
THA.
Amygdala
CUL.4.5.po.
COPY.po.
Crus2.po.
SIM.po.
PRM.po.
Crus1.po.
CUL.4.5.gr.
COPY.gr.
Crus2.gr.
PRM.gr.
Crus1.gr.
SIM.gr.
CTX.SS6b.
CTX.SS4.
CTX.SS6a.
SUB.
POST.
STR.
PRE.
PAL.
Structures

S14 Fig

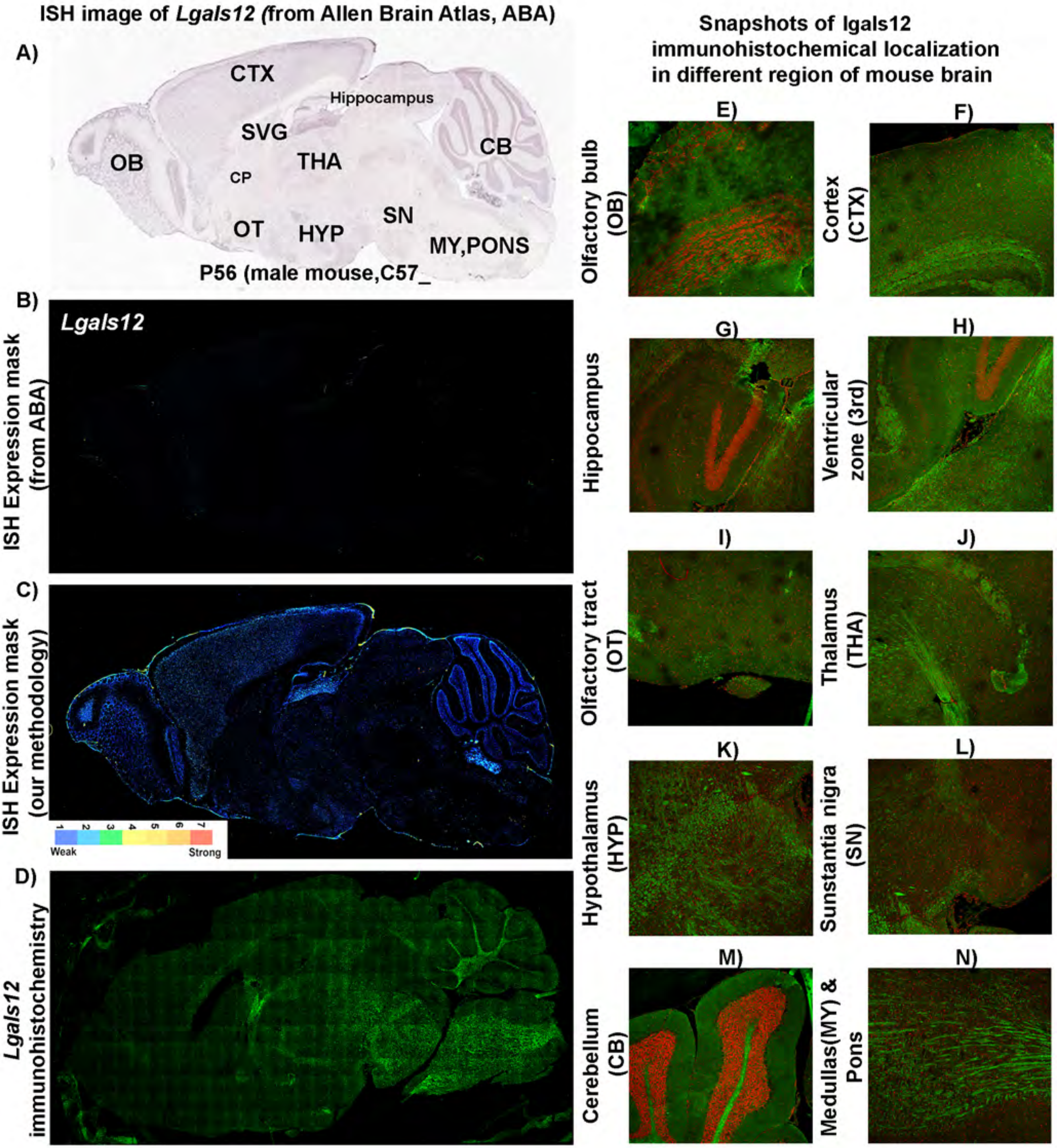

Supplementary Figure 15
