# Supplementary material for: mRNA Transcriptomics of Galectins Unveils Heterogeneous Organization in Mouse and Human Brain
Source: Front Mol Neurosci. 2016 Dec 16;9:139. doi: 10.3389/fnmol.2016.00139 (PMC5159438; doi:10.3389/fnmol.2016.00139)
Supplement: Supplementary file 4 [file Data_Sheet_4.PDF]

Supplementary Figure S16

Supplementary methods and examples for LUT generation; Intensity, Density and Expression factor calculation.

Methodology for Expression Analysis Followed in this Study

mRNA Transcriptomics of Galectins Unveils Heterogeneous Organization in Mouse and Human Brain

Authors: Sebastian John and Rashmi Mishra

Contents

1. How to make a custom pseudo color LUT…………………….. ………… Pages: 2-26

2. How to perform expression Intensity (L) measurements…………….Pages: 27-35

3. How to perform expression Density (D) measurements………………Pages: 36-42

4. How to derive Expression Factor (E)……………………………………………Pages: 43-45

How to make a custom pseudo color LUT

Requirements:

1. Fiji Image Processing Software (just Image J)- 64 bit (freely downloadable)

2. High Resolution Allen Brain Atlas (ABA) ISH images (use Entire Image option in ISH Image download window)

1. Open Fiji -64 bit Image processing software and update it from Help menu dropdown,
2. Restart the software application (always update before use).

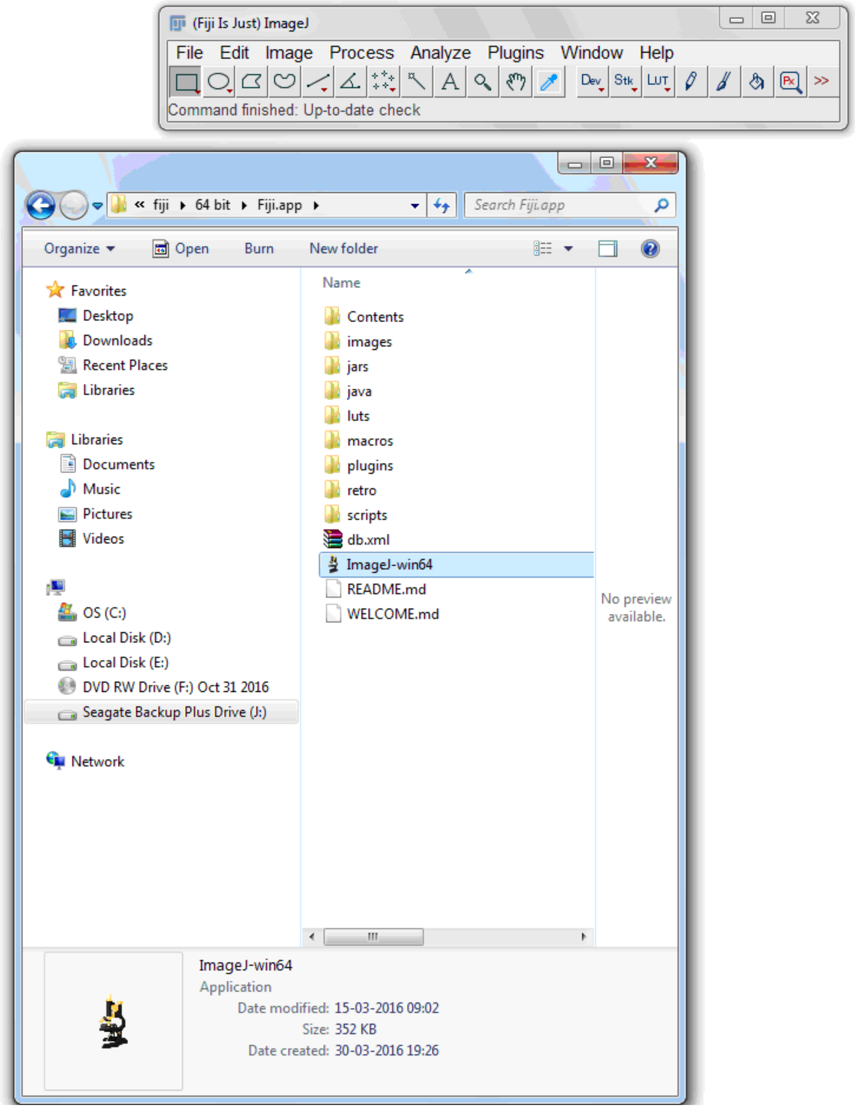

From ‘File’ menu ‘Open’ High Resolution ABA ISH image (Galectin-9 parasagittal plane ISH image is shown here)

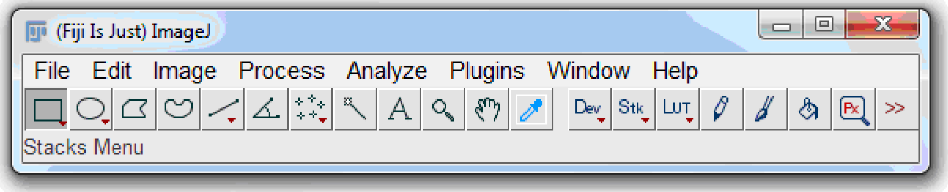

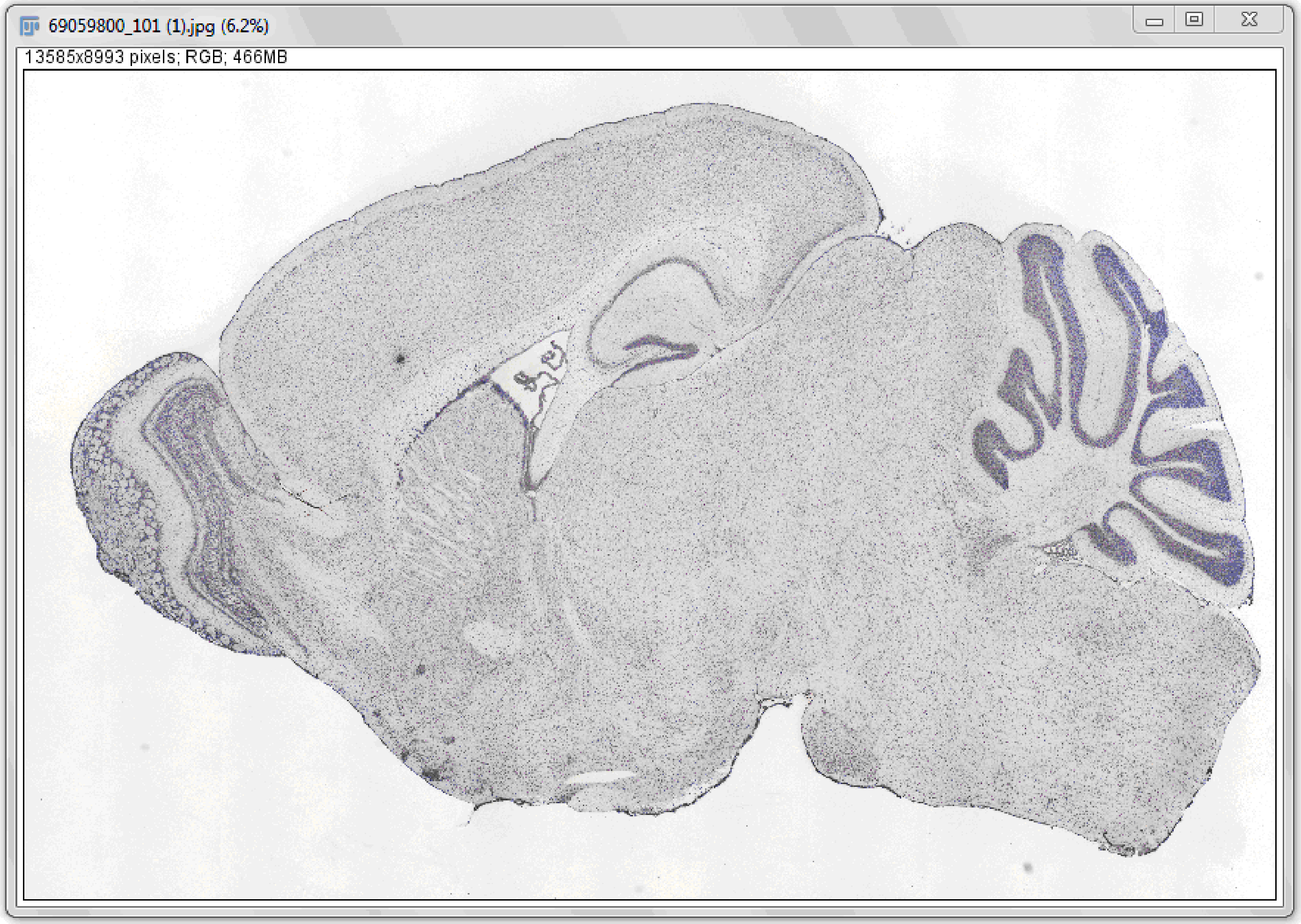

Using Image>Type>8bit command convert the image into gray-scale format

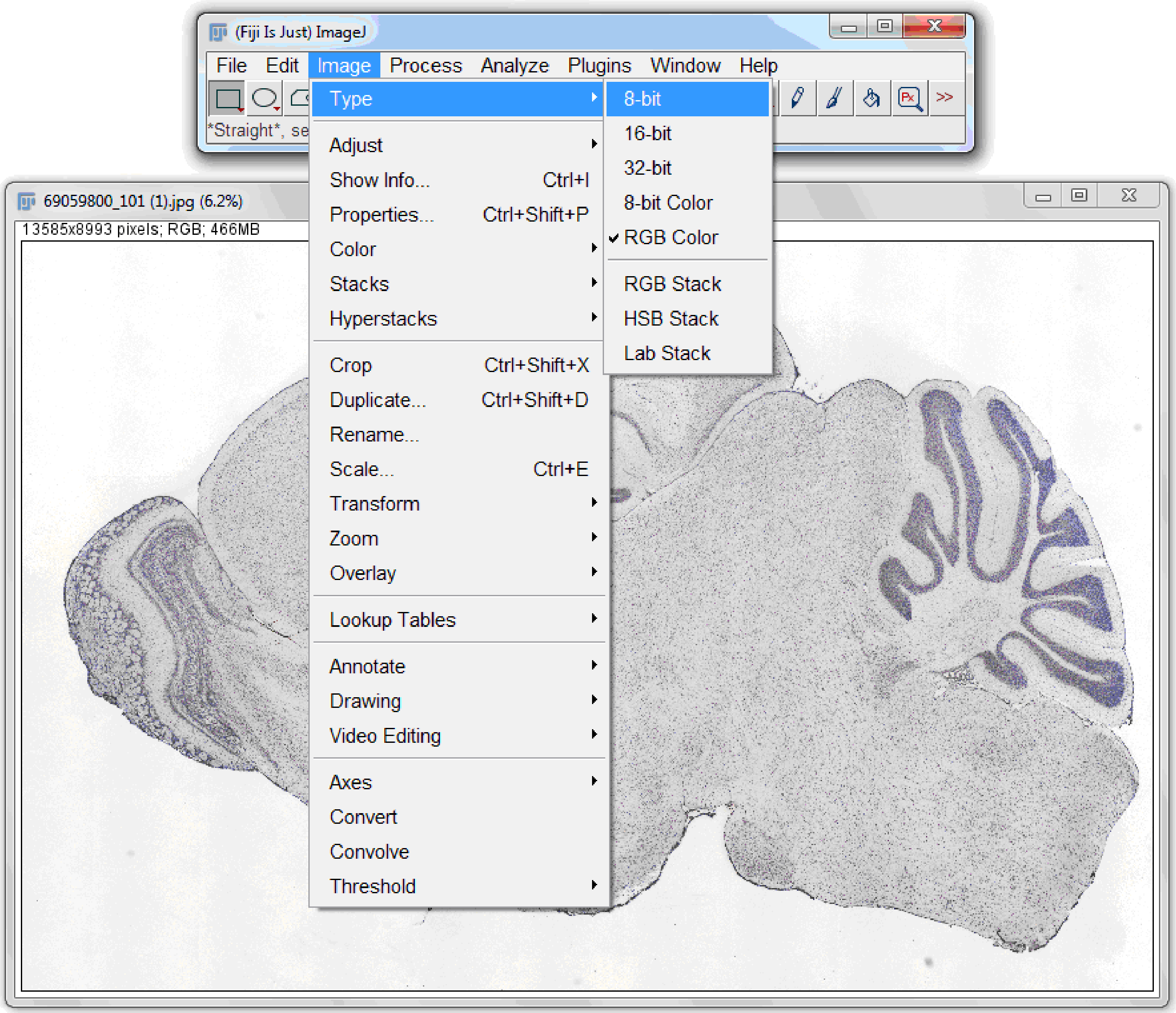

8 bit converted image will look like this:

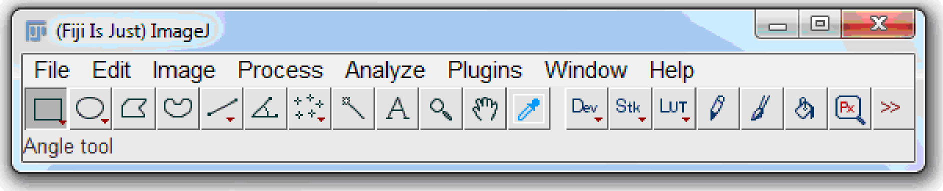

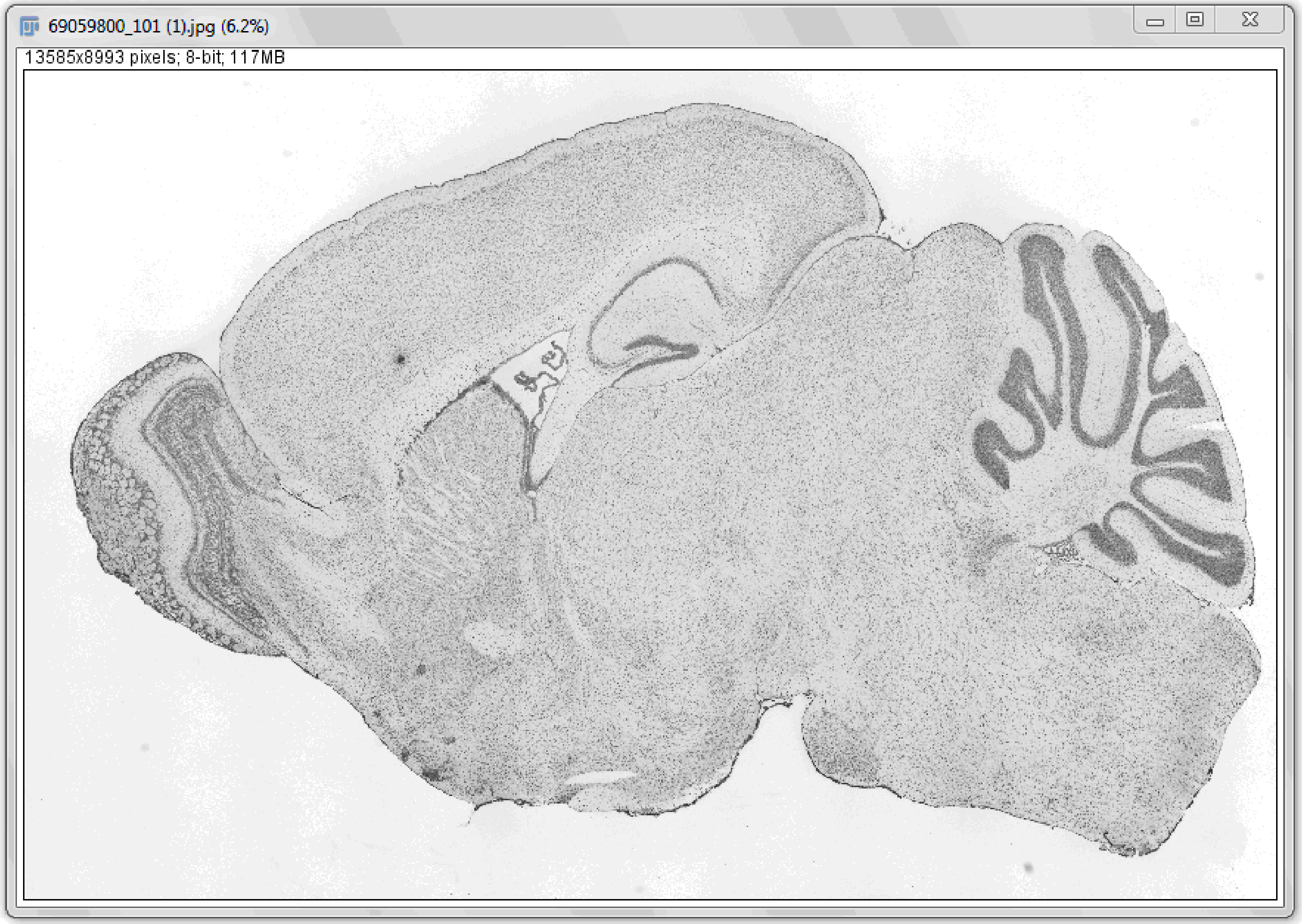

Using Image>Type>Color>Edit LUT option, open the LUT Editor window

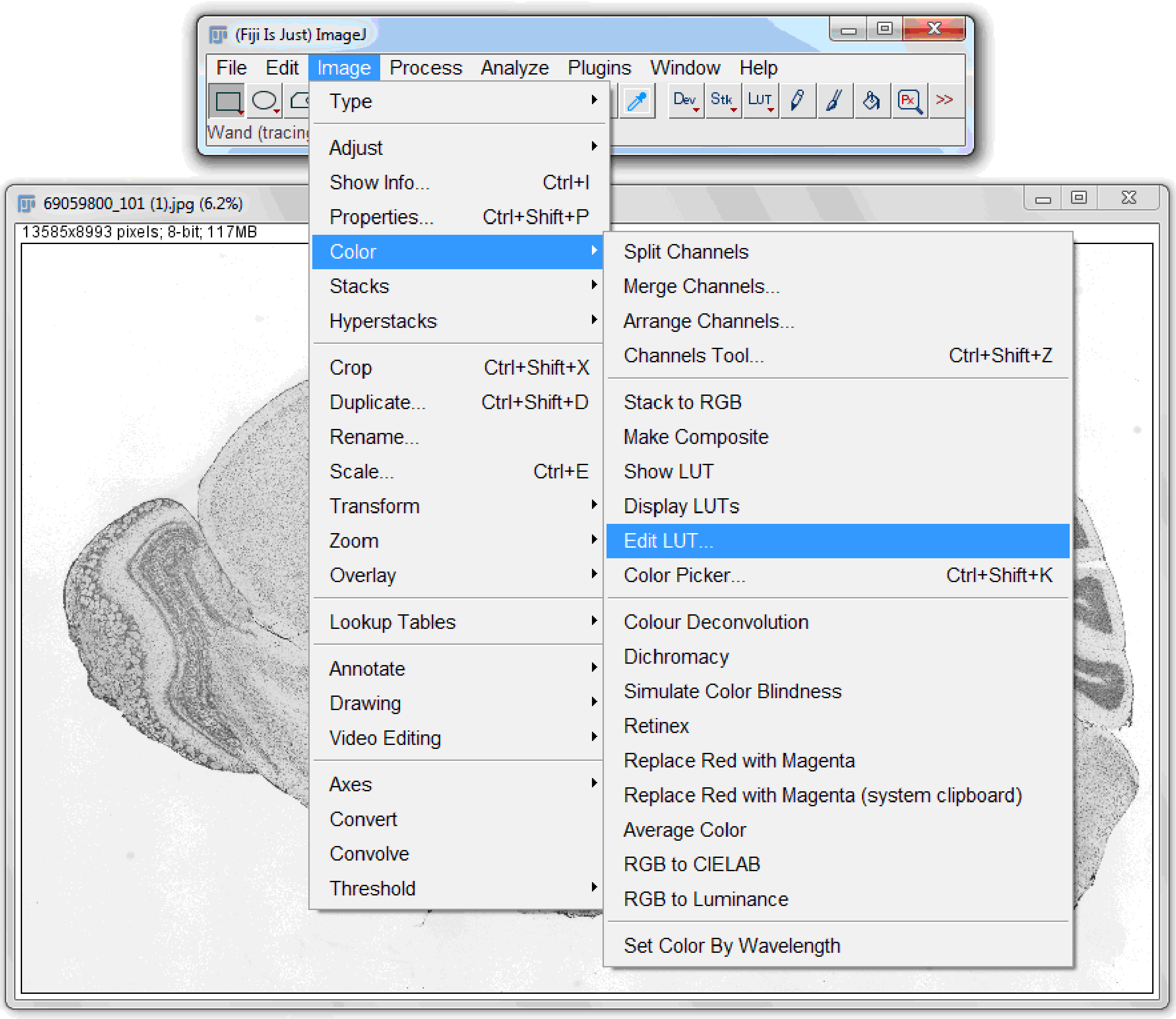

The LUT Editor shows gray scale schema for 256 pixel values in a decreasing order from black (strongest, index position 0) to white (weakest/no signal, index position 255)

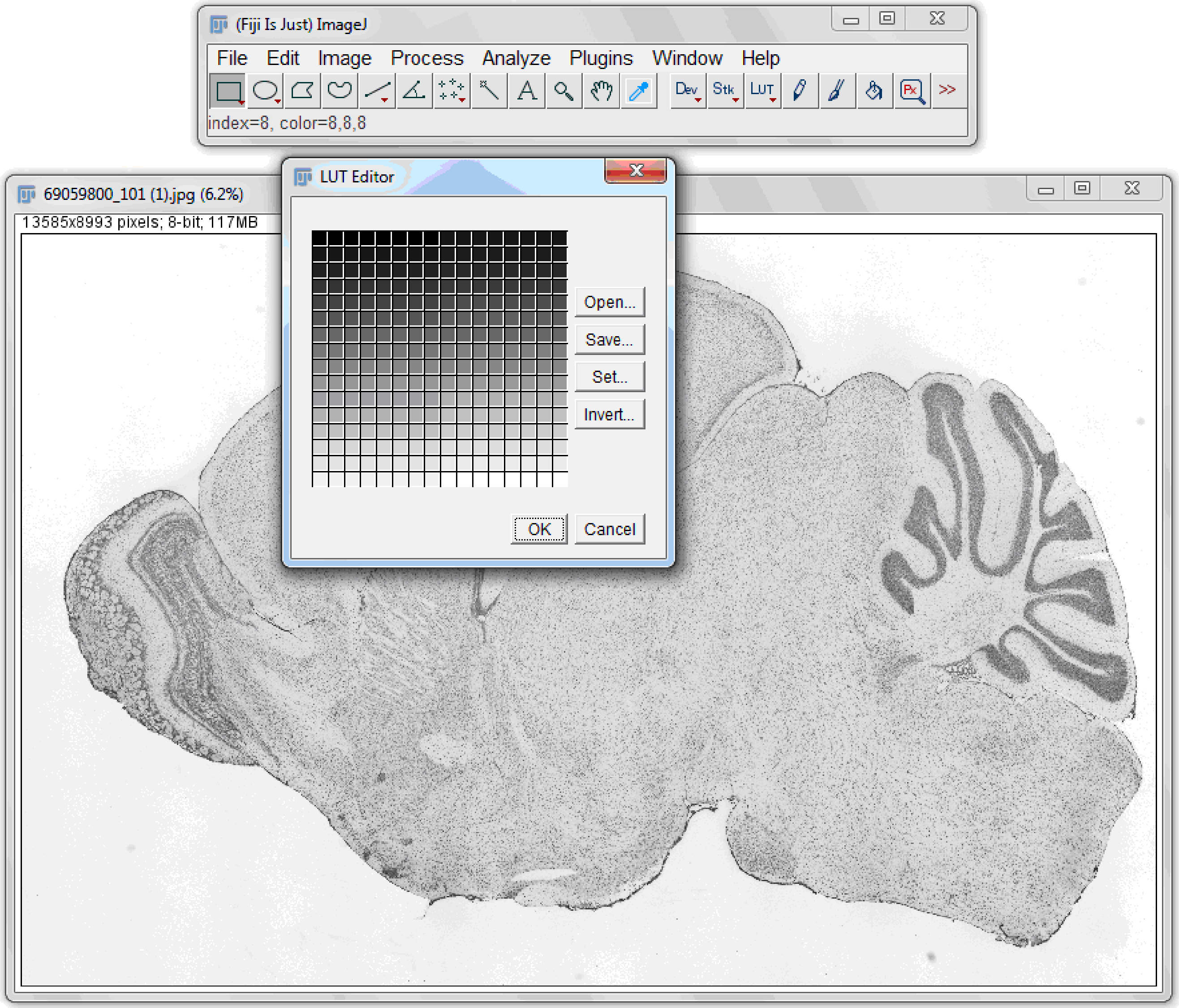

Click on first (0) position in LUT Editor and drag cursor to select first 2 rows (positions 0-31). A color entry pop up window will emerge.

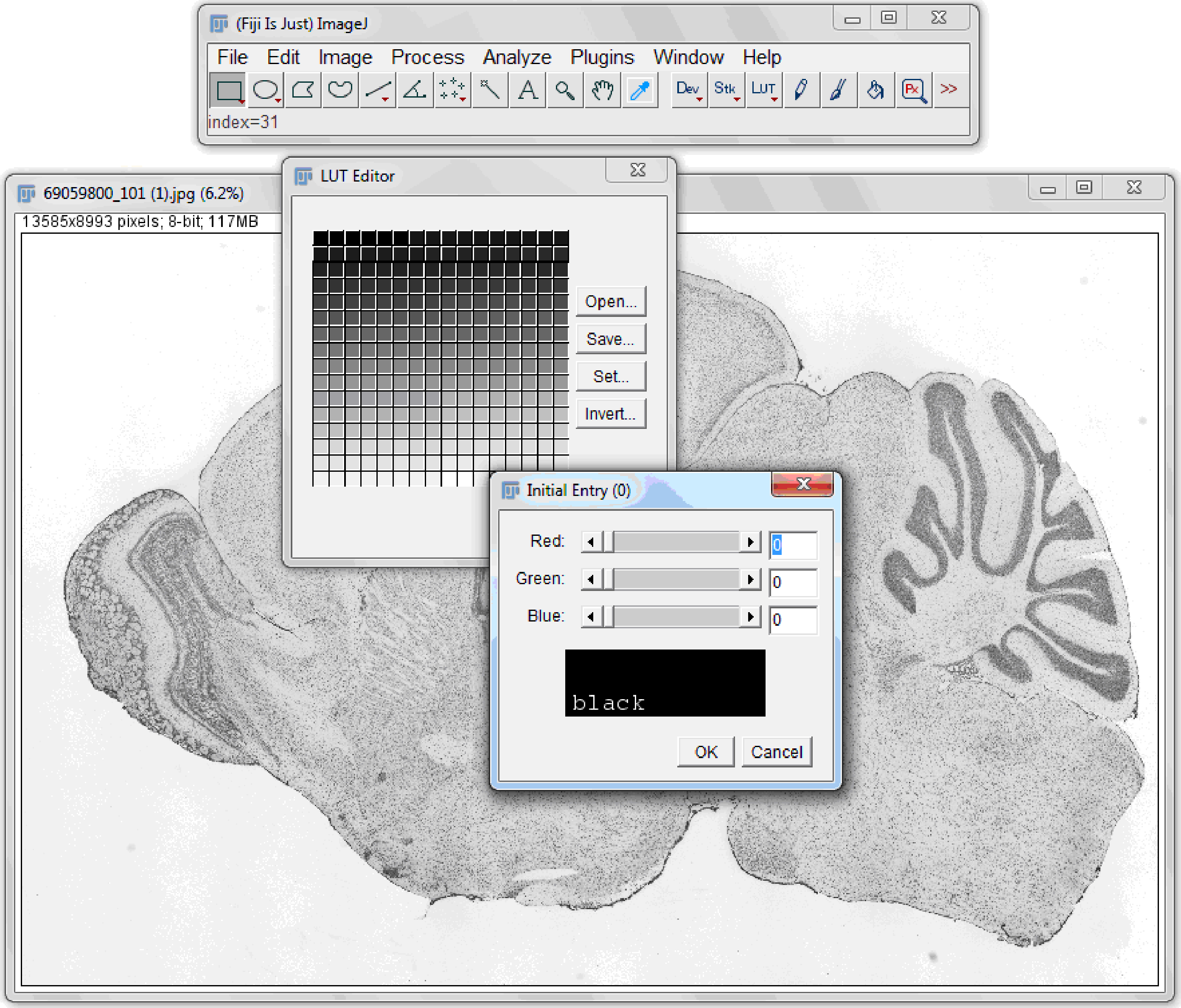

Set RGB readings to be (255, 0, 0) to assign red color to the selected panels for initial entry ‘0’

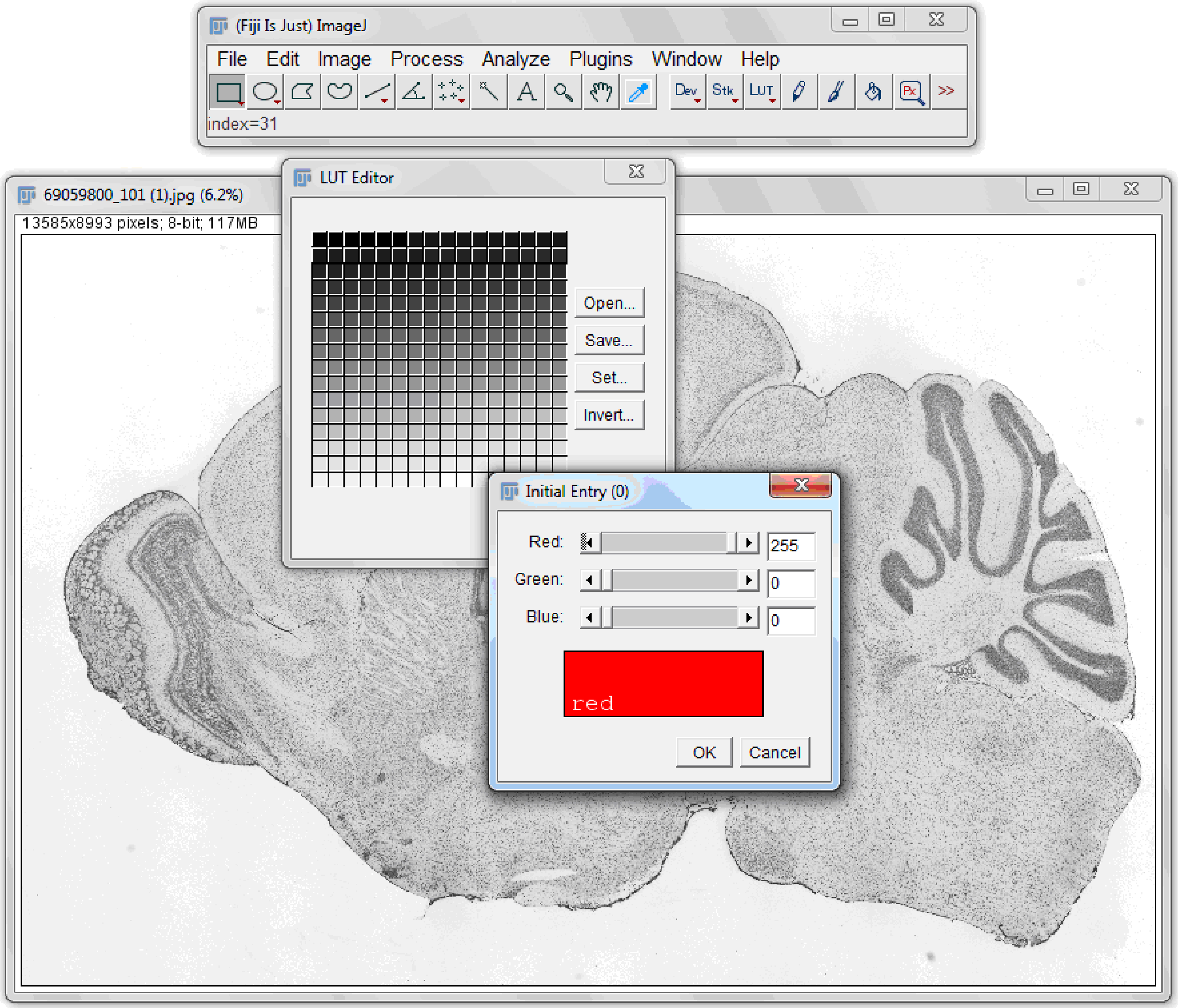

Set RGB readings to be (255, 0, 0) to assign red color to the selected panels for final entry '31'

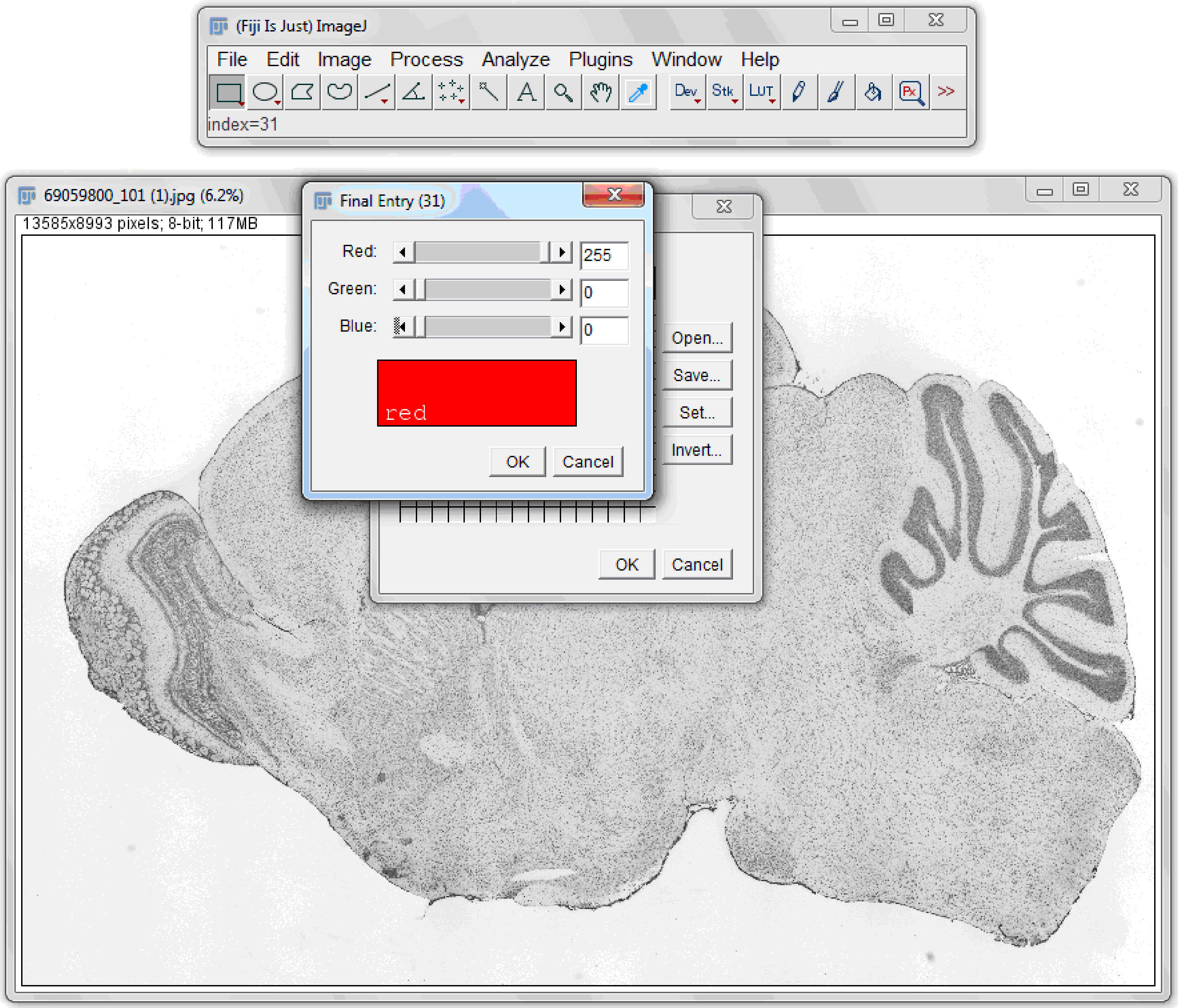

So the first two rows (index position 0-31) are now assigned pseudocolor RED (corresponding to high grey scale values)

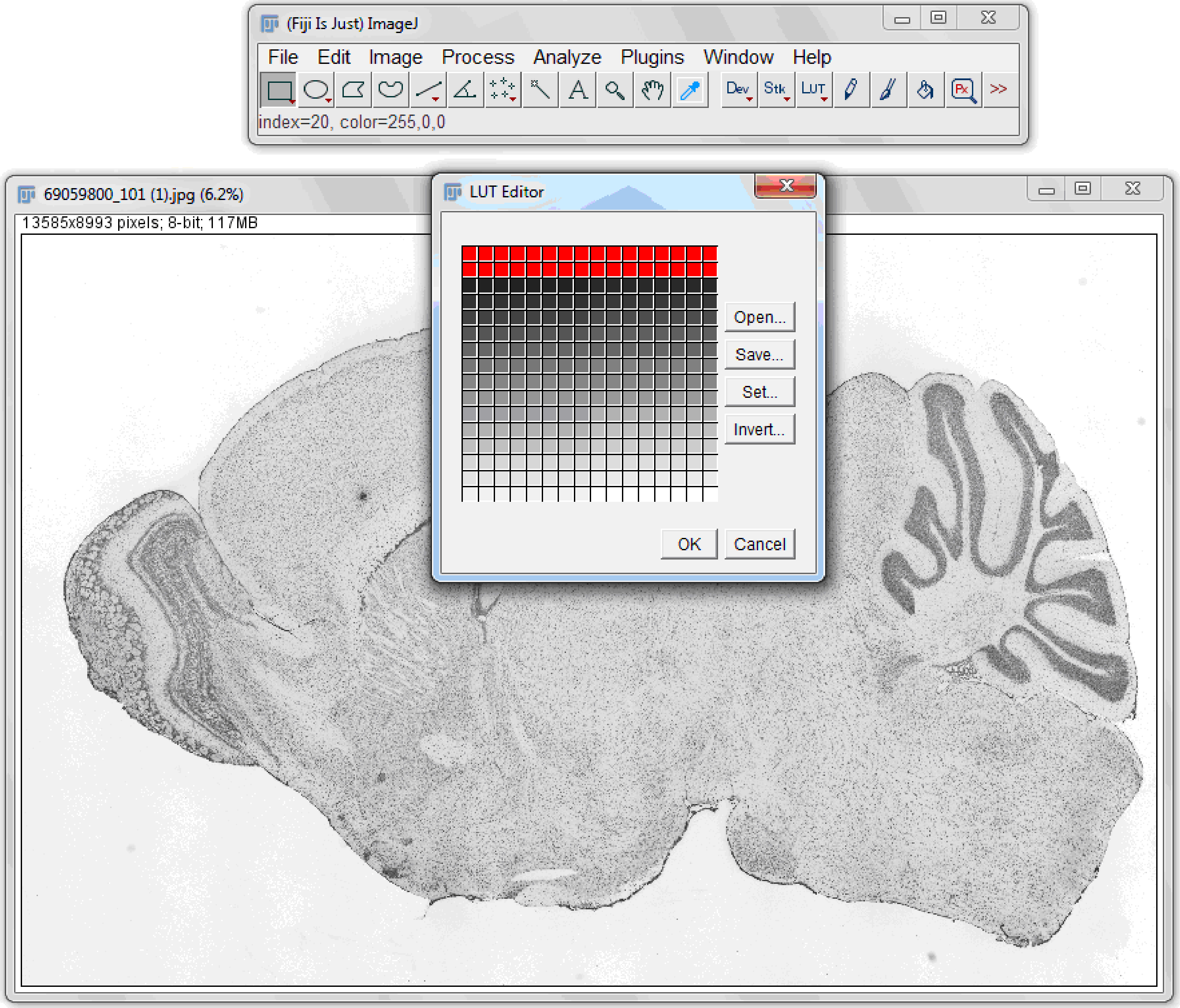

Similar to the previous process next two rows (index position 32-63) are now assigned pseudocolor 'DARK ORANGE' (corresponding to a gray scale values lower than first two panels)

Set RGB readings to be (255,124,0) to assign DARK ORANGE color to the selected panels from initial entry position '32'

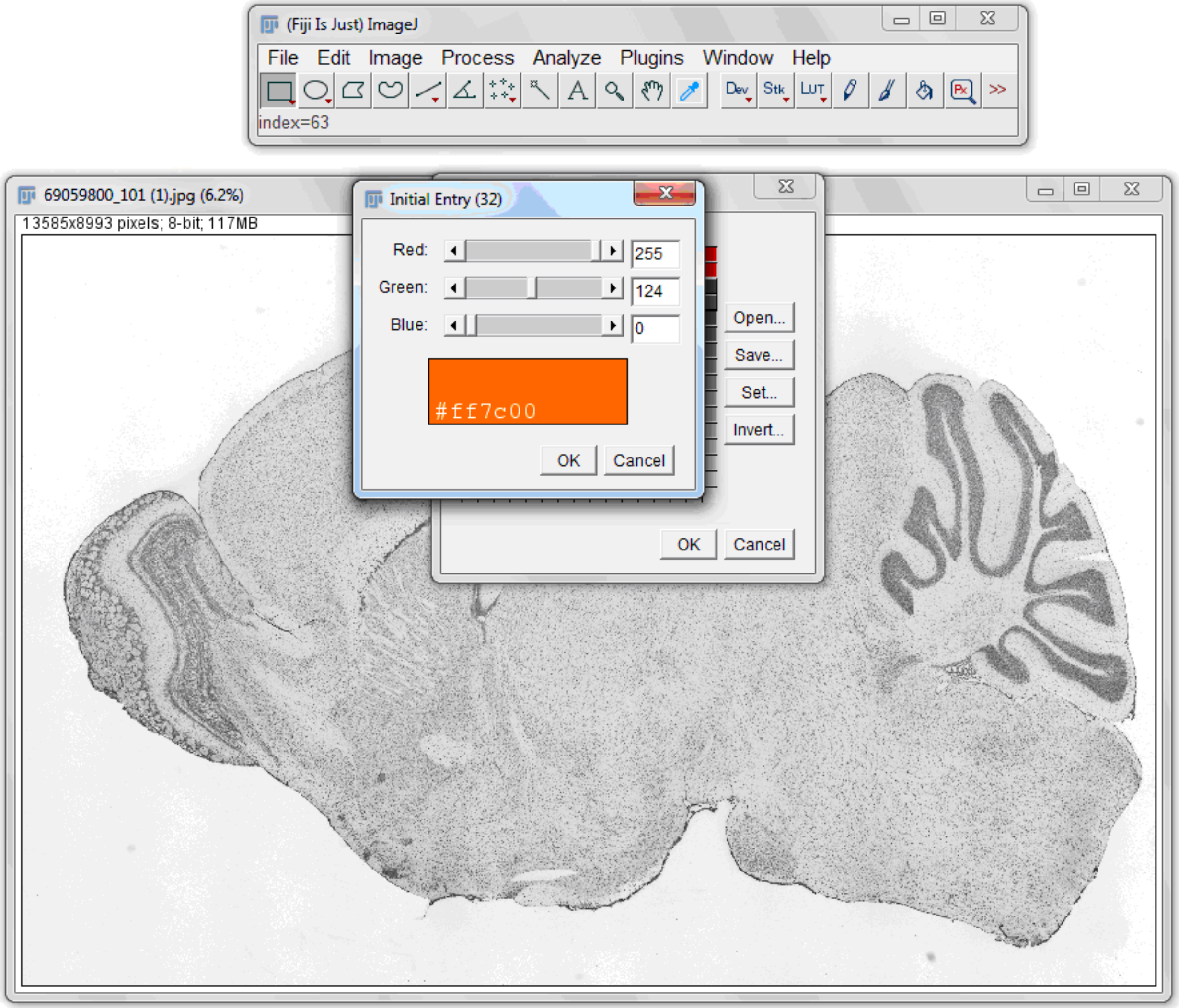

Set RGB readings to be (255,124,0) to assign DARK ORANGE color to the selected panels
In final entry position‘63’

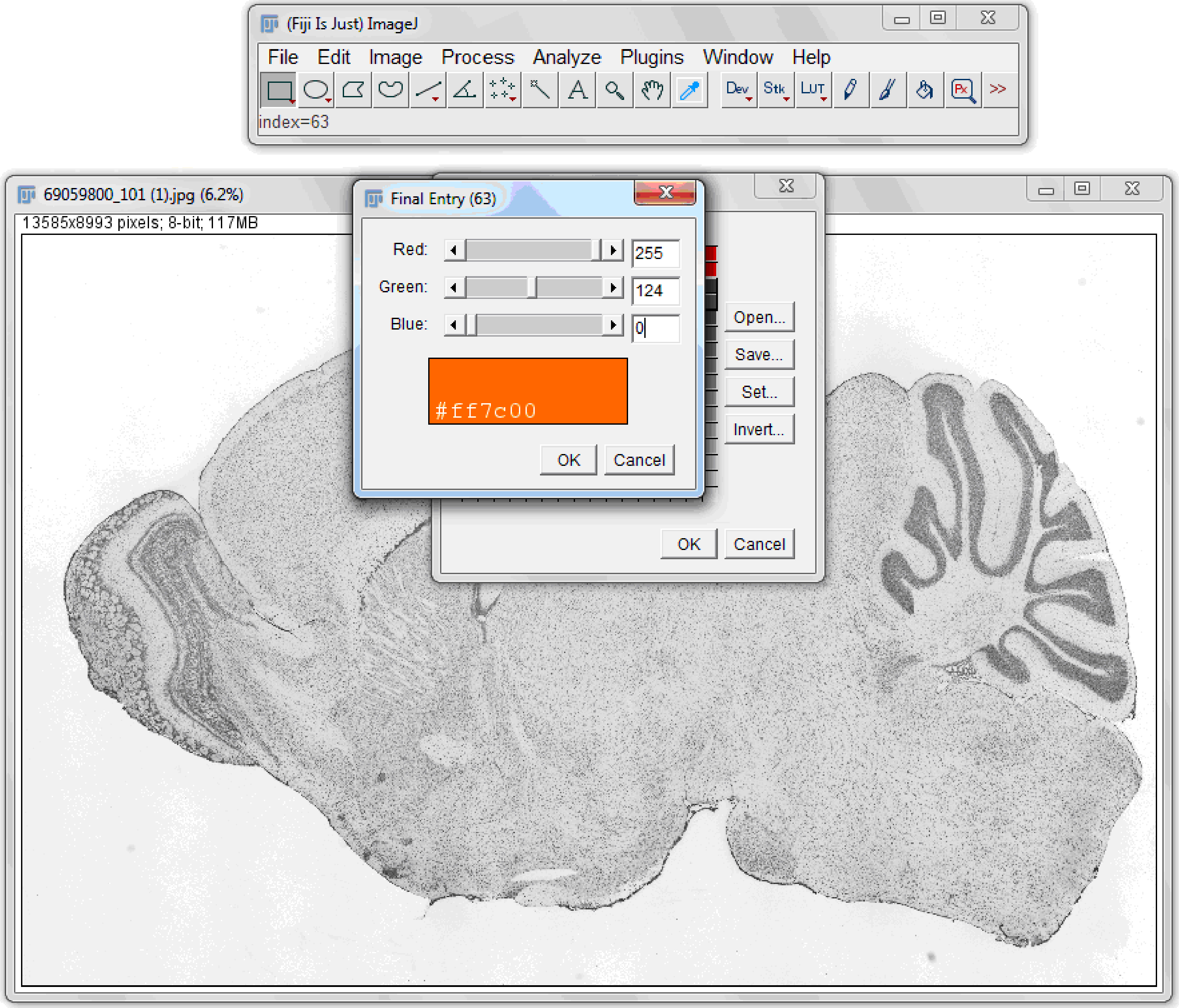

So the panel now looks like this:

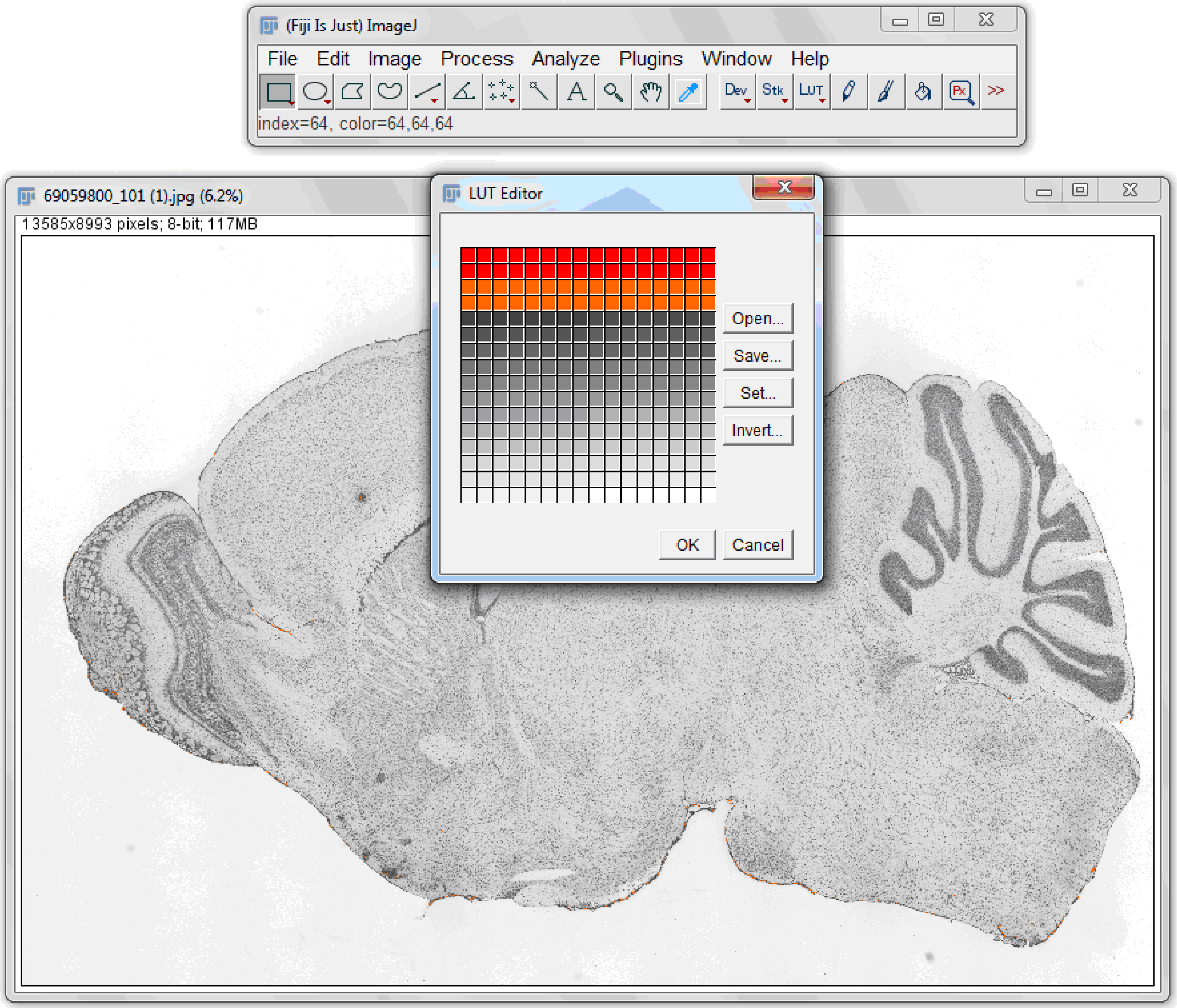

Similarly , proceed to fill the value for orange (255,190,0), yellow (255,255,0), green (0,255,0), See the gray pixels change into pseudocolor pixels in the image accordingly. Remember, pseudocolor does not change the underlying gray scale pixel value.

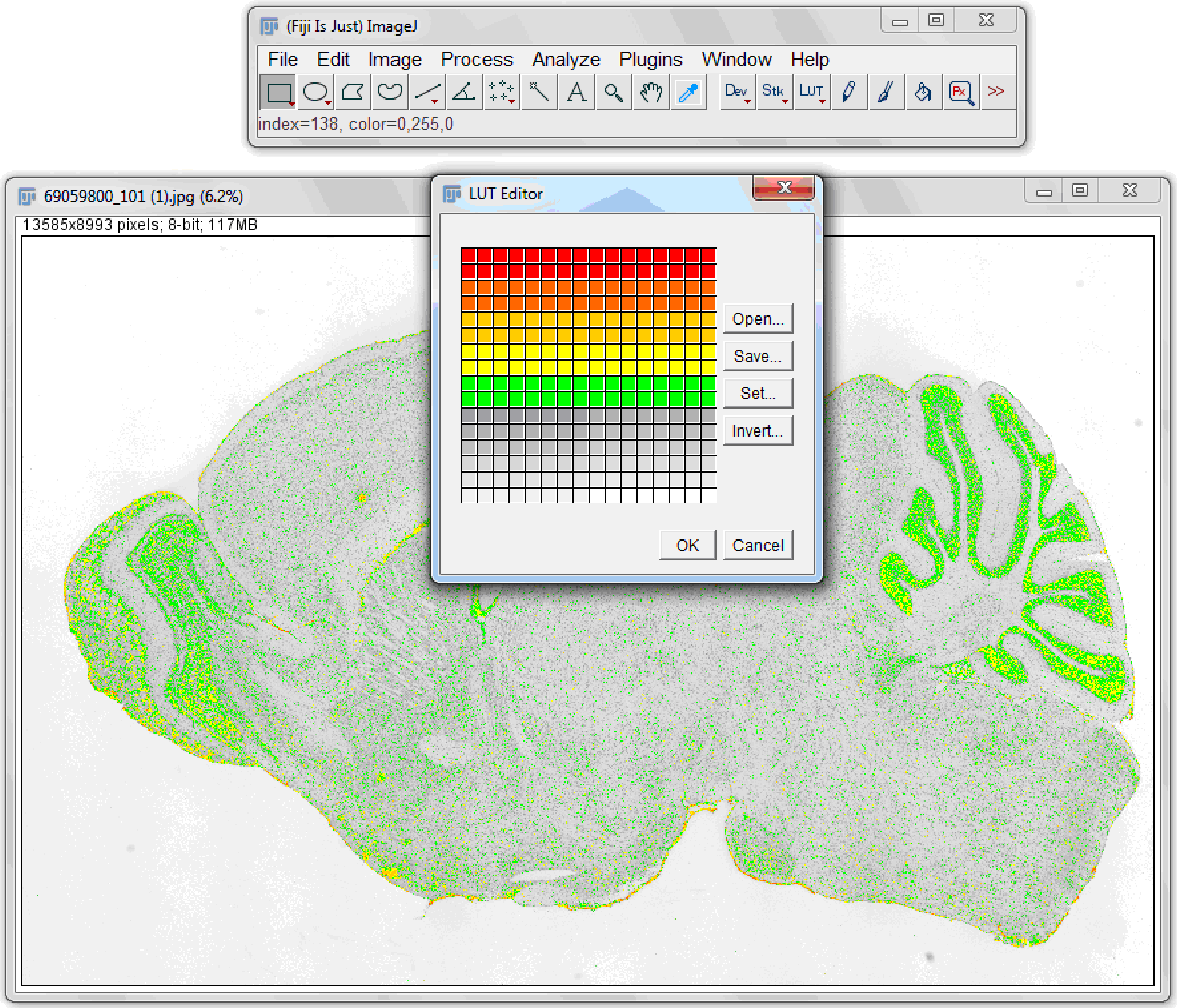

Similarly proceed to fill the value for blue (0,123,255)

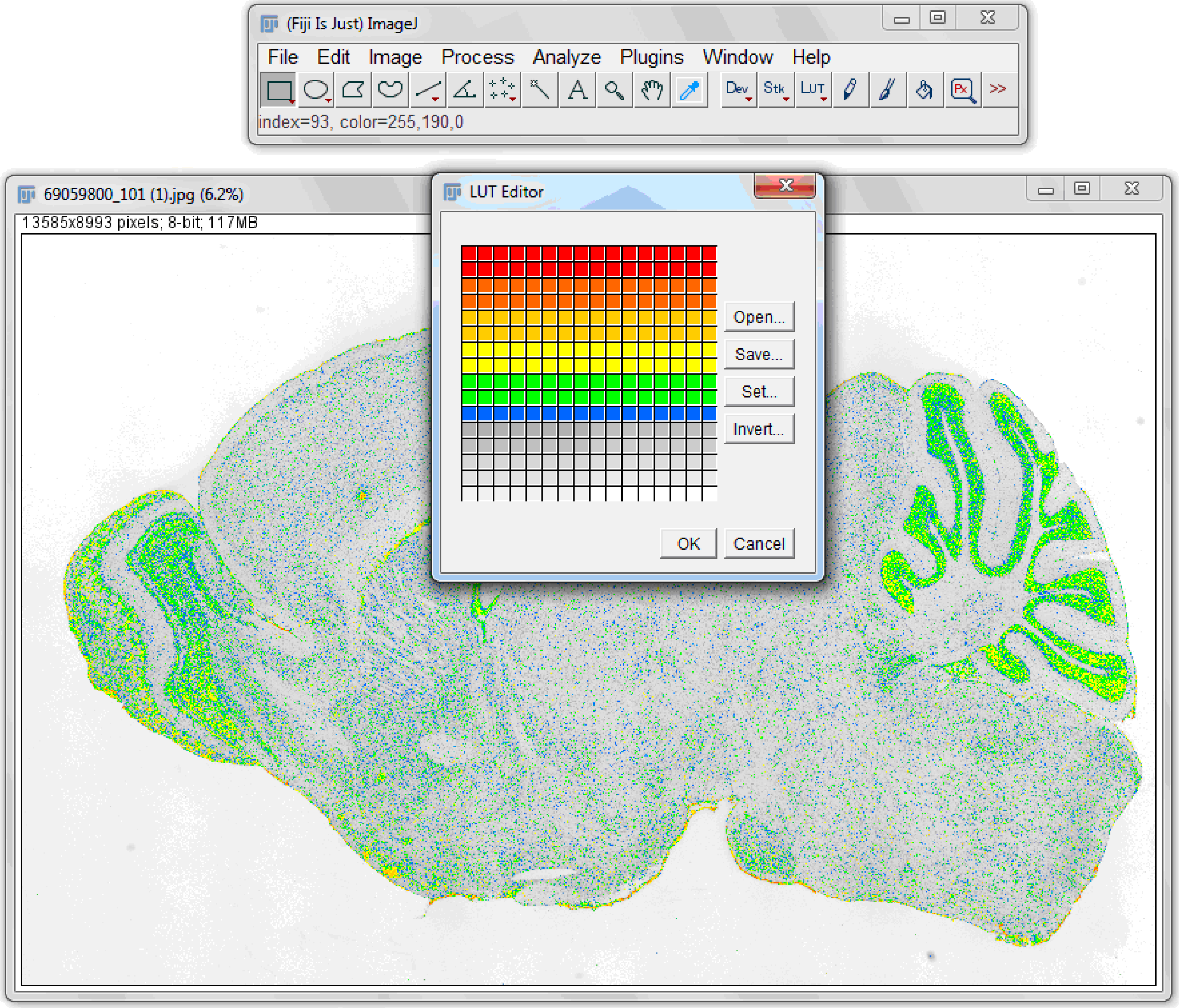

Similarly proceed to fill the value for orange,,
dark blue (0,0,255)

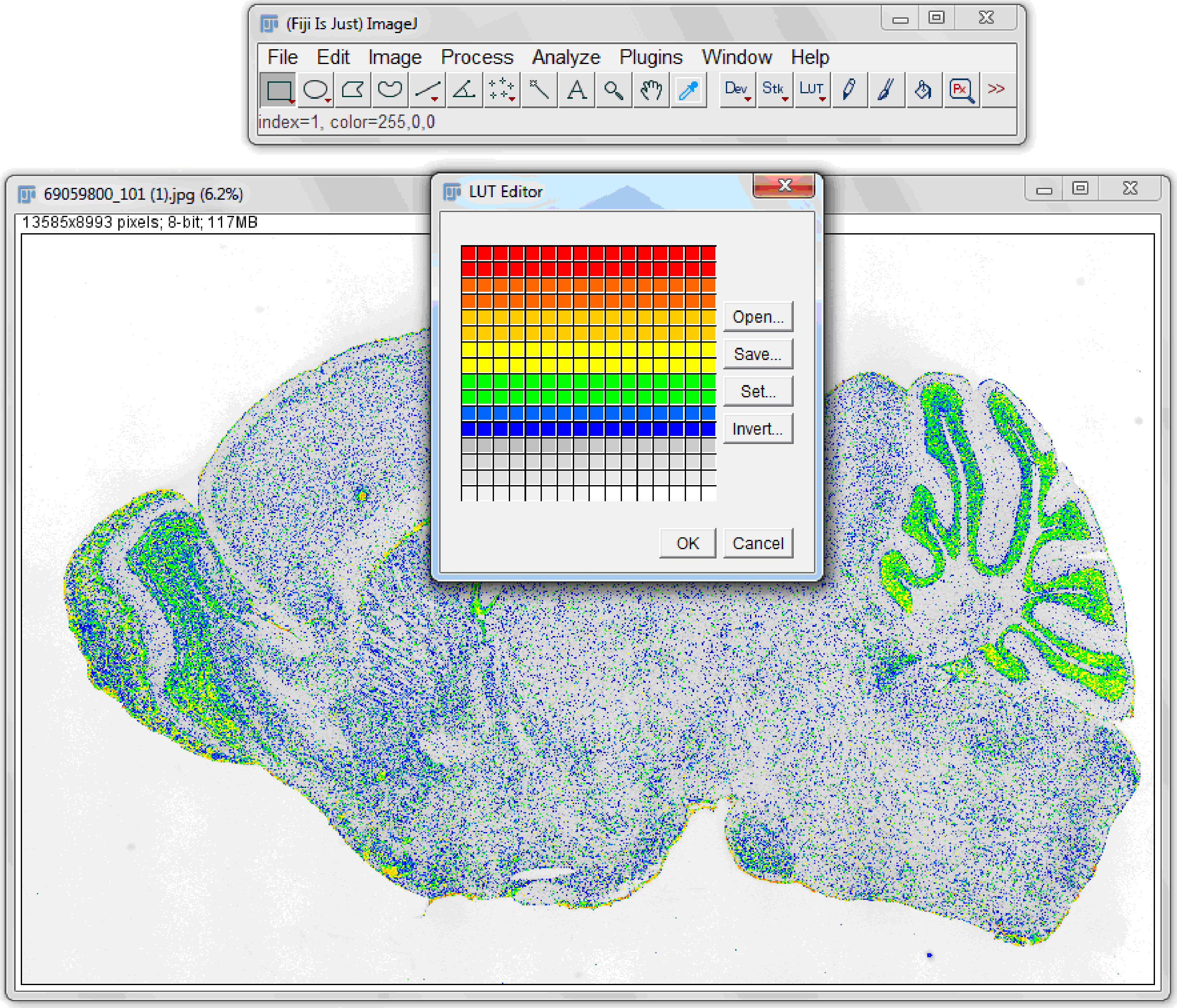

Similarly proceed to fill the value for black (0,0,0). Click OK on LUT Editor.

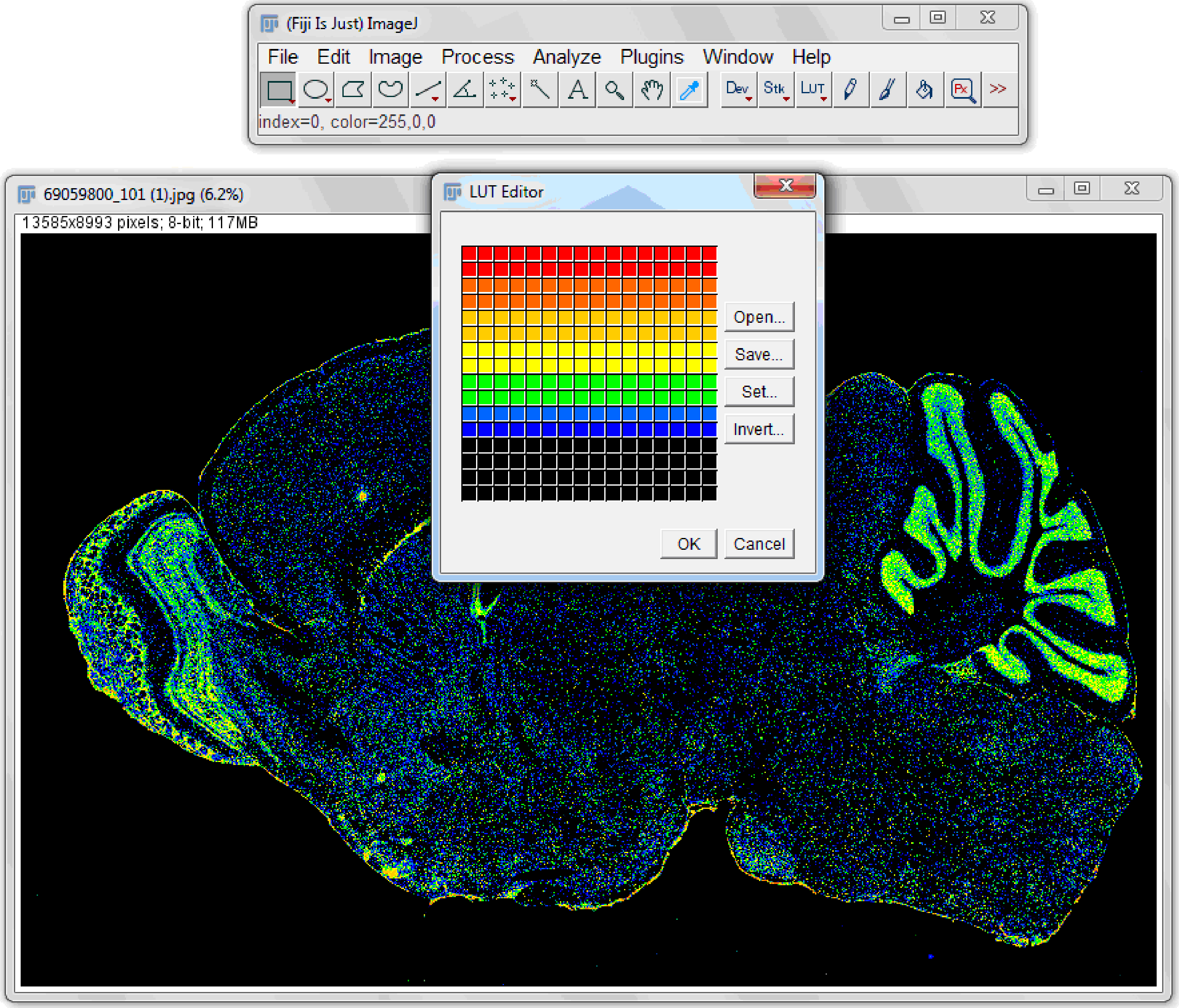

Save the pseudocolor LUT in a folder for reapplication on every ISH image.
Use ‘save’ command on LUT Editor for this

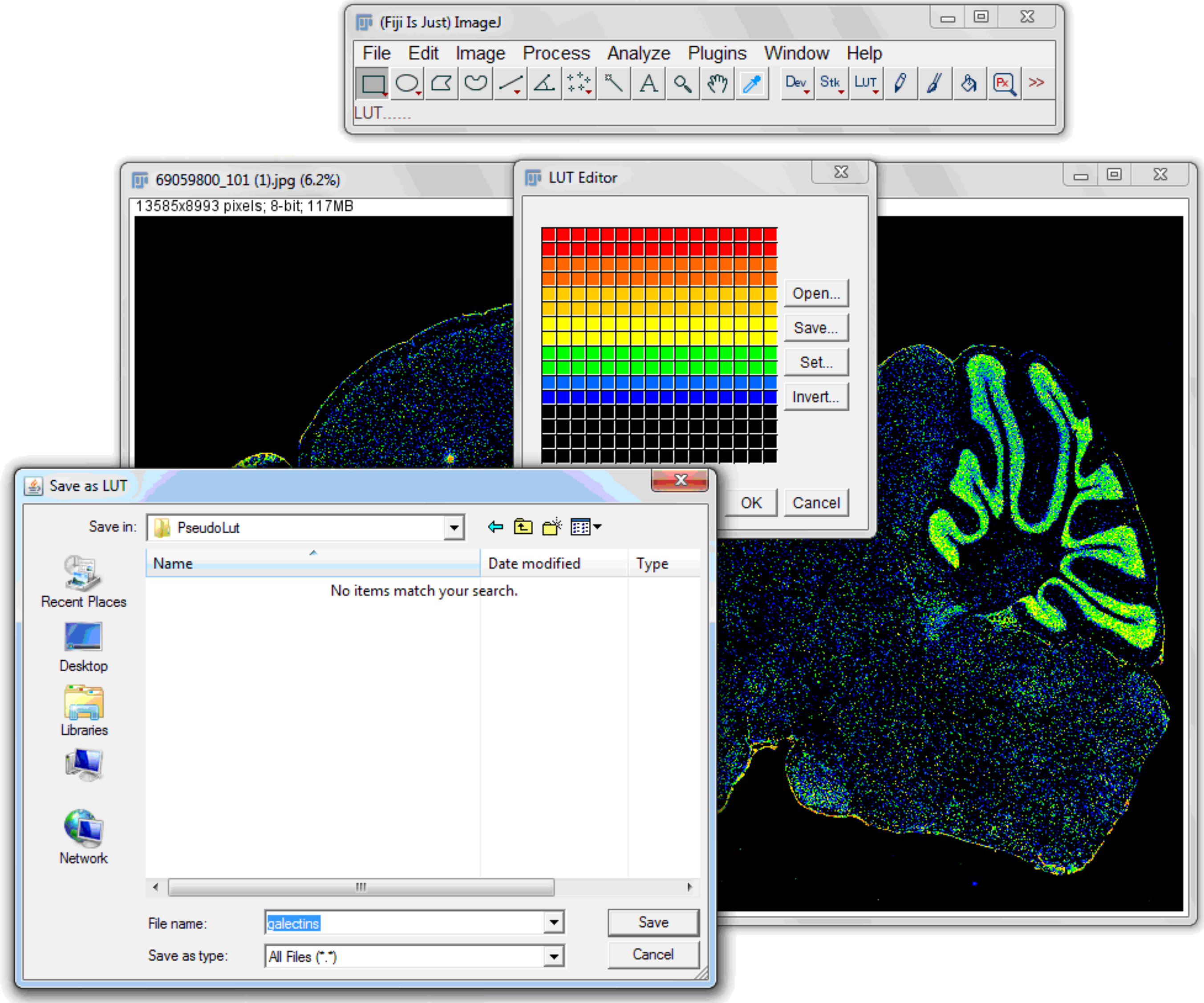

An example of Custom pseudocolor LUT converted ABA ISH image (Lgals9)

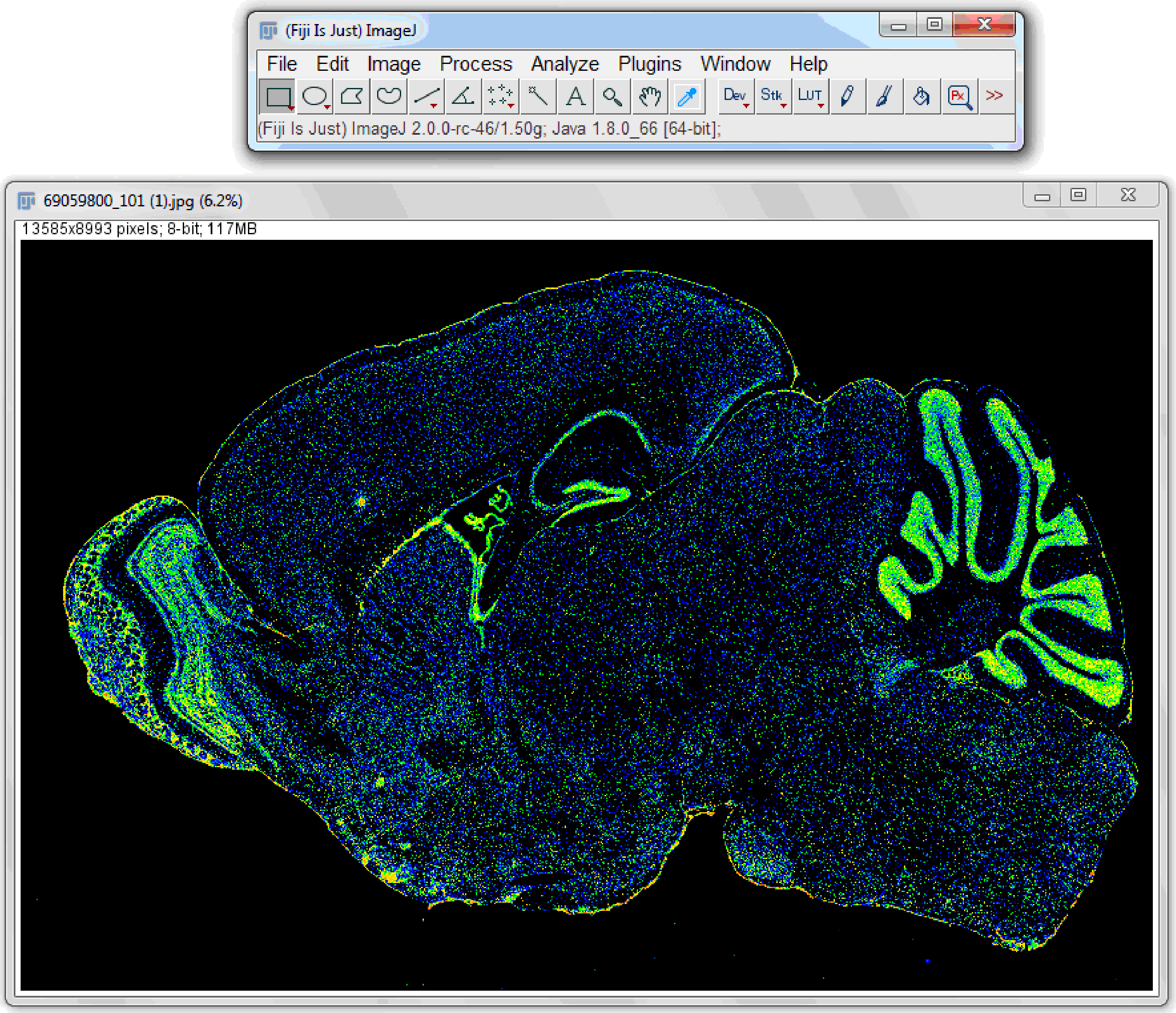

One can visualize pseudocolor calibration bar by using Analyze>Tools>Calibration Bar option

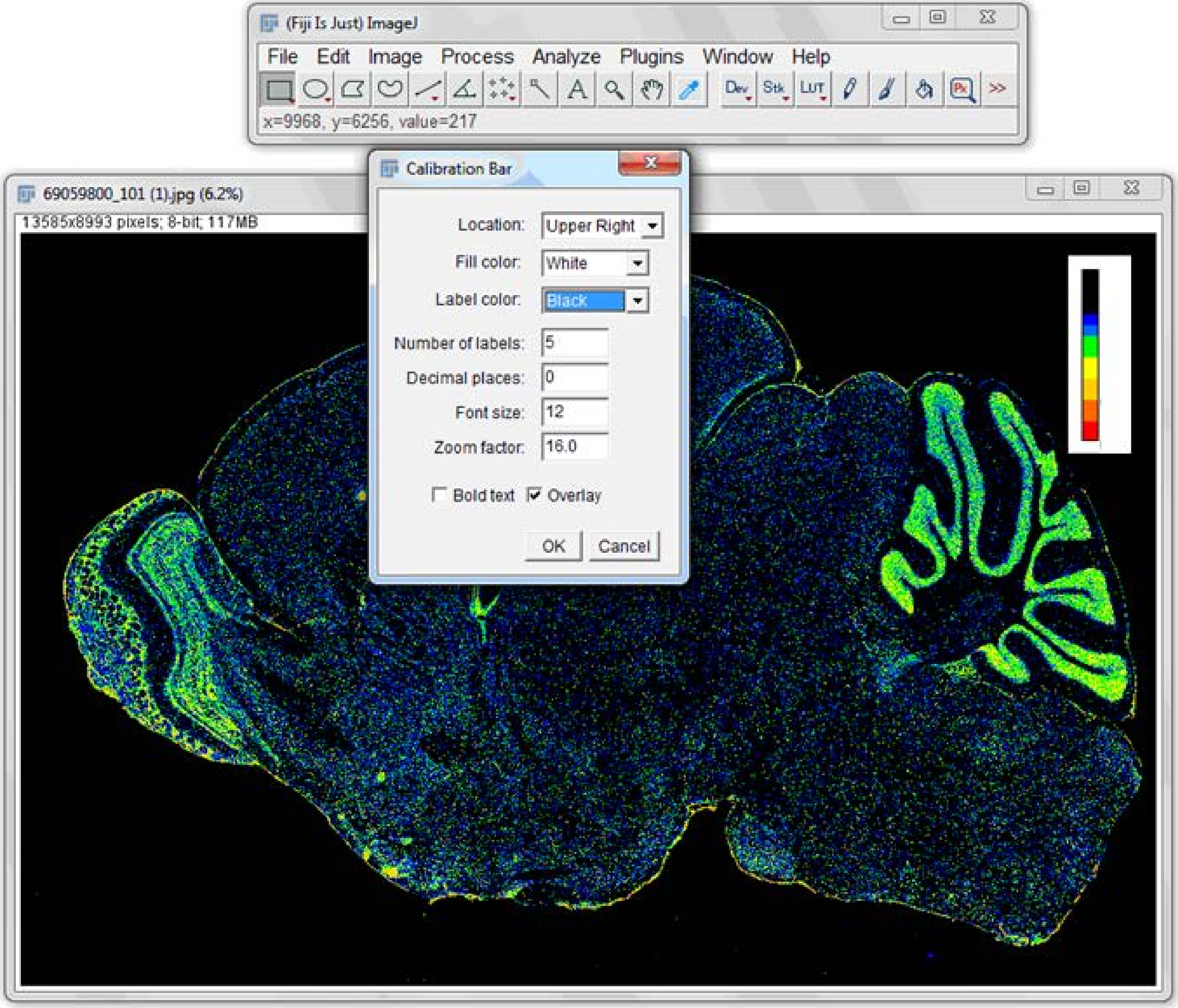

Example of the use of saved pseudocolor LUT on another ISH image (GAPDH)

Open Image, convert to 8 bit. Use Image>Color>LUT Editor>open option to open the saved pseudocolor LUT file

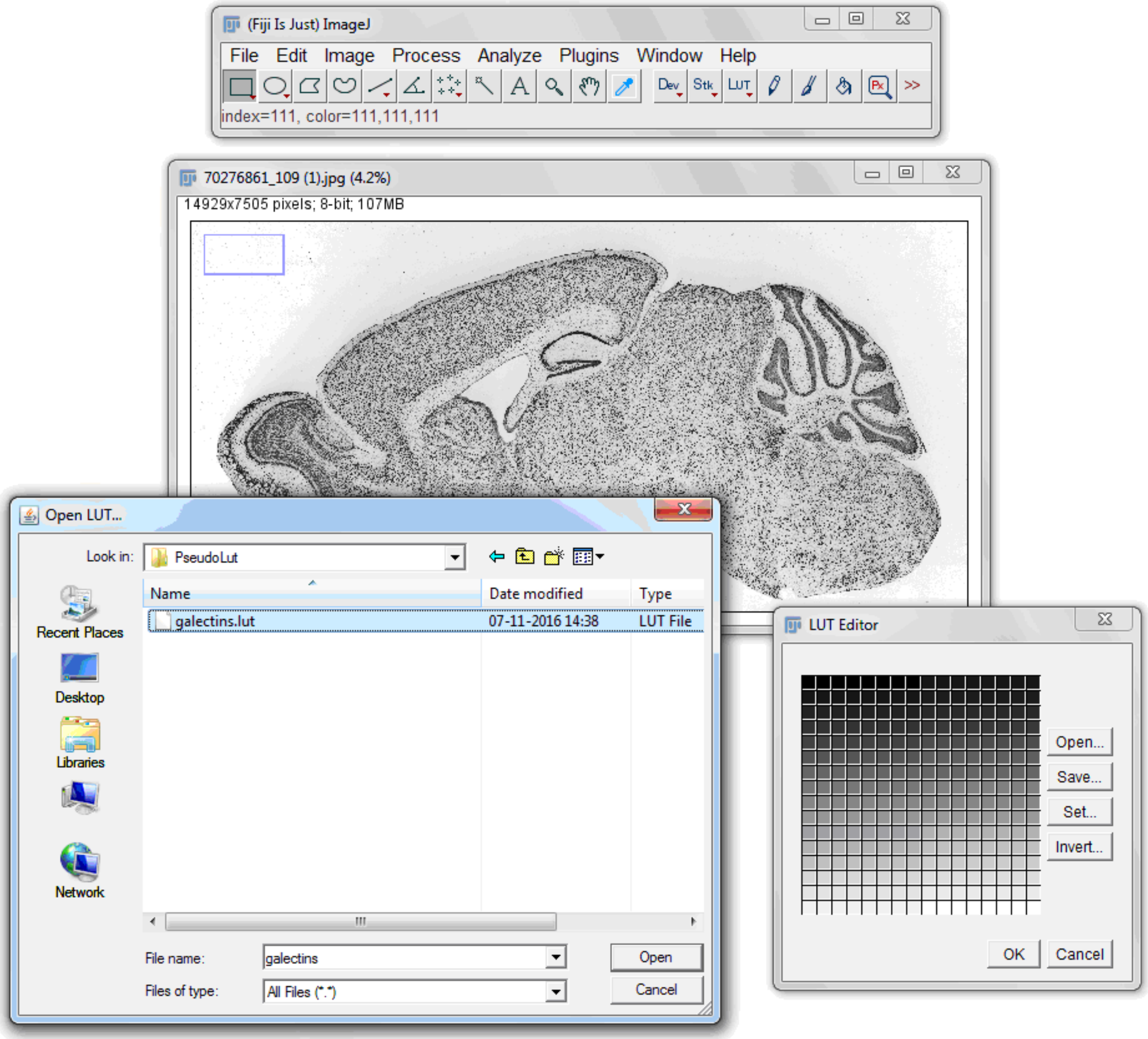

Click OK on LUT Editor displaying custom LUT. Example of use of pseudocolor LUT converted ISH image (GAPDH)

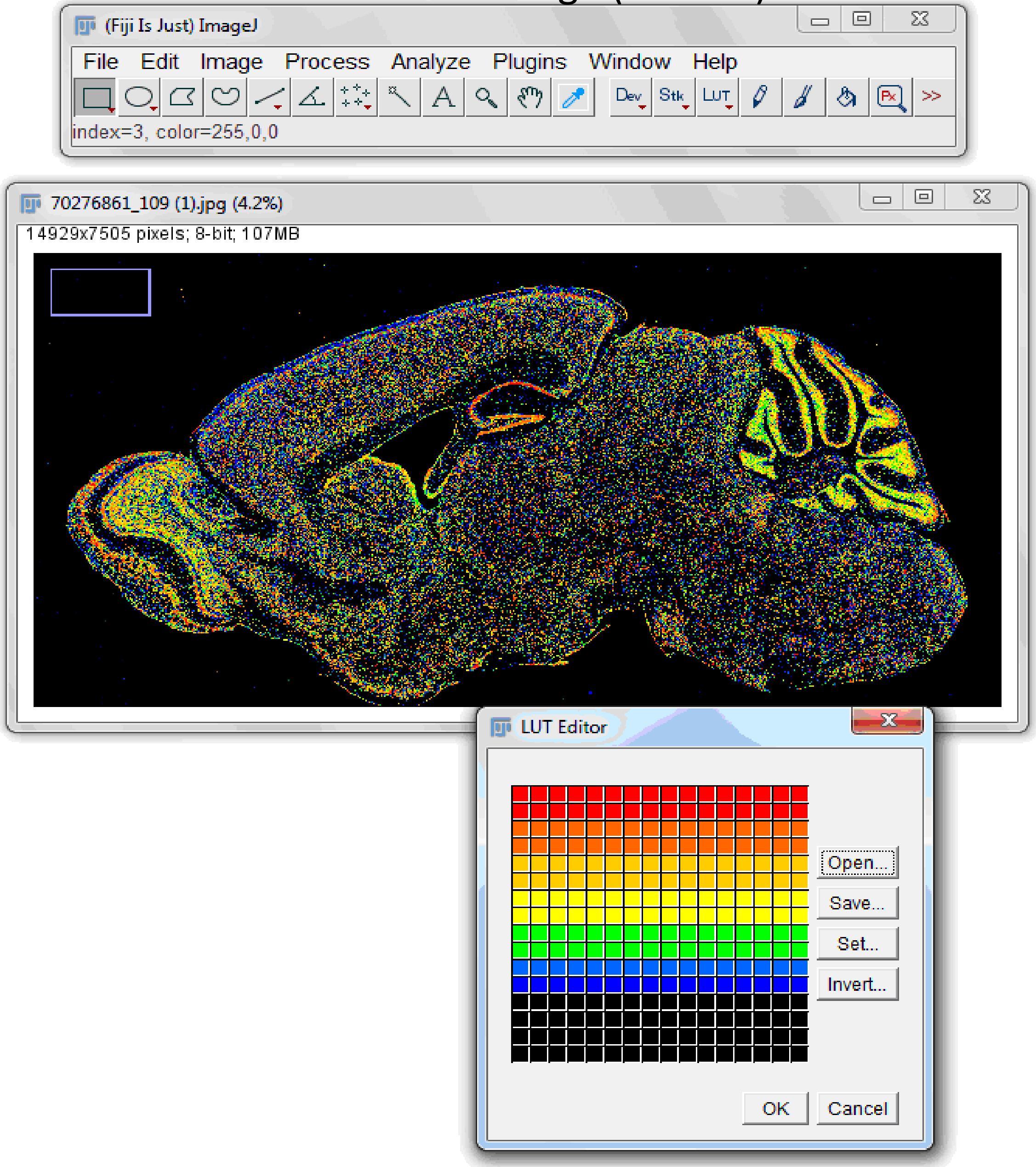

Expression Intensity (L) Scaling from 7-0 (highest to no signal values)

A series of decreasing integers from 7 to 0 were then assigned to selected color codes in order to generate a calibration scale. that is

(i) '*very very high*'/very strong expression (color: red, index position:0-31, pixel intensity scale=7),

(ii) '*very high*'/strong expression (color: dark orange, index position:32-63, pixel intensity scale=6);

(iii) '*high*'/above moderate expression (color: orange, index position:64-95, pixel intensity scale=5;

(iv) **'***medium'***/**moderate expression (color: yellow, index position:96-127, pixel intensity scale=4);

(v) '*low*'/below moderate expression (color: green, index position:128-159, pixel intensity scale=3);

(vi) *'very low'/weak expression* (color: cyan, index position:160-175, pixel intensity scale=2);

(vii) detectable but *'very very low' expression* (color: blue, index position:176-191, pixel intensity scale=1);

(viii) *'no expression/no signal'* (color: black; index position:192-255, pixel intensity scale=0).

<u>This pseudocolor LUT was saved as a Fiji plug-in and all ISH 8 bit converted images were edited using the same LUT (in LUT Editor)</u> to visualize differential expression signal for each gene. Note that equal 'intensity' index positions were assigned for scales from '*very high*' expression (red, intensity scale=7) to '*low*' (green, intensity scale=3) in LUT Editor, that is a total of 32 positions. The next 32 index positions below 'low/green' were equally divided into 16 positions each to distinguish weak signals into '*very low*' and '*very very low* 'pixel intensities. Last 64 index positions (from 192-255) were assigned for '*no signal*' as these positions corresponded to background intensities 8 bit grey scale format.

How to perform expression Intensity (L) measurements

Requirements:

- High Resolution ABA ISH Images
- Corresponding or near synchronous Nissl’s Image to trace ROI
- Corresponding ABA Reference Atlas to map relevant ROI, if needed by individual

ISH full brain images were first cropped for defined subregions (if required ABA reference atlas can be used to identify relevant regions. Use of Image>Transform>Rotate tool for image Alignment can be done).

The region matched Nissl's image was aligned with ISH image and ROI (region of interest) was selected, in this case, for Layer 5 of Cortex (CTBX L5). ROI tool is in Analyze menu.
Register ROI in ROI manager for superimposition in ISH image (in this case ISH image is of Lgals8)

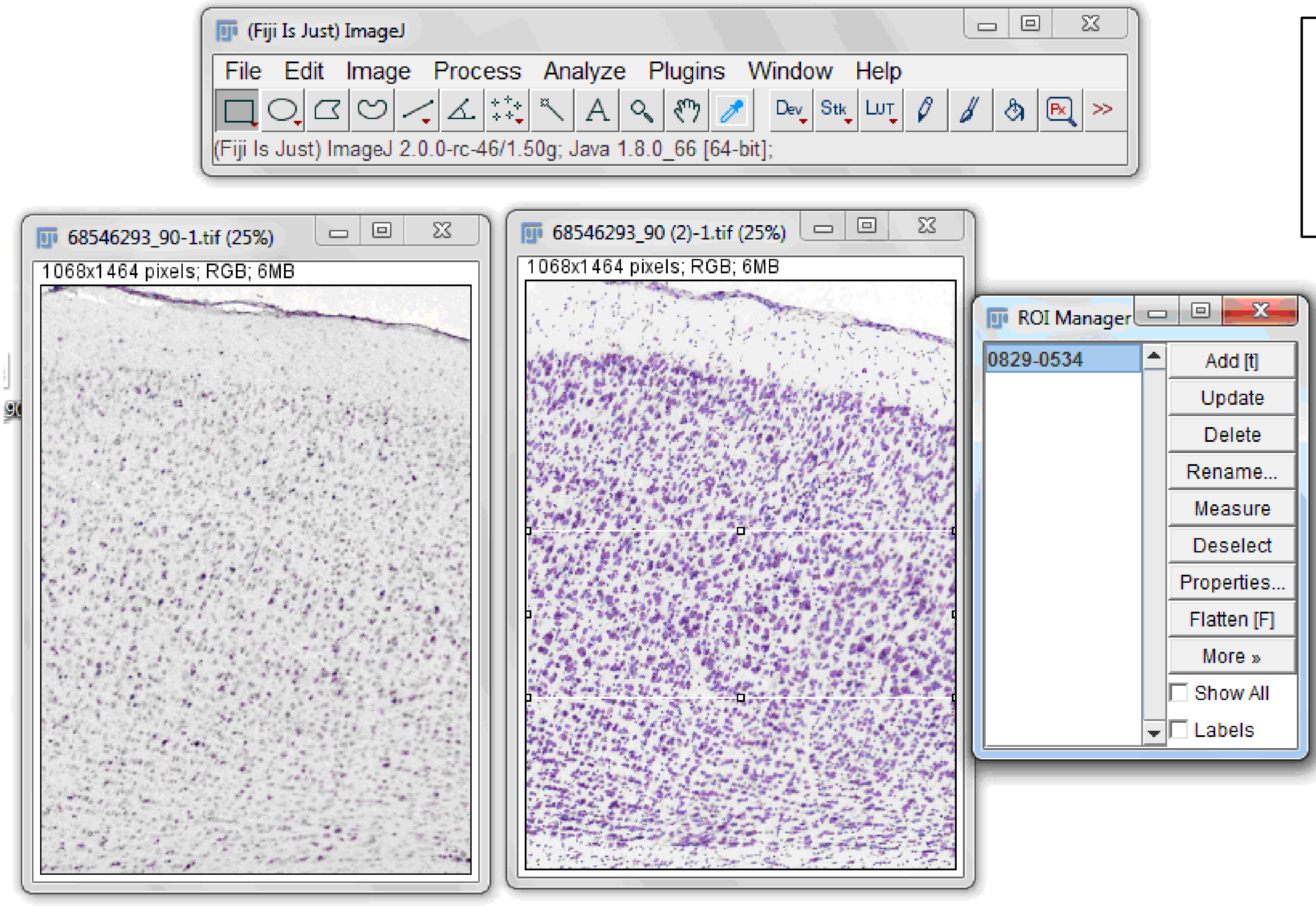

In several cases, Free hand tool may be required for ROI tracing of more uneven

Convert ISH image to 8 bit and threshold it by using Image>Adjust>Threshold option

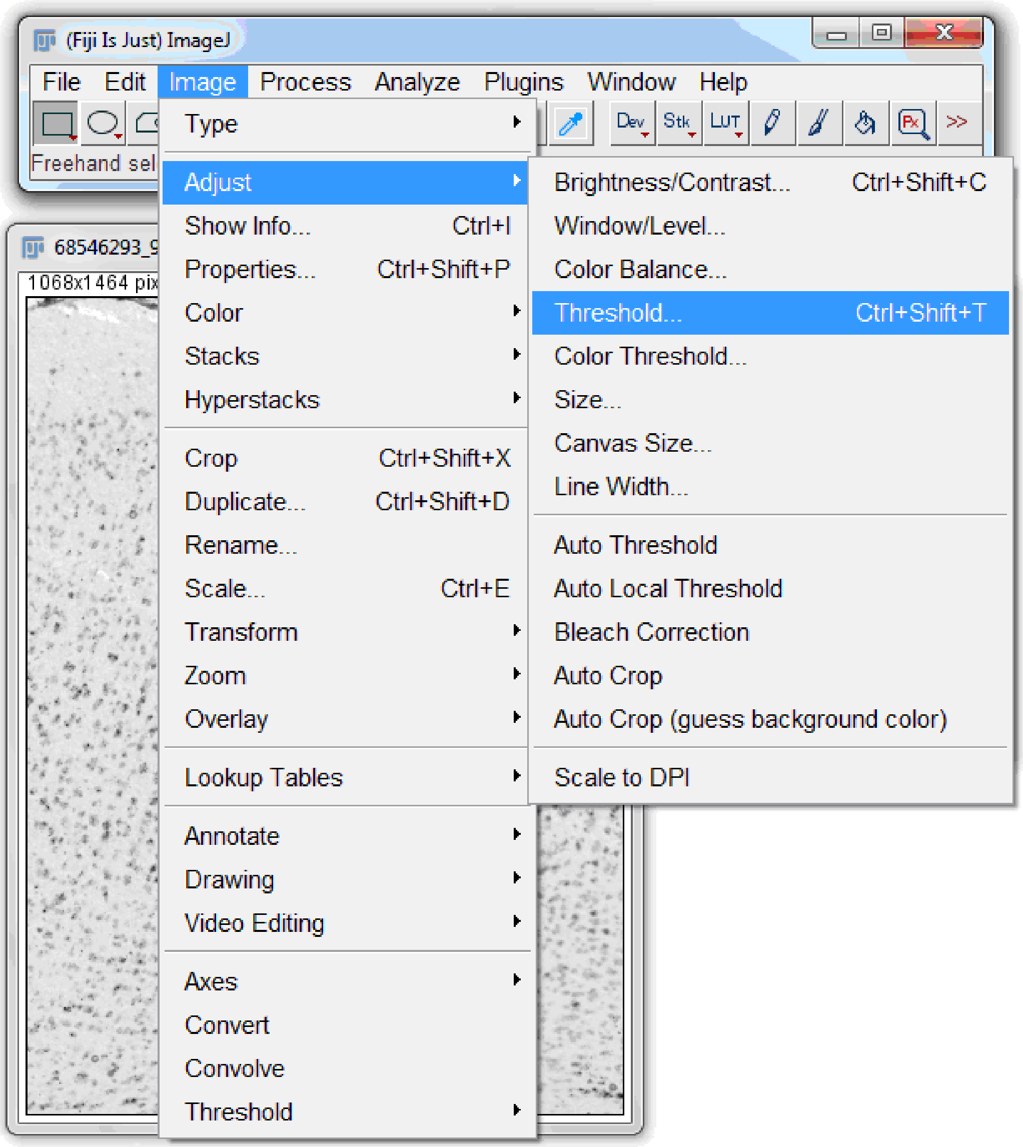

Thresholding image: some images may need robust thresholding (see help files in Fiji). Click Apply.

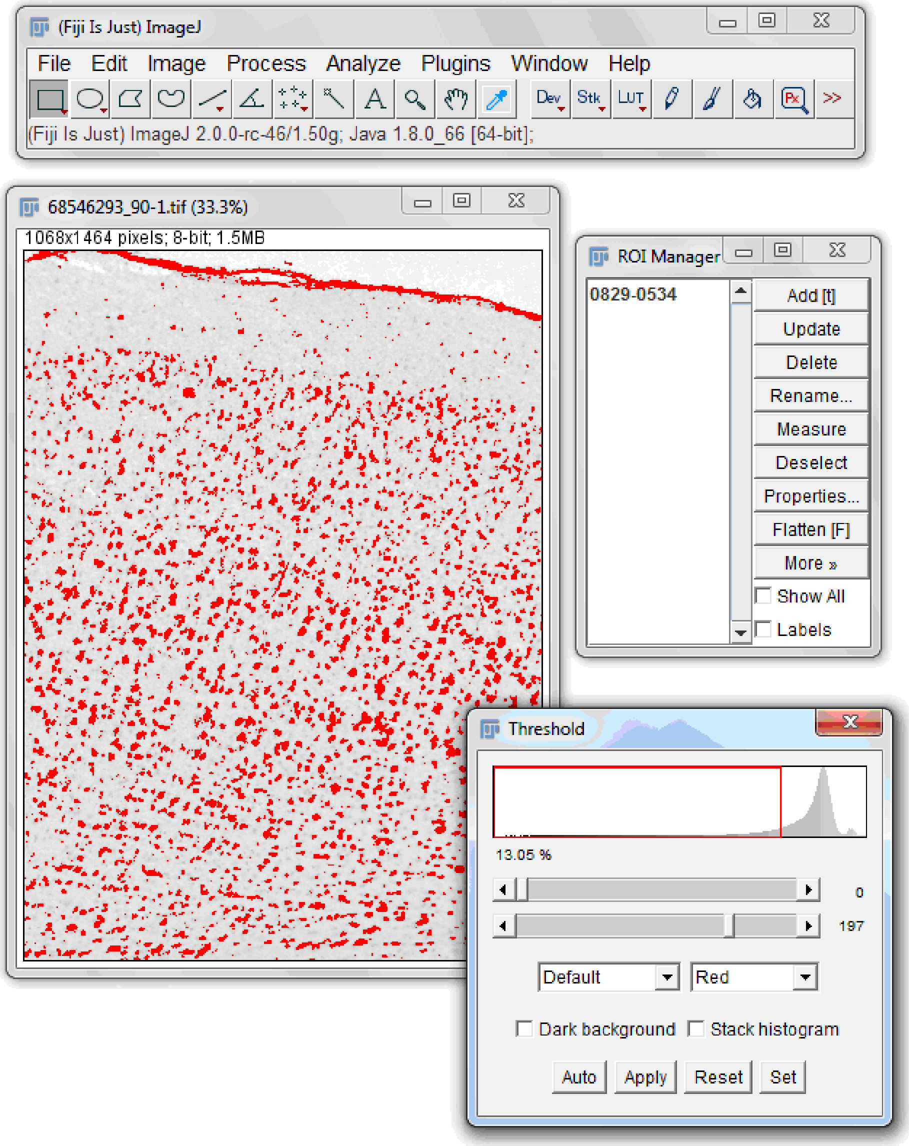

Thresholded binary image.

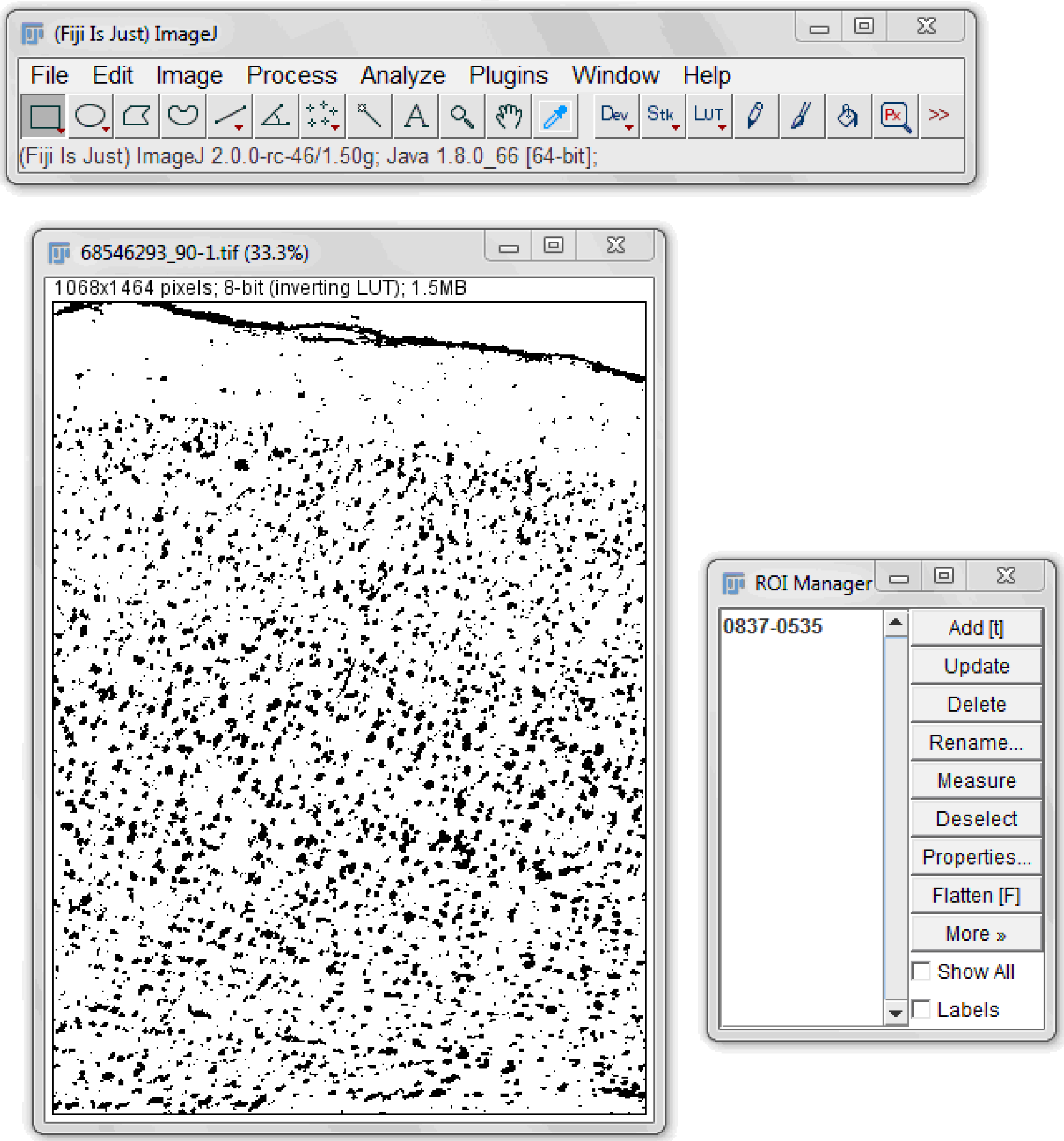

Go to Analyze>Set Measurements>Click on Mean gray value and Max and Min gray value) Click OK.

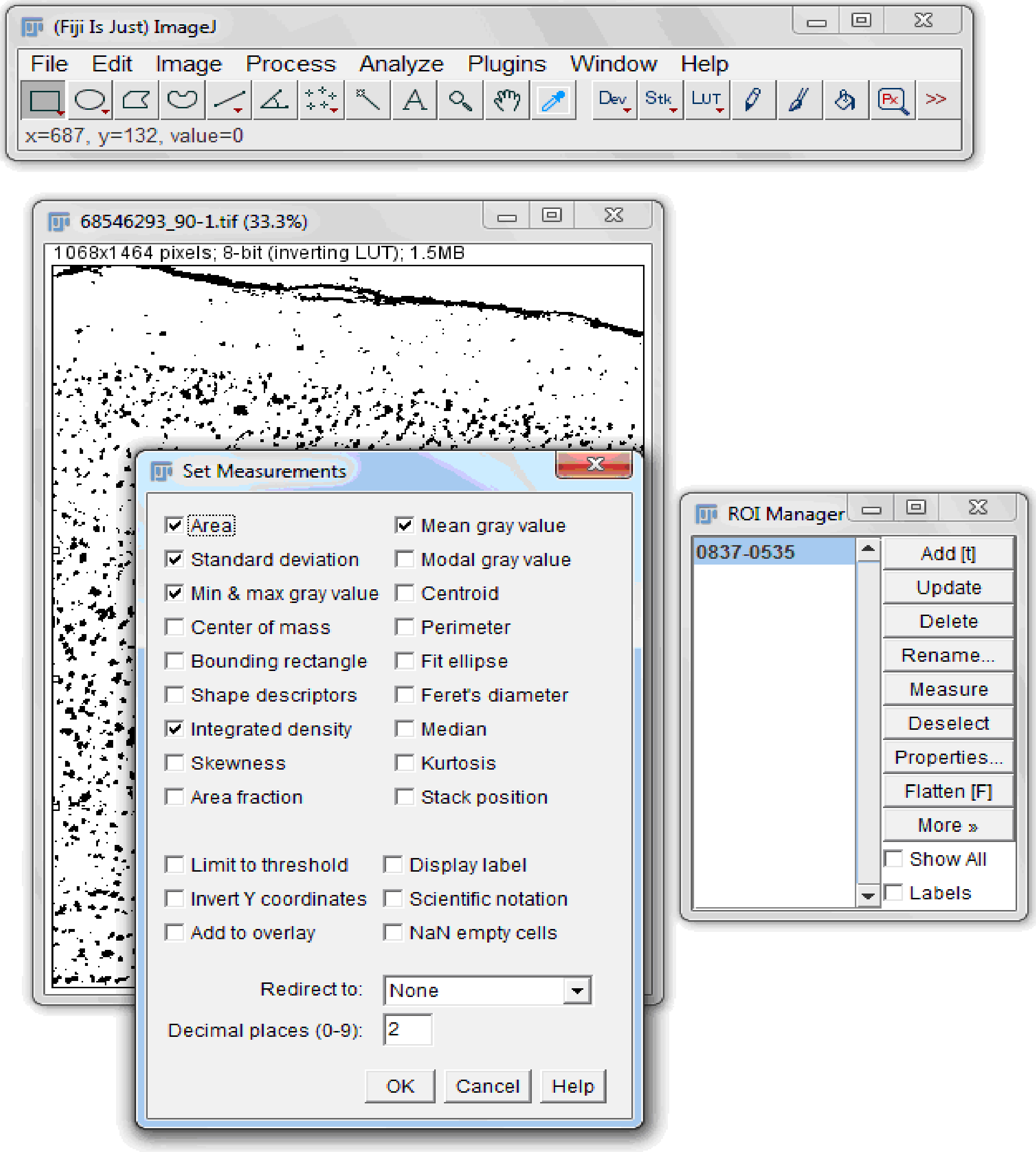

Select ROI traced from Nissl's image (shown in ROI Manager). Go to Analyze>Measure

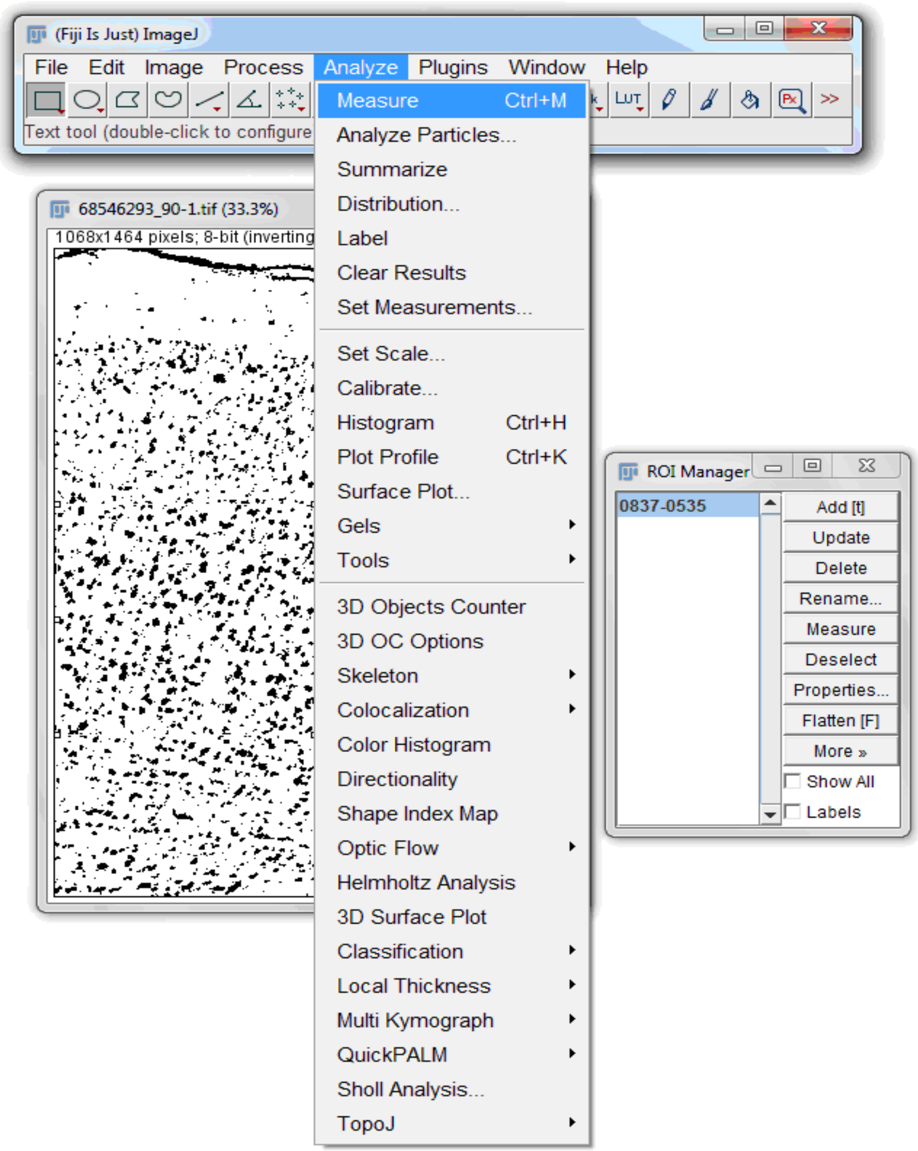

The mean gray value for all positive pixel intensities within the ROI will be shown in result window.

Go to Edit>Select all>copy and paste the results in Excel sheet.

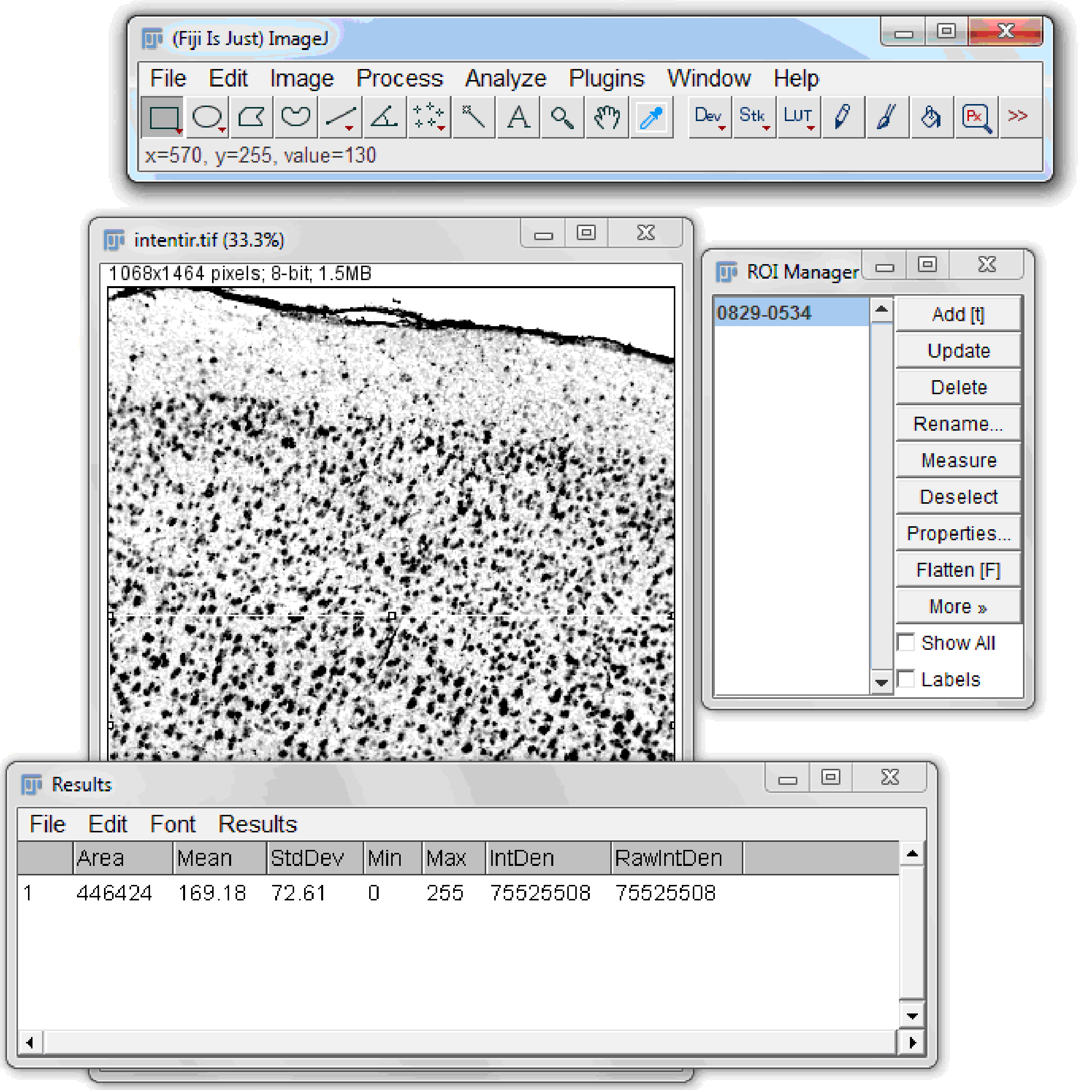

Example for Intensity (L) scoring

To determine expression levels, let's say of *Lgals8* in Cortex Layer 5 (L5) in parasagittal plane, where the ROI shows 'a' cells as blue and 'b' cells as green and 'c' as look yellow and 'd' cells in red, mean gray value associated with these pseudocolor pixels is ascertained from 8 bit binary images using Fiji: Image/Threshold and Analyse/Set Measurement(click on mean gray value and max and min gray value to visualize gray value range), follow Measure tool option to measure the mean gray values around all expressing pixels in the selected region.

For example, let's say, mean gray value was 169.18 (ranges from minimum 0 to maximum 255, shown in previous slide), then expression level (L1) according to mentioned intensity scale (from 0-7) will receive value of 2 (intensity calibration scale described in slide number 26). On performing similar measurements over 3 images, in one plane of section, an average Intensity (L, L= (L1+L2+L3)/3) can be obtained.

Similar exercise can be followed to yield average intensity in other planes of sagittal sectioning (mid/ lateral as the case maybe)

How to perform expression Density (D) measurements

On a 8 bit binary image, use ROI, duplicate image to show ROI in a separate window. Go to Process>Binary>Watershed (mark pixel outlines)

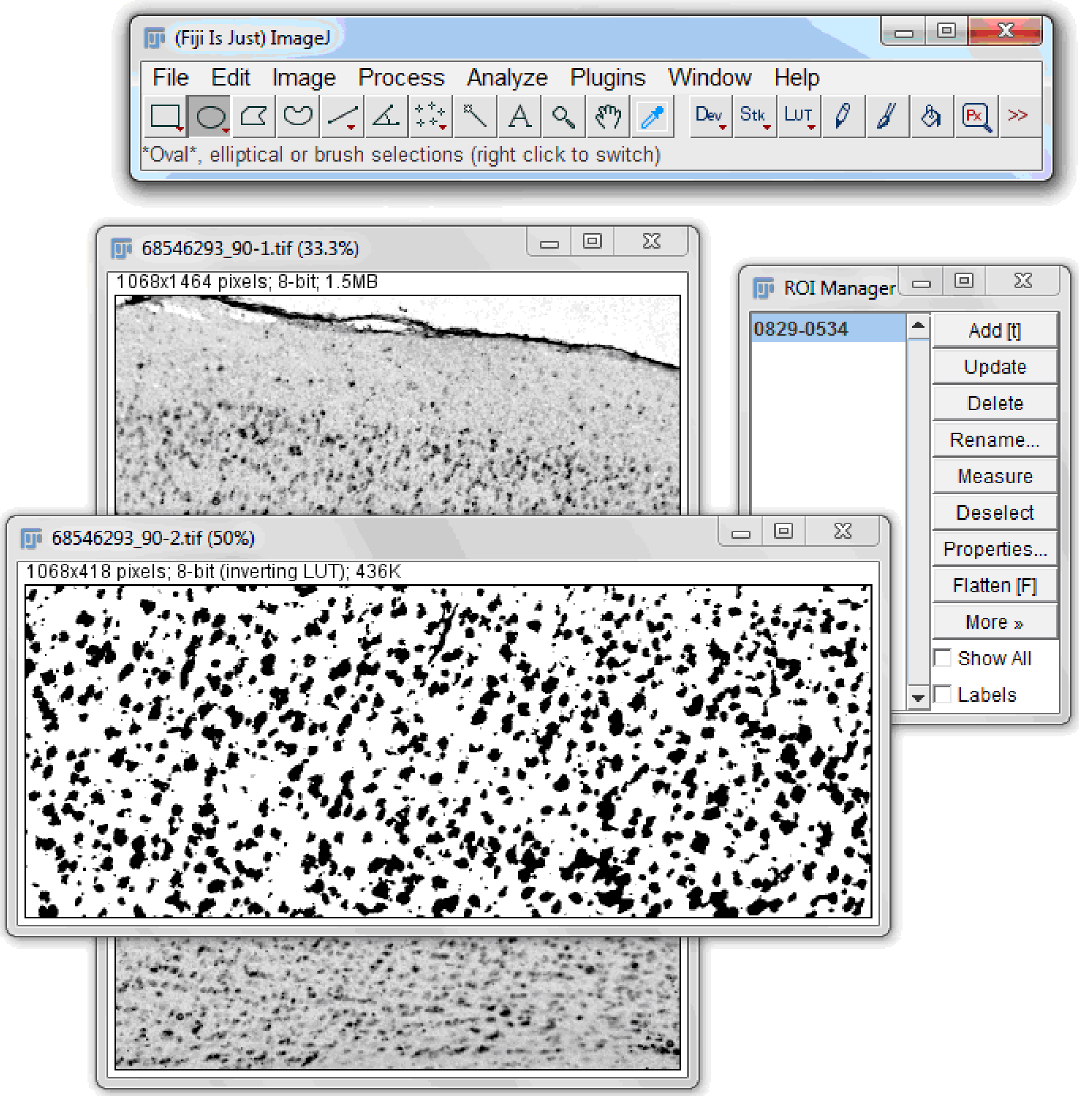

Go to Analyze>Analyze particles>Select-'outline' and 'display result' option.

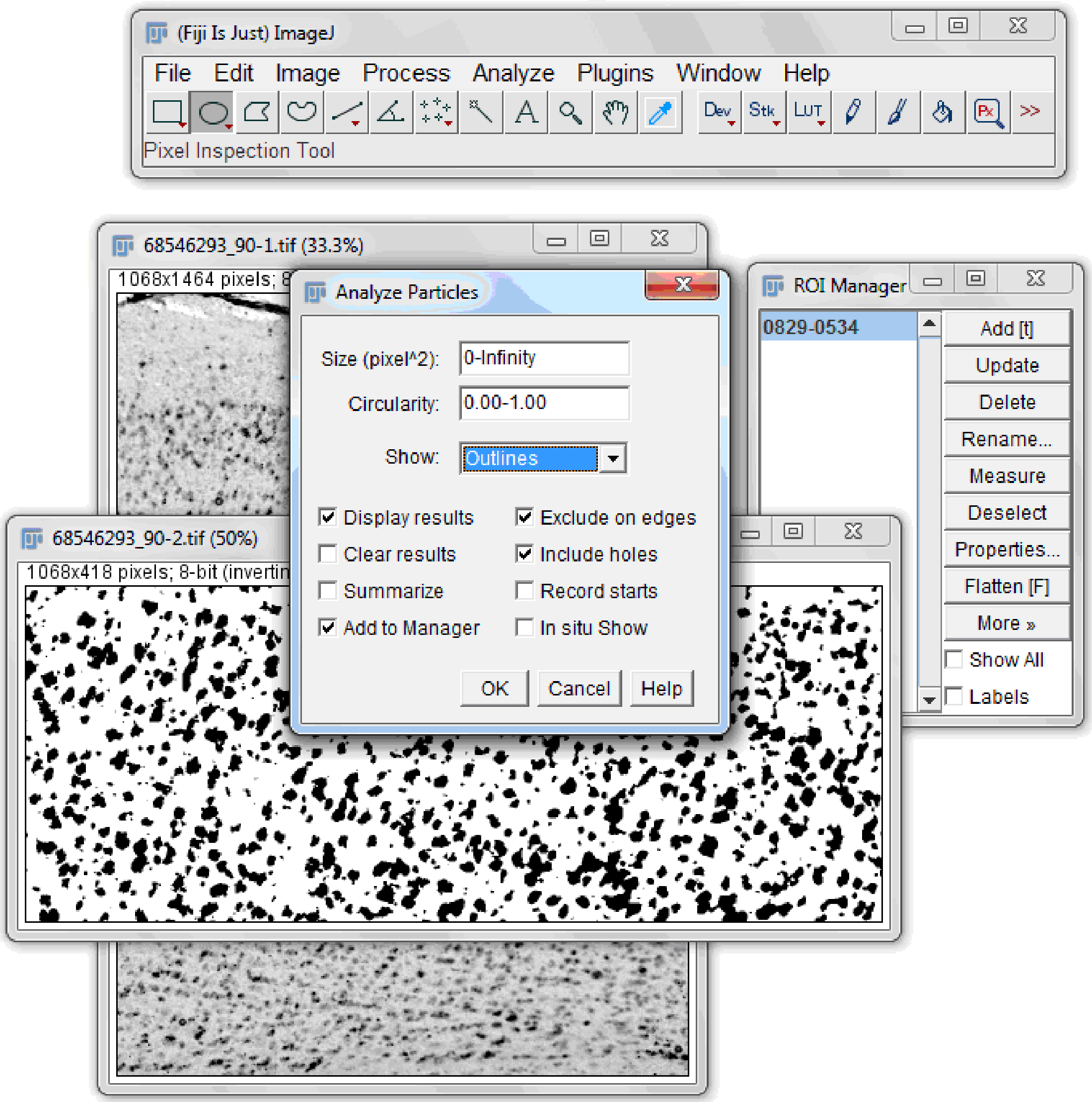

‘Results’ window will open to show areas counted with positive intensities. Also ‘measured areas’ outlines will be shown. Copy the results in Excel. Do the same for the corresponding Nissl’s image. Summate the areas of ISH and Nissl’s separately.

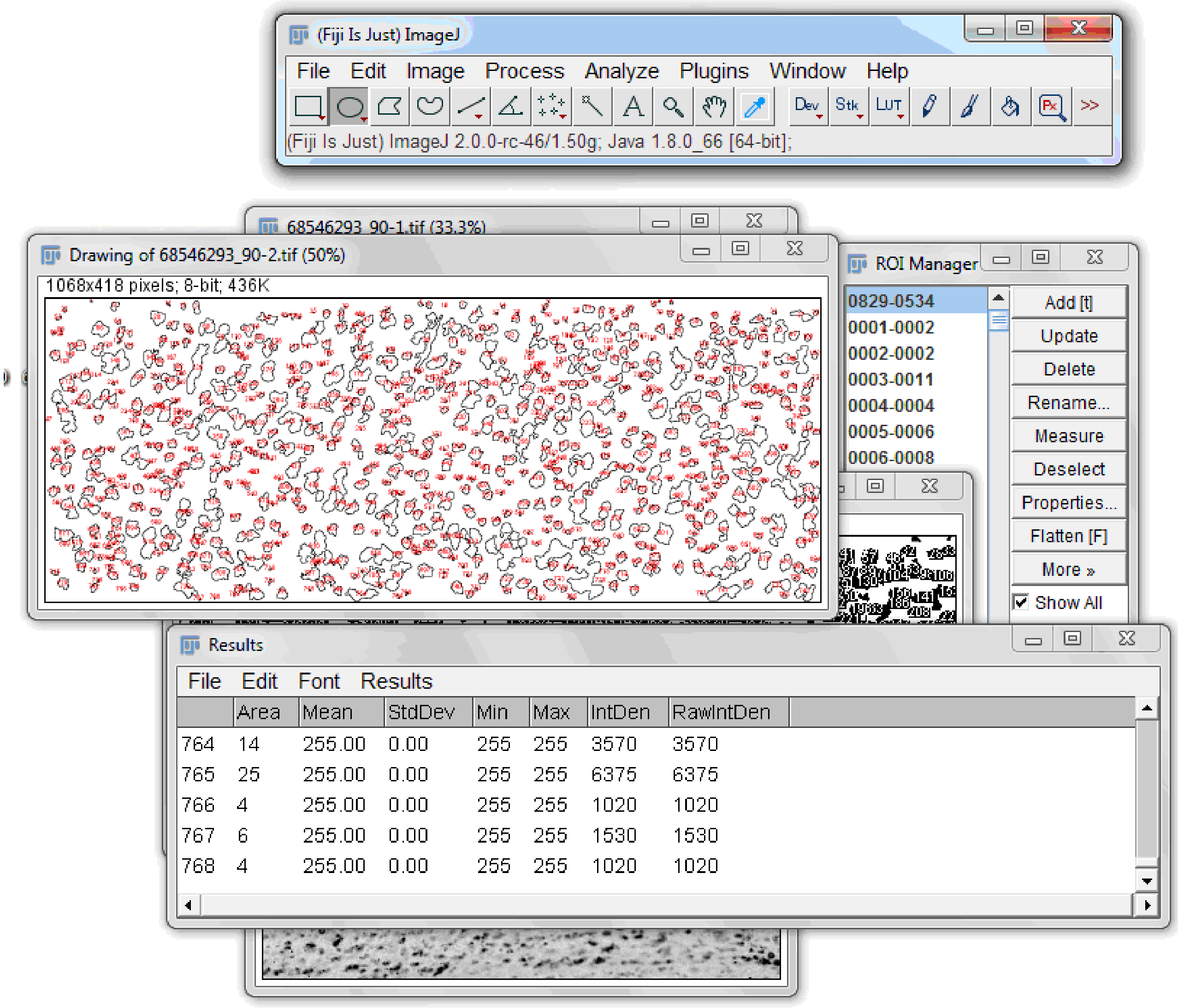

When 'sum of the areas' of all positive signals was divided by the area of the smallest pixel, that is 0.17um^2 (in Fiji) then the number of the total pixels for the transcript of interest and total number of pixels available in that area (through Nissl's image) can be independently obtained. From this 'density' in percentage can be calculated (% density Np, Np= [(sum of areas of all pixel intensities for a transcript in a ROI/0.17)/ (sum of areas of all pixel intensities for Nissl's image in the same ROI/0.17) *100].

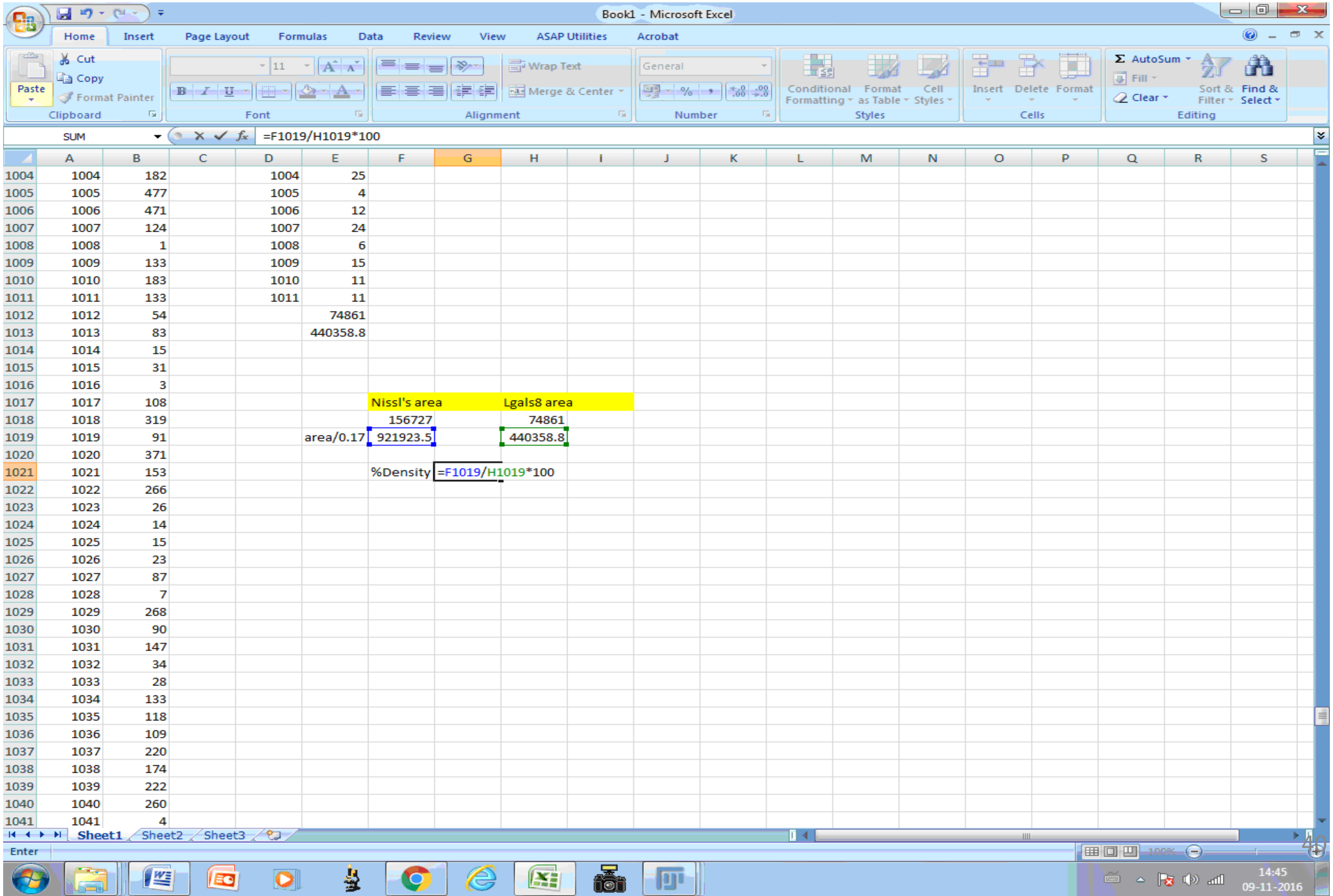

	A	B	C	D	E	F	G	H
1004	1004	182		1004	25			
1005	1005	477		1005	4			
1006	1006	471		1006	12			
1007	1007	124		1007	24			
1008	1008	1		1008	6			
1009	1009	133		1009	15			
1010	1010	183		1010	11			
1011	1011	133		1011	11			
1012	1012	54			74861			
1013	1013	83			440358.8			
1014	1014	15						
1015	1015	31						
1016	1016	3						
1017	1017	108				Nissl's area		Lgals8 area
1018	1018	319				156727		74861
1019	1019	91			area/0.17	921923.5		440358.8
1020	1020	371						
1021	1021	153				%Density	=F1019/H1019*100	
1022	1022	266						
1023	1023	26						
1024	1024	14						
1025	1025	15						
1026	1026	23						
1027	1027	87						
1028	1028	7						
1029	1029	268						
1030	1030	90						
1031	1031	147						
1032	1032	34						
1033	1033	28						
1034	1034	133						
1035	1035	118						
1036	1036	109						
1037	1037	220						
1038	1038	174						
1039	1039	222						
1040	1040	260						
1041	1041	4						

% density Np, Np= [(sum of areas of all pixel intensities for a transcript in a ROI/0.17)/ (sum of areas of all pixel intensities for Nissl's image in the same ROI) *100].
In this example, it was 47.76%

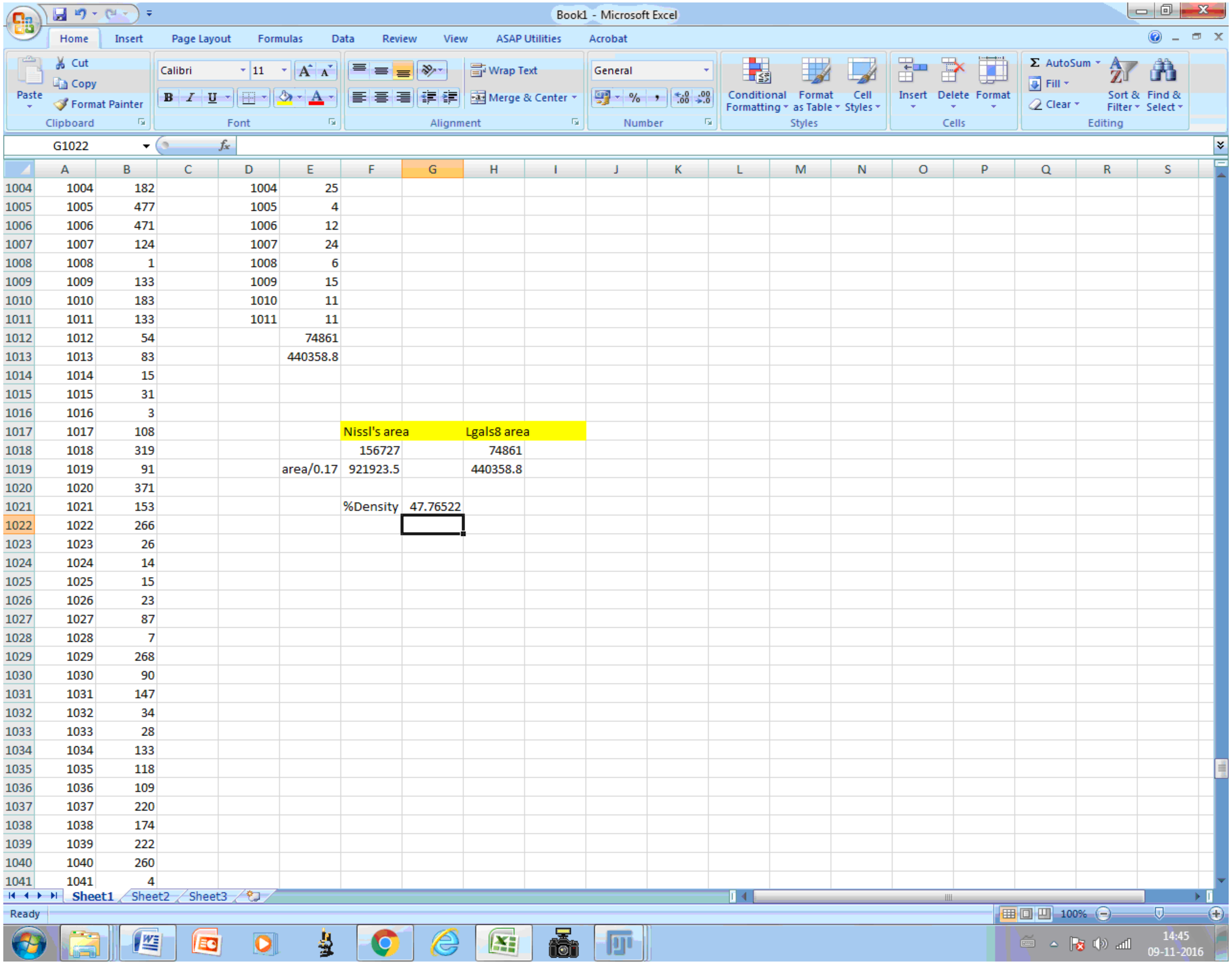

	A	B	C	D	E	F	G	H
1004	1004	182		1004	25			
1005	1005	477		1005	4			
1006	1006	471		1006	12			
1007	1007	124		1007	24			
1008	1008	1		1008	6			
1009	1009	133		1009	15			
1010	1010	183		1010	11			
1011	1011	133		1011	11			
1012	1012	54			74861			
1013	1013	83			440358.8			
1014	1014	15						
1015	1015	31						
1016	1016	3						
1017	1017	108				Nissl's area		Lgals8 area
1018	1018	319				156727		74861
1019	1019	91			area/0.17	921923.5		440358.8
1020	1020	371						
1021	1021	153				%Density	47.76522	
1022	1022	266						
1023	1023	26						
1024	1024	14						
1025	1025	15						
1026	1026	23						
1027	1027	87						
1028	1028	7						
1029	1029	268						
1030	1030	90						
1031	1031	147						
1032	1032	34						
1033	1033	28						
1034	1034	133						
1035	1035	118						
1036	1036	109						
1037	1037	220						
1038	1038	174						
1039	1039	222						
1040	1040	260						
1041	1041	4						

Example for Density (D) scoring

The calculated density (in percentage, Np) was further classified on the scale of 1 to 4:

a) scale of 1: 0-5% (*sparse distribution*);
b) scale of 2= 5-20% (*scattered distribution*);
c) scale of 3 = 20-70% (*medium distribution*) and
d) scale of 4 = >70% (*high/wide-spread distribution*).

For example, as shown in previous example, say in a *Lgals8* ISH image, ROI of parasagittal plane Cortex Layer 5 (CTX-L5) showed positive pixels 440358.8 while Nissl's image showed 9219235 positive pixels in the given ROI of a comparable sagittal section. Pixels from ISH image/Pixels from Nissl's image for the same ROI*100 gives the % density. Hence, in the given example, the area density is 47.76%

The percentage density receives a density (D1) value of 3 from the Density scale. On performing similar measurements over 3 images, in one plane of section, an average Density (D, D=(D1+D2+D3)/3) can be obtained.

Please note that this kind of analysis was done for each plane of sectioning using multiple magnification levels to accurately quantify the expression.

Also note that for more dense regions, a robust manual thresholding was performed using Fiji, *see* help files in Fiji).

How to perform expression Expression Factor (E) measurements

Expression Factor (E)

Different intensity level within an area were averaged (3 or more images) and multiplied with the average of density level within an area to provide an average expression factor (E) for one specific area or sub-structure in the mouse brain.

This can be further represented by the following relationship:

E= L X D,

where L is average of different intensities and D is an average of respective different densities.

Since there are 7 intensity levels and 4 density levels, the upper limit for average expression factor in individual subregions was 28 and the lower limit was 0. These expression factors were further assigned the following categories:

'*very low*' (where, $0<E<6$),
'*low*' (where, $6<E<11$),
'*moderate*' (where, $11<E<17$),
'*high*' (where, $17<E<22$) and
'*very high*' (where, $E>22$).

Example for Expression factor (E) calculation

Hence, using the average values of Intensity(L) and Density(D) functions from the previous example for CTX L5 in parasaggital plane ROI,
where say intensity (L) for 3 images is 2,2,1 and density (D) is: 3,3,3

then expression factor E [(L1xD1+L2xD2+L3xD3/3] will be 5.

So, in this example, expression factor for CTX L5 for *Lgals8* will be categorized as overall ' *very low*'(0<E<6).

END
